# Efficacy and safety of intranasal agents for the acute treatment of migraine: a systematic review and network meta-analysis

**DOI:** 10.1186/s10194-023-01662-6

**Published:** 2023-09-18

**Authors:** Guanglu Li, Shaojie Duan, Tiantian Zhu, Zhiying Ren, Hui Xia, Ziyao Wang, Lei Liu, Zunjing Liu

**Affiliations:** 1 Graduate School of Beijing, University of Chinese Medicine, Beijing, China; 2 Department of Neurology, China-Japan Friendship Hospital, Beijing, China; 3 Department of Geriatrics, Taizhou Central Hospital (Taizhou University Hospital), Taizhou, China; 4 Beijing Tiantan Hospital, Capital Medical University, Beijing, China; 5 Department of Neurology, Peking University People’s Hospital, Beijing, China

**Keywords:** Migraine, Acute treatment, Intranasal agents, Network meta-analysis

## Abstract

**Background:**

Intranasal agents may be ideal for the treatment of migraine patients. Many new acute intranasal-specific therapies have been developed, but few of them have been directly compared. The aim of this network meta-analysis (NMA) was to compare the efficacy and safety of various intranasal agents for the treatment of acute migraine in adult patients.

**Methods:**

The Cochrane Register of Controlled Trials, Embase, and PubMed were searched from inception to 15 August 2023. Randomized controlled trials (RCTs) using intranasal agents (no restrictions on dose, formulation, dosing regimen or timing of the first dose) to treat adult patients with acute migraine were included. The primary efficacy endpoint was pain freedom at 2 h, and the primary safety endpoint was adverse events (AEs). The analysis process followed the Preferred Reporting Items for Systematic Reviews and Meta-Analyses (PRISMA) guidelines.

**Results:**

Nineteen studies (21 RCTs, 9738 participants) were included. Compared to the placebo, 5 mg of zolmitriptan using a conventional liquid nasal spray device was the most effective for pain freedom at 2 h [odds ratio (OR): 4.67, 95% confidence interval (CI): 3.43 to 6.43] and 24 h (OR: 5.49, 95% CI: 3.58 to 8.42) among all the interventions. Butorphanol nasal spray 1 mg was the most effective (OR: 8.62, 95% CI: 1.11 to 66.92) for pain freedom at 1 h, but with low-quality evidence. DFN-02 presented the highest freedom from nausea (OR: 4.95, 95% CI: 1.29 to 19.01) and phonophobia (OR: 5.36, 95% CI: 1.67 to 17.22) at 2 h, albeit with lower odds of achieving complete pain freedom. ROX-828 showed the highest improvement in freedom from photophobia at 2 h (OR: 4.03, 95% CI: 1.66 to 9.81). Dihydroergotamine nasal spray was significantly associated with the highest risk of AEs (OR: 9.65, 95% CI: 4.39 to 21.22) and was not recommended for routine use. Zavegepant nasal spray demonstrated the lowest risk of AEs (OR: 2.04, 95% CI: 1.37 to 3.03). The results of sensitivity analyses for the primary endpoints (pain freedom at 2 h and AEs) were generally consistent with those of the base case model.

**Conclusions:**

Compared with other new intranasal-specific therapies in treating migraine attacks, zolmitriptan nasal spray 5 mg was the most effective agent for pain freedom at 2 h. Zavegepant nasal spray 10 mg had the fewest adverse side effects.

**Supplementary Information:**

The online version contains supplementary material available at 10.1186/s10194-023-01662-6.

## Introduction

Migraine is a common trigeminal neurovascular disorder with typical symptoms of recurring [1, 2], mostly one-sided, often highly disabling attacks of moderate to severe throbbing headaches [3, 4], often mixed with nausea, vomiting, photophobia, phonophobia, blurred vision and other variable physical, mental and psychological signs and symptoms for 4–72 h [5]. According to the data from the Global Burden of Diseases, Injuries, and Risk Factors (GBD), migraine is the second most prevalent neurological disorder [6, 7] and the second leading cause of years lived with disability (YLD) after low back pain but takes first place in young women [8, 9]. In addition, migraine is the top cause of disability-adjusted life years (DALYs) in young women [9]. Based on GBD modelling of prevalence, almost 1.04 billion people are estimated to suffer from migraines, with a global age-standardized prevalence of approximately 14.4%. The financial cost of migraine is also a societal concern. Migraines cost nearly $17 billion annually in the United States [10], while in Europe, the direct, indirect, and social costs are estimated at €27 billion per year [11]. Headache-related disability, high comorbidity, financial stress, and absence of family roles impose a significant personal burden on individuals with migraine.

Migraine management includes acute treatment and preventive treatment. Acute treatment aims to provide rapid and sustained relief of headaches and other related symptoms, restore functional ability, and minimize rescue medication use and adverse events (AEs). Choosing the appropriate medication, dose, route of administration, and early dosing can improve acute treatment outcomes. Stratified care and step-care are two principles of acute migraine treatment. Stratified care means selecting treatment medications based on pain intensity. Analgesics and nonsteroidal anti-inflammatory drugs (NSAIDs) can effectively treat mild or moderate attacks. Triptans are the first choice for moderate to severe acute migraine [12, 13]. Step-care means using simple analgesics and only stepping up to a migraine-specific medication if the pain progresses [14]. According to the American Headache Society, evidence-based proposals for acute pharmacologic treatment of migraine, triptans, ergot derivatives, NSAIDs, opioids, and combination medications are recommended for the treatment of migraine [13].

Oral tablets are the most common options for patients due to convenience and comfort [15]. Although oral therapies are the most commonly prescribed, the onset of action for oral tablets is relatively slow compared to other formulations [16]. Oral tablets may be difficult for patients with nausea and vomiting to ingest [17]. In a survey of 500 people with migraine, 30.5% of patients with nausea and 42.2% with vomiting reported that those accompanying symptoms hindered their ability to take oral medications [17, 18]. Patients with migraine often experience reduced gastrointestinal motility, which may delay medication absorption and result in a slowed or inconsistent treatment response [19]. Nonoral formulations offer an alternative to oral treatment. Subcutaneous (SC) methods work the fastest but are associated with more common side effects [20–22]. SC sumatriptan has long been considered the most rapidly effective triptan [23], reaching the maximum concentration (t_max_) in 12 min, having an onset of action of 10 min, and relieving headache in 82% of patients 2 h post dose [24, 25]. However, due to the suboptimal tolerability profile, fewer than 10% of eligible migraine patients choose it to treat their headache [26]. The most common AE with SC sumatriptan is burning or stinging at the administration site, which occurs in approximately 60% of patients [27]. Many patients (42%) experience “triptan sensations” [24, 25], such as warmth/heat, tightness/pressure, tingling, flushing, and feelings of heaviness or pressure in areas such as the face. Intravenous injection is not a routinely recommended formulation for migraine attack [3, 28, 29] and is primarily used for status migraines and severe headache attacks not responding to acute treatment at home [30]. For patients presenting to the emergency department and for migraine patients requiring admission, intravenous injection is available but is also frequently difficult for episodic migraine sufferers to obtain on a timely basis. Intranasal formulations may be an ideal alternative for migraine patients with significant nausea, vomiting, gastroparesis, rapidly progressing headache, a high recurrence rate, an inadequate response to oral therapy, or more invasive parenteral therapies.

Although multiple intranasal medications have shown positive efficacy and safety for the acute treatment of migraine in a series of randomized controlled trials (RCTs), as most of the studies were placebo-controlled trials, according to our search, the drugs were not directly compared. The relative efficacy and safety of each nasal spray device were uncertain. Without direct comparisons, network meta-analysis (NMA) provides a way to perform multiple comparisons simultaneously in a single analysis as evidence for clinical practice. Therefore, we conducted an NMA to systematically assess and rank the relative effectiveness, safety, and acceptability of various intranasal medications for the acute treatment of migraine in adult patients. The results of this NMA will be useful for clinicians.

## Methods

### General guidelines applied

**Fig. 1:**
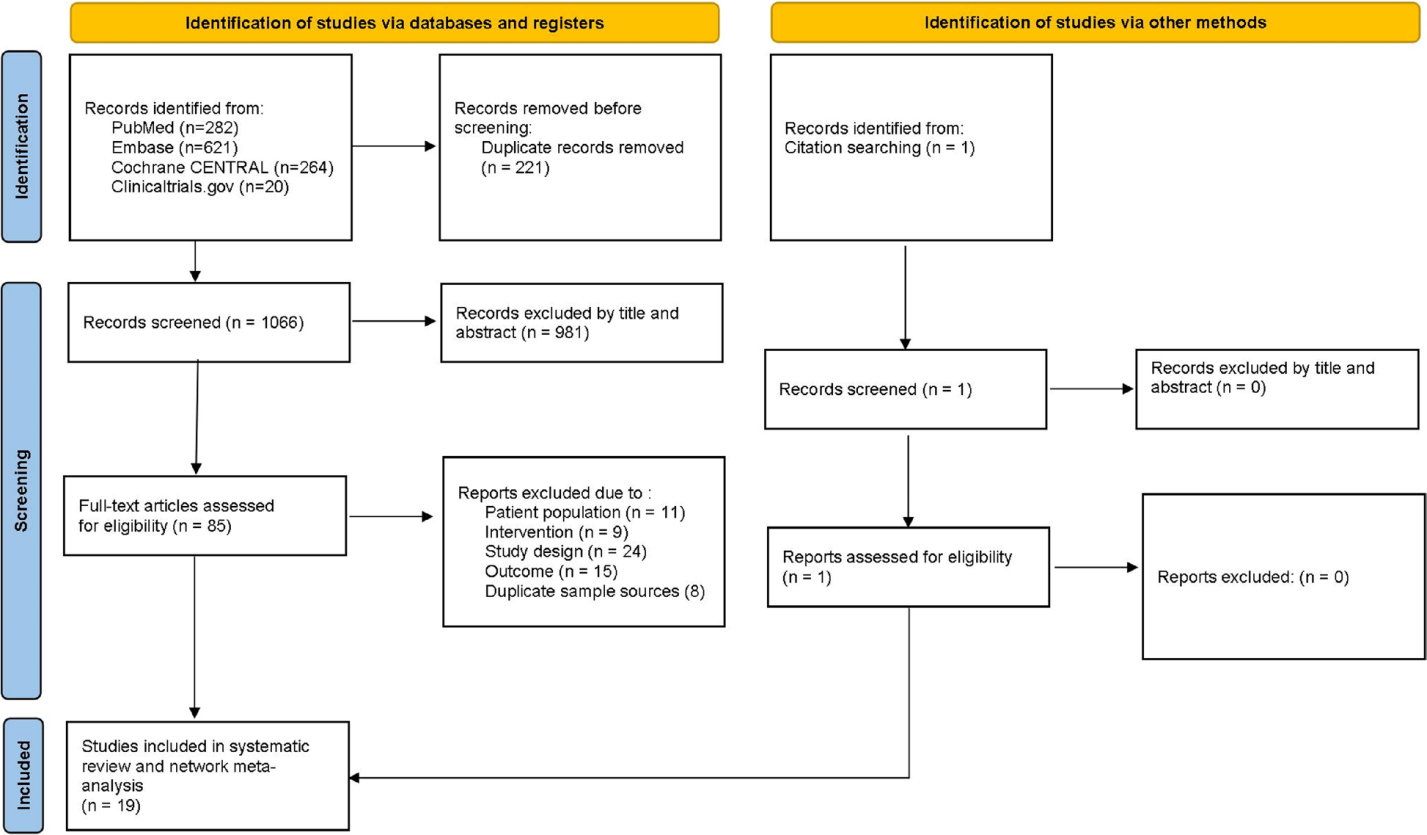
Flow Chart of the network meta-analysis procedure. A total of 21 trials were included because 2 studies provided the results of 2 trials each

This systematic review and meta-analysis followed the recommendations of the Preferred Reporting Items for Systematic Reviews and Meta-analyses (PRISMA) reporting guideline checklist [31]. The PRISMA flowchart of the screened studies is shown in Fig. 1. Specific PRISMA checklists of the current meta-analysis are reported in eAppendix 1.

### Literature search

This systematic review and meta-analysis identified intranasal medications used to treat chronic or episodic migraine patients with or without aura and was registered in the International Prospective Register of Systematic Reviews (PROSPERO: CRD42023396291). First, we searched the Cochrane Register of Controlled Trials and MEDLINE via PubMed and Embase databases, with language restrictions in English, from inception until August 15, 2023, to search for RCTs of intranasal pharmacologic agents in the acute treatment of migraine. We applied a combination of keywords and text words related to migraine and intranasal pharmacologic agents. Then, we combined them with validated screening tools recommended by the Harvard Countway Library for randomized controlled clinical trials. Each database uses a specific search strategy and can be found in eAppendix 2. Registries of clinicaltrials.gov were also searched to identify ongoing trials. We additionally conducted searches from existing pairwise meta-analyses and the reference lists of review articles to complement our further trials [32–35]. Two authors operated the literature search process independently (LGL, DSJ).

### Inclusion and exclusion criteria

To increase the reliability of the current NMA, studies needed to meet the following PICOS criteria and had been peer-reviewed and formally published.

1. Population (P): Participants were required to be diagnosed with episodic and chronic migraine patients (with or without aura) for 18 years and older based on the International Classification of Headache Disorders criteria (ICHD) system, or the ICHD operating at the time of the study.
2. Intervention (I): We examined all types of intranasal agents currently available for migraine-specific acute treatments. There were no restrictions on the dose, formulation (e.g., liquid spray, powder), dosing regimen (e.g., single dose versus optional second dose of the study medication or rescue medication) or timing of the study medication (e.g., first dose of the study medication for moderate to severe headache attacks or first dose for mild headache attacks). However, we only extracted doses recommended by guidelines [28] or previous studies to limit the number of intervention arms.
3. Comparator (C): We included only RCTs that compared at least one pharmacologic agent with placebo or that performed direct comparisons of at least two pharmacologic agents but applied a placebo in the study design.
4. Outcome (O): The study had to report at least one clinical outcome indicator that we were concerned about.
5. Study design (S): We only included RCTs with human participants that were fully published in English.

The exclusion criteria were as follows: (1) no target outcome that interested us, (2) nonhuman clinical trials; (3) no inclusion of adult individuals with migraine; (4)studies not published in peer-reviewed journals or studies that were post hoc or secondary analyses; (5) crossover studies, excluded except when the results of the first phase were given separately; (6) no use of placebo; and (7) studies involving cluster, tension, menstrual migraine or episodic and chronic migraine associated with other neurological disorders. For duplicate publications (i.e., the same set of sources had been used by multiple studies), we only included studies with larger samples and more information.

### Study screening

The extraction process for all returned study data followed a standardized form. After the initial search, one pair of reviewers (GL Li, SJ Duan) independently performed the following operations: removing duplicates, reviewing the titles and abstracts of all identified citations for primary screening, and retrieving and screening full-text papers according to eligibility criteria. Disagreements were resolved through discussion and, if necessary, by a third senior author (L Liu). The Endnote Literature Management platform was used for study selection and screening.

### Primary and secondary outcome measures

The guidelines recommended to determine the effectiveness for acute treatment of migraine should be either pain freedom at 2 h or freedom from the most bothersome symptom (MBS) at 2 h as a coprimary endpoint [36]. We followed the International Headache Society (IHS) recommendations and chose pain freedom (defined as the absence of pain based on a 4-point global scale) 2 h before using any rescue medication as our primary efficacy outcome. We did not choose freedom from the MBS at 2 h because few trials among the initially included studies reported it. Relevant secondary efficacy endpoints of the intranasal medications included freedom from migraine symptoms at 2 h, with such symptoms being photophobia, phonophobia, and nausea, and were also evaluated to provide a more accurate option for patients planning to use intranasal agents for migraine. In addition, we chose pain freedom at 1 h and sustained pain freedom to 24 h as secondary efficacy outcome measures to assess the early and sustained migraine treatment response.

The primary outcomes for safety and tolerability were any recorded AEs within 48 h post-dose, any serious adverse events (SAEs), withdrawal due to AEs, and the frequency of the most common AEs.

### Data collection and quality assessment

For each included study based on eligibility criteria, two reviewers (GL Li, SJ Duan) independently performed the data extraction with a predesigned Excel spreadsheet, which included the study title, first author name, publication year, participants’ baseline data characteristics, sample size, details on each study arm/pharmacological intervention (dose, frequency, administration route, duration of intervention), efficacy outcomes and safety outcomes data, and information for the assessment of the risk of bias. A third reviewer (L Liu) checked the consistency and accuracy of all extracted data. Any discrepancies in evaluating these data were resolved by discussion or consultation. A third author (L Liu) would evaluate those data that could not be resolved until a consensus was reached. Two reviewers (GL Li, SJ Duan) independently assessed each included study critically using a standardized table according to the Cochrane Risk of Bias version 2 (RoB2) for RCTs [37].

### Statistical analysis

We performed all NMA analyses using R (version 3.2.2) to combine direct and indirect evidence for migraine. For each primary and secondary outcome measure, we estimated the odds ratio (OR) with a 95% credible interval (CI) using a frequentist random effects model because of the heterogeneity and relatively small number of studies we included [38, 39]. We used the frequentist theory model with the mvmeta command to compare the effect size (ES) among studies with the same treatments. All comparisons were two-tailed, and a p value cut-off point of 0.05 denoted statistical significance. Heterogeneity among the included studies was evaluated using the tau value and the heterogeneity statistic I^2^. The Tau value is the estimated standard deviation of the effect across the included studies [40]. I^2^ values of less than 50% indicate that the heterogeneity may not be significant; a value higher than 50% may represent substantial heterogeneity. NMA relies on the transitivity assumption to estimate indirect treatment effects. We conducted a statistical evaluation of inconsistency. A loop-specific approach and the node-splitting method [41] were used to assess the potential local inconsistency of the model. The design-by-treatment model [40] was applied to evaluate global inconsistency among the whole NMA.

One of the advantages of NMA is that it allows for the ranking of interventions. Several ranking metrics have been proposed to present NMA results. For the Bayesian framework, competing treatments can be reported by rank probabilities (i.e., the probability of being at each possible rank, from best to worst), the mean/median rank, or the surface under the cumulative ranking curve (SUCRA) [42]. For the frequentist framework, authors can use the P score. Ranking of interventions based on the rank probabilities should be discouraged. This approach does not account for the uncertainty in relative effect estimates and relative ranking and can spuriously give higher ranks to treatments for which little evidence is available [43, 44]. The cumulative ranking probabilities are more appropriate to present treatment rankings and their uncertainty [44]. SUCRA is one such summarization ranking metric proposed by Salanti et al. [44]. It is calculated by averaging cumulative rank probabilities and transforms the mean rank of a treatment to a value between 0 and 1. The advantage of SUCRA over the mean rank is that it has a common range from 0 to 1, facilitating consistent interpretation of different NMAs [45]. SUCRA values of 1 indicate that the treatment might be the best and 0 the worst [44].

We performed sensitivity analyses in three manners on the availability of data within the networks for the primary efficacy endpoint (pain freedom after 2 h) and the safety endpoint (AEs):

- Sensitivity analysis 1-exclusion of studies with a high risk of bias.
- Sensitivity analysis 2-exclusion of studies prohibiting and not reporting the use of prophylactic medication.
- Sensitivity analysis 3-exclusion of studies reporting multiple headache episodes in a single patient.

Confidence in Network Meta-Analysis (CINeMA) [46] was used to assess the quality of evidence for the outcome indicators of the NMA. CINeMA is an approach for determining confidence in the results of an NMA broadly based on the Grading of Recommendations Assessment, Development, and Evaluation (GRADE). We used a freely available, user-friendly online CINeMA web application [47] to evaluate confidence in the results from NMA. Finally, comparative-adjusted funnel plots and Egger regression [48] were used to assess potential minor study effects and publication bias.

## Results

### Search results and study characteristics

Across the NMA, 1187 database records were identified from the initial literature search for the review stage. Additional literature was searched through citation searching. After removing 221 duplicates,1066 papers were initially included in the review; of these, 981 were eliminated by reviewing the titles and abstracts, and 85 were further screened by examining the full text. Based on the eligibility, a total of 19 studies [49–67] (21 RCTs comparing 12 pharmacologic treatments with each other or with a placebo) fulfilling the inclusion criteria were included in the final NMA. PRISMA diagrams of the screened studies are given in Fig. 1.

**Fig. 2:**
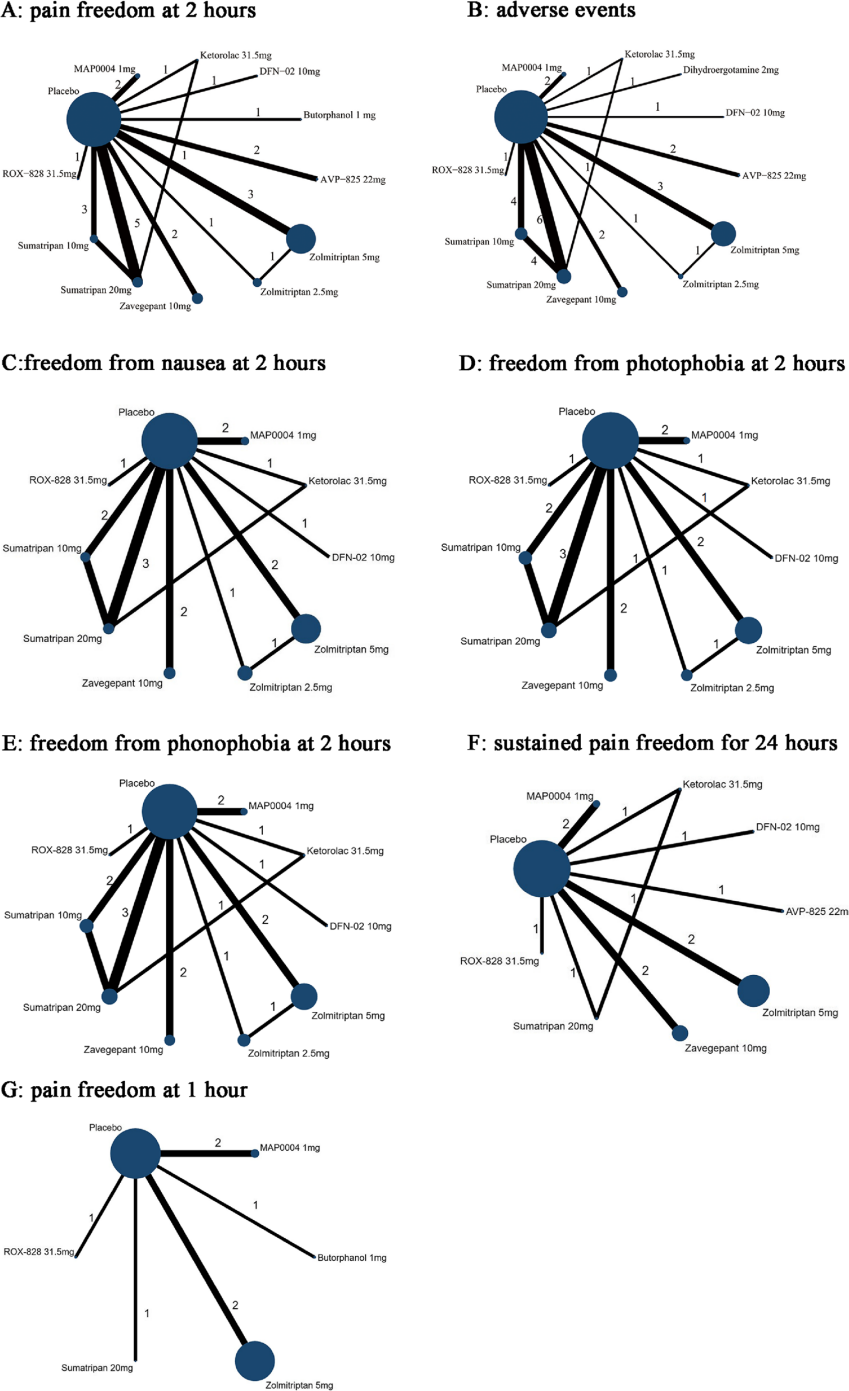
Network plot for primary outcomes and secondary outcomes. The lines between nodes represent direct comparisons in various trials, and each circle's size is proportional to the population involved in each specific treatment. The thickness of the lines is proportional to the number of trials connected to the network. DFN-02, sumatriptan nose spray 10 mg with a permeation enhancer; AVP-825, a drug-device combination of 22mg sumatriptan powder; MAP0004, a dihydroergotamine inhaler; ROX-828, ketorolac 31.5mg with 6% of lidocaine

We enrolled five classes of antimigraine acute medicines, including triptans (sumatriptan nasal spray 10, 20 mg; zolmitriptan nasal spray 2.5, 5 mg; DFN-02 nasal spray (which contains sumatriptan 10 mg with a permeation enhancer); AVP-825 (a drug-device combination of 22 mg sumatriptan powder), gepants (zavegepant nasal spray 10 mg), NSAIDs (ketorolac nasal spray 31.5 mg and ROX-828, a ketorolac 31.5 mg with 6% lidocaine), ergot derivatives (dihydroergotamine nasal spray 2 mg and MAP0004, a dihydroergotamine inhaler 1 mg) and opioids (butorphanol nasal spray 1 mg). The network graphs are shown in Fig. 2.

All included studies were published between 1994 and 2023 and conducted in several countries (e.g., the USA, Czech Republic, France, Germany). Each included study was similar, and the inclusion criteria were consistent with the current clinical guidelines. A total of 9,738 randomized participants were involved in the analysis. 32% (6/19 items) of studies did not allow concurrent use of prophylactic medication. However, 58% (11/19 items) of the studies allowed this use, provided the patients needed a stable dose for 30–90 days prior to the screening visit, and the dose did not change during the study. Additionally, two studies did not report this aspect. 63% (12/19 items) of studies included migraine patients with or without aura; the remaining studies were unspecified. The baseline demographic characteristics and headache features of the 19 studies included in the NMA are given in eTable 1. We labelled the heterogeneity statistic I^2^ of each outcome in the upper right of the forest plot and only detected substantial heterogeneity in the endpoint of AEs (I^2^ = 55.5%).

Participants used zolmitriptan nasal spray across two and three migraine attacks in papers published by Dodick et al. in 2005 [59] and Charlesworth et al. in 2003 [61]. For the crossover trials, we only extracted the first phase results in papers published by Rao et al. in 2016 [52]. In sensitivity analysis 3, we excluded these three studies and combined the ES again. In most of the remaining studies, patients used the intervention for a single migraine attack.

### Efficacy

#### Primary efficacy outcome: pain freedom at 2 h

**Fig. 3:**
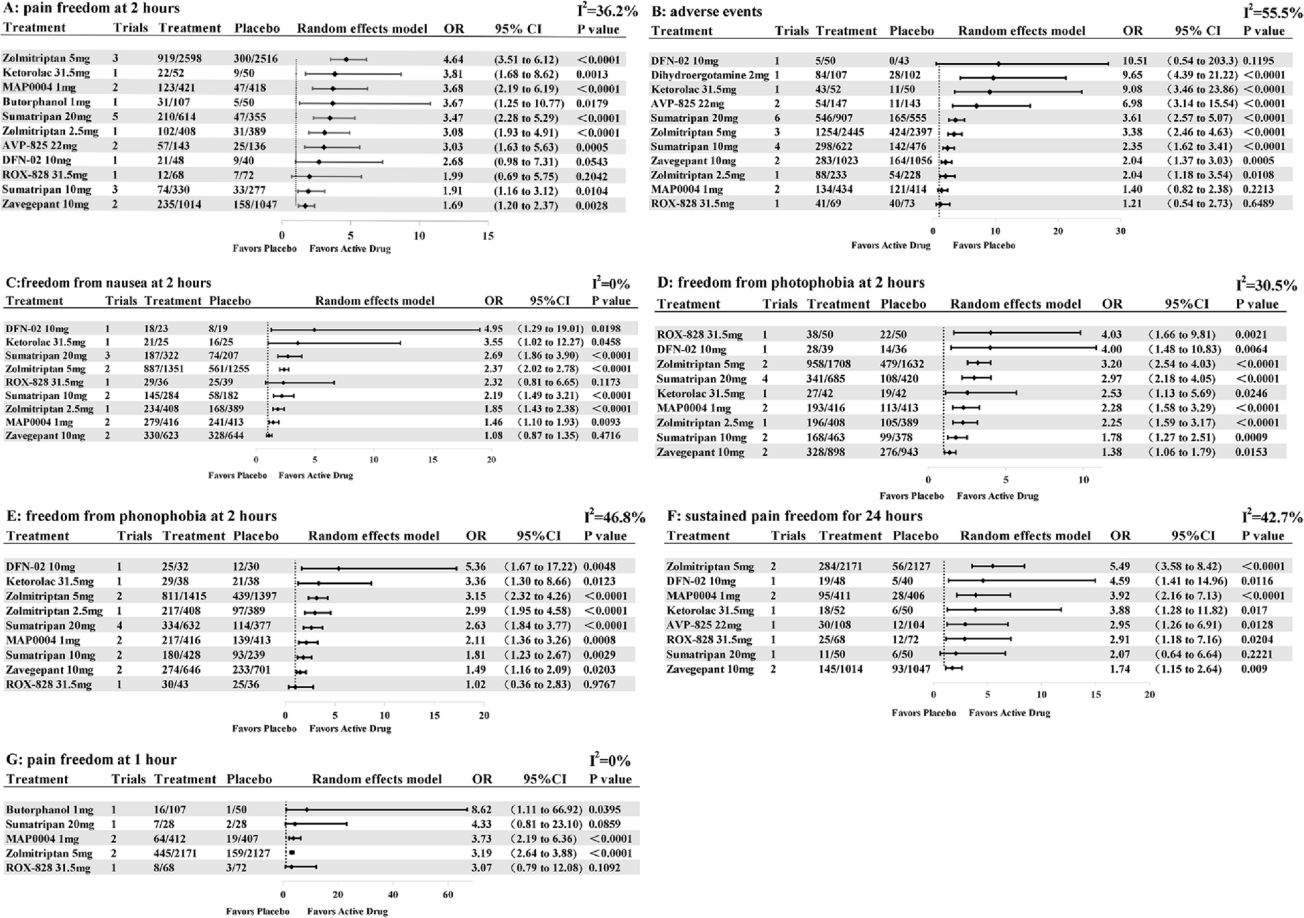
Forest plot for primary outcomes and secondary outcomes. The forest plot was based on a random-effects model. The data behind the drug names indicate the following: Trials, number of trials examining the current active drug; Treatment/Placebo, number of patients with events/number of patients in which the drug was examined in these trials; OR, odds ratio; CI, credible interval. 95% CI that did not contain one and a p-value cutoff point of 0.05 was considered statistically significant. I^2^ values of less than 50% indicate that heterogeneity may not be significant; a value higher than 50% may represent substantial heterogeneity. For the effectiveness endpoint, results to the left of 1 favor placebo, to the right favor intervention, result in adverse events was the opposite. DFN-02, sumatriptan nose spray 10 mg with a permeation enhancer; AVP-825, a drug-device combination of 22mg sumatriptan powder; MAP0004, a dihydroergotamine inhaler; ROX-828, ketorolac 31.5mg with 6% of lidocaine

The network diagram for the primary outcome of pain freedom at 2 h contains 17 studies (18 trials) and 11 intervention nodes (Fig. 2A). All remaining specific intranasal pharmacologic treatments, except DFN-02 10 mg and ROX-828 31.5 mg, had statistically higher ORs compared to placebo, with zolmitriptan 5 mg showing the highest odds (OR: 4.67, 95% CI: 3.43 to 6.34), followed by ketorolac 31.5 mg (OR: 3.81, 95% CI: 1.67 to 8.70), and zavegepant 10 mg showing the lowest (OR: 1.68, 95% CI: 1.18 to 2.41) (Table 1; Fig. 3A).

**Table 1:** League table of pain freedom at 2 h

**DFN-02**											2.67 (0.98,7.30)
0.70 (0.19,2.60)	**Ket**				1.19 (0.50,2.86)						**3.34 (1.25,8.87)**
0.88 (0.27,2.91)	1.25 (0.44,3.54)	**AVP-825**									**3.03(1.63,5.62)**
1.35 (0.31,5.88)	1.91 (0.49,7.40)	1.52 (0.44,5.28)	**ROX-828**								1.99 (0.66,5.83)
1.41 (0.45,4.36)	2.00 (0.83,4.83)	1.59 (0.71,3.56)	1.04 (0.32,3.41)	**Suma10mg**	**0.52 (0.34,0.79)**						**1.82 (1.19,3.14)**
0.77 (0.26,2.32)	1.10 (0.49,2.45)	0.87 (0.41,1.87)	0.57 (0.18,1.81)	**0.55 (0.36,0.83)**	**Suma20mg**						**3.48 (2.26,5.33)**
0.86 (0.28,2.67)	1.23 (0.47,3.22)	0.98 (0.44,2.17)	0.64 (0.20,2.09)	0.61 (0.30,1.24)	1.12 (0.58,2.16)	**Zol2.5 mg**	*0.59 (0.37,0.94)				**3.06 (1.74,5.59)**
0.57 (0.20,1.65)	0.82 (0.34,1.97)	0.65 (0.32,1.30)	0.43 (0.14,1.30)	**0.41 (0.23,0.74)**	0.74 (0.44,1.26)	^a^0.66 (0.42,1.05)	**Zol5mg**				**4.66 (3.43,6.35)**
1.59 (0.54,4.65)	2.26 (0.92,5.56)	1.80 (0.87,3.71)	1.18 (0.38,3.65)	1.13 (0.61,2.09)	**2.06 (1.18,3.60)**	**1.84 (1.01,3.40)**	**2.77 (1.73,4.44)**	**Zave**			**1.68 (1.18,2.41)**
0.73 (0.23,2.29)	1.03 (0.39,2.77)	0.82 (0.36,1.88)	0.54 (0.16,1.79)	^b^0.52 (0.25,1.08)	0.94 (0.47,1.87)	0.84 (0.41,1.74)	1.27 (0.69,2.34)	**0.46 (0.24,0.87)**	**MAP0004**		**3.67 (2.18,3.20)**
0.73 (0.17,3.22)	1.04 (0.27,4.06)	0.83 (0.24,2.90)	0.54 (0.12,2.49)	0.52 (0.16,1.72)	0.95 (0.29,3.04)	0.84 (0.26,2.79)	1.27 (0.41,3.93)	0.46 (0.15,1.44)	1.00 (0.30,3.37)	**Buto**	**3.67(1.25,10.7)**
^b^2.68 (0.97,7.38)	**3.81 (1.67,8.70)**	**3.03 (1.62,5.69)**	1.99 (0.68,5.81)	**1.91 (1.15,3.15)**	**3.48 (2.27,5.33)**	**3.10 (1.89,5.10)**	**4.67 (3.43,6.34)**	**1.68 (1.18,2.41)**	**3.69 (2.15,6.31)**	**3.67 (1.24,10.87)**	**Placebo**

Pairwise (upper-right portion) and network (lower-left portion) meta-analysis results are presented as estimated effect sizes for pain freedom at 2 h. For the result, outcomes are expressed as odds ratios (OR) with 95% credible interval (CI) (OR of > 1 indicated that the treatment specified in the row got more improvement than that specified in the column), 0 < OR < 1, the opposite. For the network meta-analysis, OR of > 1 indicated that the treatment specified in the column got better improvement than that specified in the row, 0 < OR < 1, the opposite. 95% CI that did not contain one was considered to have a statistical difference

Bold results indicated statistical significance. Marked with ^a^indicated a significant difference between direct and mixed comparisons. ^b^indicated a significant difference between the basic model and sensitivity analysis

DFN-02, sumatriptan nose spray 10 mg with a permeation enhancer; Ket, ketorolac nose spray 31.5 mg; Suma10mg, sumatriptan nose spray 10 mg; Suma20mg, sumatriptan nose spray 20 mg; Zol2.5 mg, zolmitriptan nose spray 2.5 mg; Zol5mg, zolmitriptan nose spray 5 mg; Zave, zavegepant nose spray 10 mg; Buto, butorphanol nose spray 1 mg; AVP-825, a drug-device combination of 22 mg sumatriptan powder; MAP0004, a dihydroergotamine inhaler; ROX-828, ketorolac 31.5 mg with 6% of lidocaine; DHE, dihydroergotamine nose spray 2 mg

In the base model, sumatriptan 20 mg showed better efficacy than sumatriptan 10 mg in both direct (OR: 0.52, 95% CI: 0.34 to 0.79) and mixed comparisons (OR: 0.55, 95% CI: 0.36 to 0.83); zolmitriptan 5 mg presented higher OR than sumatriptan 10 mg (OR: 0.41, 95% CI: 0.23 to 0.74); zavegepant 10 mg showed lower OR than sumatriptan 20 mg (OR: 2.06, 95% CI: 1.18 to 3.60), zolmitriptan 2.5 mg (OR: 1.84, 95% CI: 1.01 to 3.40), zolmitriptan 5 mg (OR: 2.77, 95% CI: 1.73 to 4.44) and MAP0004 1 mg (OR: 0.46, 95% CI: 0.24 to 0.87). Although inconsistent results existed for zolmitriptan 5 mg versus zolmitriptan 2.5 mg in pairwise meta-analyses and network meta-analyses, there was no significant difference. For the remaining outcomes, the results were mostly homogeneous. According to SUCRA (eTable 2), zolmitriptan 5 mg was ranked as the best treatment for pain freedom at 2 h, followed by ketorolac 31.5 mg and MAP0004 1 mg.

The results of sensitivity analysis 1 (lower-left portion) in eTable 18, excluding 4 studies with a high risk of bias to check the reliability of the combined effect sizes, were consistent with those of the base model. In the base model, DFN-02 was associated with a higher OR (2.68) versus placebo, but the 95% CI contained the null effect (0.97 to 7.38). However, in sensitivity analyses 2 (the upper-right portion in eTable 2) and 3 (eTable 19), although the OR did not change markedly (2.68 and 2.67), the CI became narrower, thus being statistically significant (1.05 to 6.83). The same trend was also seen in the comparison between MAP0004 1 mg versus sumatriptan 10 mg. These differences between MAP0004 1 mg versus sumatriptan 10 mg did not reach significance in the basic model, but in sensitivity analysis 3, the difference between MAP0004 and sumatriptan 10 mg became statistically significant (OR: 0.54, 95% CI: 0.30 to 0.97).

#### Secondary efficacy outcome: pain freedom at 1 h

The network diagram is shown in Fig. 2G. In brief, the network diagram shows 7 trials and 5 intervention nodes. Butorphanol 1 mg, zolmitriptan 5 mg, and MAP0004 1 mg were associated with significantly higher ORs versus placebo, with butorphanol 1 mg showing the highest OR (8.62, 95% CI: 1.11–66.92) (Fig. 3G), but with a broad CI and low quality of evidence. ROX-828 and sumatriptan 20 mg had higher ORs relative to the placebo, but the CI was insignificant. Indirect comparisons between active drugs did not show significant differences (eTable 13). Direct comparison and mixed comparisons were consistent. According to SUCRA (eTable 8), 1 mg of butorphanol was associated with the best treatment for pain freedom at 1 h, followed by 20 mg of sumatriptan.

#### Secondary efficacy outcome: sustained pain freedom for 24 h

A total of 10 studies and 9 treatment nodes were included for the outcome (Fig. 2F). All pharmacologic treatments showed significantly higher odds of improvement versus placebo when data were combined in the NMA, except for sumatriptan 20 mg with a null CI (0.64 to 6.64) (eTable 14 and Fig. 3F). Zolmitriptan 5 mg was associated with the highest OR (5.49, 95% CI: 3.58 to 8.42), followed by DFN-02 (OR: 4.59, 95% CI: 1.41–14.96), and zavegepant 10 mg showed the lowest OR (OR: 1.74, 95% CI: 1.15 to 2.64). zolmitriptan 5 mg showed better efficacy than zavegepant 10 mg (OR: 3.15, 95% CI: 1.73–5.72). Direct comparison and mixed comparisons were consistent. According to the SUCRA (eTable 7), zolmitriptan 5 mg was ranked as the best treatment for sustained pain freedom to 24 h, with a SUCRA value of 85.6.

#### Secondary efficacy outcome: freedom from nausea at 2 h

For freedom from nausea at 2 h, the main result revealed that most interventions yielded significantly higher ORs than placebo (no statistical significance in ROX-828 and zavegepant 10 mg), as shown in Fig. 3C and eTable 15. DFN-02 was associated with the highest OR (4.95, 95% CI: 1.29 to 19.01), and zavegepant 10 mg showed the lowest OR (OR: 1.08, 95% CI: 0.87 to 1.35). In an indirect comparison among active drugs, DFN-02, sumatriptan 10, 20 mg, and zolmitriptan 2.5, 5 mg were estimated to be significantly superior to zavegepant 10 mg. Sumatriptan 20 mg and zolmitriptan 5 mg showed higher efficacy than MAP0004 1 mg. Direct and mixed comparisons were generally consistent, except for ketorolac 31.5 mg versus placebo, which was not meaningful in the direct comparison. However, the OR increased and grew significantly after mixing the indirect comparisons. According to the SUCRA results, DFN-02 was ranked as the best treatment among all the interventions (eTable 4), with a SUCRA value of 87.

#### Secondary efficacy outcome: freedom from photophobia at 2 h

All pharmacologic treatments demonstrated significantly higher odds of inducing freedom from photophobia at 2 h versus placebo (Fig. 3D), with ROX-828 showing the highest improvement (OR: 4.03, 95% CI: 1.66 to 9.81), followed by DFN-02, which showed similar efficacy (OR: 4.00, 95% CI: 1.48 to 10.83], and zavegepant 10 mg showing the lowest (OR: 1.38, 95% CI: 1.06 to 1.79) among all interventions. Zavegepant 10 mg was associated with lower odds of achieving photophobia freedom at 2 h compared with all treatments (no statistical significance compared to ketorolac 31.5 mg and sumatriptan 10 mg). Direct and mixed comparisons were generally consistent (eTable 16). According to SUCRA (eTable 5), ROX-828 was the best treatment for freedom from photophobia at 2 h, followed by DFN-02 and zolmitriptan 5 mg.

#### Secondary efficacy outcome: freedom from phonophobia at 2 h

Freedom from phonophobia at 2 h was achieved for all treatments compared to placebo, except for ROX-828 (Fig. 3E). DEN-02 presented the highest (OR: 5.36, 95% CI: 1.67 to 17.22), followed by ketorolac 31.5 mg (OR: 3.36, 95% CI: 1.30 to 8.66) and ROX-828 (OR: 1.02, 95% CI: 0.36 to 2.83). Zolmitriptan 5 mg was estimated to be significantly superior to sumatriptan 10 mg, zavegepant 10 mg, and ROX-828; sumatriptan 20 mg yielded a significantly better OR than the 10 mg dose of sumatriptan. Direct and mixed comparisons revealed no significant difference (eTable 17). According to SUCRA (eTable 6), DFN-02 was associated with the best treatment, followed by zolmitriptan 5 mg and ketorolac 31.5 mg.

### Safety analysis

Regarding AEs, 17 studies (18 trials) and 11 treatments are included in Figs. 2B and 3B. Based on our NMA, the odds of AEs were significantly higher with treatments than placebo (with no statistical significance in DFN-02, MAP0004, and ROX-828), particularly dihydroergotamine 2 mg, which showed the largest OR (9.65, 95% CI: 4.39 to 21.22) for AEs, followed by ketorolac 31.5 mg (OR: 9.08, 95% CI: 3.46 to 23.86); triptans were associated with a higher OR for AEs with a trend of a dose-response relationship, including sumatriptan 10, 20 mg and zolmitriptan 2.5, 5 mg. In other words, AEs were less likely to occur with zavegepant 10 mg than with placebo (OR: 2.04, 95% CI: 1.37 to 3.03) and were almost equally possible with zolmitriptan 2.5 mg (OR: 2.04, 95% CI: 1.18 to 3.54). ROX-828 and MAP0004 1 mg had lower ORs than placebo but were not statistically significant.

The mixed comparison (Table 2) of AEs showed that sumatriptan 20 mg was estimated to have a significantly higher OR than sumatriptan 10 mg (OR: 0.65, 95% CI: 0.42 to 0.92), but zolmitriptan 5 mg was not significantly higher than zolmitriptan 2.5 mg (OR: 0.61, 95% CI: 0.35 to 1.04). Dihydroergotamine 2 mg was associated with a higher OR for AEs than most of the other treatments. MAP0004, a new orally inhaled delivery device of dihydroergotamine, showed lower AEs (OR: 6.92, 95% CI: 2.67 to 17.91) and higher safety compared to dihydroergotamine. The results of the direct and mixed comparisons were generally consistent, except that ketorolac 31.5 mg (OR: 16.93, 95% CI: 5.71 to 50.24) had a significantly higher OR and CI in the direct comparison. According to SUCRA (eTable 3), apart from the placebo, ROX-828 had the highest probability of rating first in reducing AEs (SUCRA 85.9), followed by MAP0004 1 mg (SUCRA 81.9); dihydroergotamine 2 mg ranked the lowest.

**Table 2:** League table of of adverse events

**DFN-02**											10.52 (0.54,203.3)
1.16 (0.05,26.10)	**Ket**				1.35 (0.45,4.00)						^a^16.93 (5.71,50.24)
1.51 (0.07,32.30)	1.30 (0.37,4.56)	**AVP-825**									**6.98 (3.14,15.54)**
8.71 (0.40,187.8)	**7.52 (2.13,26.57)**	**5.78 (1.85,18.09)**	**ROX-828**								1.21 (0.54,2.73)
4.48 (0.23,88.54)	**3.86 (1.42,10.53)**	^b^2.97 (1.23,7.18)	0.51 (0.21,1.26)	**Suma10mg**	**0.69 (0.49,0.99)**						**2.15 (1.47,3.16)**
2.91 (0.15,57.39)	2.51 (0.96,6.60)	1.93 (0.81,4.61)	**0.33 (0.14,0.81)**	^**b**^**0.65 (0.46,0.92)**	**Suma20mg**						**3.61 (2.58,5.09)**
5.15 (0.25,104.7)	**4.45 (1.46,13.50)**	**3.42 (1.30,9.02)**	0.59 (0.22,1.58)	1.15 (0.59,2.23)	1.77 (0.93,3.37)	**Zol2.5 mg**	0.63 (0.35,1.14)				**1.96 (1.05,3.62)**
3.11 (0.16,61.21)	2.69 (0.97,7.43)	2.07 (0.88,4.89)	**0.36 (0.15,0.85)**	0.70 (0.43,1.13)	1.07 (0.67,1.70)	0.61 (0.35,1.04)	**Zol5mg**				**3.38 (2.46,4.63)**
5.16 (0.26,102.5)	**4.46 (1.57,12.66)**	**3.43 (1.40,8.37)**	0.59 (0.24,1.47)	1.15 (0.70,1.99)	**1.77 (1.05,2.99)**	1.00 (0.51,1.98)	1.66 (0.99,2.75)	**Zave**			**2.04 (1.37,3.03)**
1.09 (0.05,23.35)	0.94 (0.27,3.27)	0.72 (0.23,2.22)	**0.13 (0.04,0.39)**	**0.24 (0.10,0.58)**	**0.37 (0.16,0.88)**	**0.21 (0.08,0.55)**	**0.35 (0.15,0.82)**	**0.21 (0.09,0.51)**	**DHE2mg**		**9.65 (4.39,21.22)**
7.54 (0.37,152.8)	**6.51 (2.16,19.62)**	**5.01 (1.91,13.09)**	0.87 (0.33,2.29)	1.68 (0.88,3.23)	**2.59 (1.38,4.88)**	1.46 (0.68,3.15)	**2.42 (1.30,4.50)**	1.46 (0.75,2.84)	**6.92 (2.67,17.91)**	**MAP0004**	1.40 (0.82,2.38)
10.51 (0.54,203.32)	^**a**^**9.08 (3.46,23.86)**	6.98 (3.14,15.54)	1.21 (0.54,2.73)	**2.35 (1.62,3.41)**	**3.61 (2.57,5.07)**	**2.04 (1.18,3.54)**	**3.38 (2.46,4.63)**	**2.04 (1.37,3.03)**	**9.65 (4.39,21.22)**	1.40 (0.82,2.38)	**Placebo**

Pairwise (upper-right portion) and network (lower-left portion) meta-analysis results are presented as estimated effect sizes for the adverse events. For the pairwise meta-analyses, outcomes are expressed as odds ratios (OR) with 95% credible interval (CI) (OR of > 1 indicated that the treatment specified in the row got more improvement than that specified in the column),0 < OR < 1, the opposite. For the network meta-analysis, OR of > 1 indicated that the treatment specified in the column got better improvement than that specified in the row, 0 < OR < 1, the opposite.95% CI that did not contain one was considered to have a statistical difference

Bold results indicated statistical significance. ^a^indicated a significant difference between direct and mixed comparisons. ^b^indicated a significant difference between the basic model and sensitivity analysis

DFN-02, sumatriptan nose spray 10 mg with a permeation enhancer; Ket, ketorolac nose spray 31.5 mg; Suma10mg, sumatriptan nose spray 10 mg; Suma20mg, sumatriptan nose spray 20 mg; Zol2.5 mg, zolmitriptan nose spray 2.5 mg; Zol5mg, zolmitriptan nose spray 5 mg; Zave, zavegepant nose spray 10 mg; Buto, butorphanol nose spray 1 mg; AVP-825, a drug-device combination of 22 mg sumatriptan powder; MAP0004, a dihydroergotamine inhaler; ROX-828, ketorolac 31.5 mg with 6% of lidocaine; DHE, dihydroergotamine nose spray 2 mg

Most results for sensitivity analyses (eTables 20 to 21) were generally consistent with the base case analysis. Exceptions included that the odds of AEs decreased for AVP-825 versus sumatriptan 10 mg and became meaningless in sensitivity analysis 1. Sumatriptan 10 mg improved the odds of AEs versus sumatriptan 20 mg, but these differences did not reach significance in sensitivity analyses 1 and 3.

For most studies, AEs were mild to moderate. Dysgeusia was the most commonly reported AE for triptans and ROX-828. Burning of the nose was linked to the most commonly occurring AEs for 31.5 mg ketorolac. The most frequent AE for dihydroergotamine 2 mg was rhinitis (primarily nasal congestion), while for butorphanol 1 mg, the most frequent complication was dizziness (eTable 12).

There were insufficient data to perform a quantitative comparison of SAEs, treatment-related SAEs, and study withdrawal due to treatment related SAEs. This is because the included studies either did not report data in all intervention arms or had zero events. Thus, we characterized the available data only. The proportion of SAEs was small and mostly unrelated to treatments. Three patients treated with zolmitriptan nasal spray 2.5 mg in Charlesworth et al.’s 2003 study [61] reported SAEs. However, only one case of potential cardiac origin (chest pain) after taking the study medication was considered medication related. In the three studies [59–61] examining zolmitriptan nasal spray 5 mg, six patients reported SAEs, although these were not thought to be related to the study drug. For ROX-828 and zavegepant 10 mg, SAEs were reported in two and one patient, respectively, but were not considered drug-related. One patient treated with 2 mg of dihydroergotamine nasal spray [65] experienced an AE that was considered drug related. This patient presented with moderate nasal swelling beginning 15 min after treatment and lasting 2 h, followed by moderate peripheral oedema beginning 7.5 h after dosing and lasting 12 h.

Sixteen participants on zolmitriptan nasal spray 5 mg withdrew because of AEs related to the study drug, while six patients withdrew in the placebo group. One patient in the dihydroergotamine nasal spray 2 mg group withdrew due to persistent nasal swelling. One study participant in the MAP0004 1 mg group in Aurora et al.’s 2009 study [57] discontinued because of AEs. No other subjects in the remaining studies withdrew after taking the study medication or were not reported.

### Publication bias and inconsistency

In the risk of bias assessment, 52.7% (10/19 studies), 26.3% (5/19 studies), and 21.0% (4/19 studies) had an overall low risk of bias, some concerns, and high risk of bias, respectively (eFigures 1 and 2). The randomization process and measurement of the outcome led to high and unclear risks of bias, respectively. The funnel plot of publication bias across the included studies showed general levels of symmetry (eFigure 3), and the results of the Egger tests indicated no significant publication bias among the base NMA analysis. There was no triangular loop formation for pain freedom at 2 and 24 h; hence, there was no potential source of inconsistency. There was no significant global inconsistency in the design-by-treatment model (eTable 9). For local inconsistency (eTable 10), there was one triangular loop in loop-specific inconsistencies regarding pain freedom at 2 h (zolmitriptan 2.5 mg-zolmitriptan 5 mg-placebo). Side-splitting inconsistencies were also exhibited but were few overall (eTable 11). The GRADE results assessment is presented in eAppendix 3. Overall, the quality of evidence for most comparisons in the current NMA ranged from low to very low.

## Discussion

To date, few RCTs have directly compared active intranasal drugs for the treatment of acute migraine, mostly involving placebo controls. We conducted an NMA, combining direct and indirect comparisons and then ranking the interventions’ efficacy and safety to yield more accurate comparisons than RCTs and traditional meta-analyses. According to our search, this is the first pilot NMA comparing intranasal drugs of currently specific acute migraine medications. For acute migraine attacks that include severe nausea or vomiting that cause difficulties in taking oral forms, a slow onset of action, or poor tolerability [13], clinicians can make relevant comparisons of available intranasal medications by referring to our findings.

In general, our study findings suggest that all interventions demonstrated superiority over the placebo regarding pain freedom at 2 h, although there were no statistically significant differences in DFN-02 and ROX-828. Zolmitriptan 5 mg was associated with the best therapeutic efficacy compared to the other investigated interventions in terms of pain freedom at 2 h and sustained pain freedom for 24 h. Butorphanol 1 mg was associated with the highest effectiveness in pain freedom at 1 h but with broad CI and low GRADE levels. DFN-02 ranked first in terms of freedom from nausea at 2 h and freedom from phonophobia at 2 h; ROX-828 was associated with the highest efficacy of freedom from photophobia at 2 h among all treatments, yet they were each based on one study with a wide CI and nonsignificant differences given the variance within the studies. Regarding safety and tolerability, the lowest OR and narrower CI indicate a more favourable risk profile and greater precision around the estimate of zavegepant nasal spray 10 mg compared with other investigated interventions. Dihydroergotamine 2 mg was associated with the highest risk of AEs among all treatments. Overall, the results of the base case analyses for the primary endpoints (pain freedom at 2 h and AEs) were supported by those of the three sensitivity analyses.

With conventional liquid nasal spray pumps for delivery, intranasal liquid spray formulations of sumatriptan [34] and zolmitriptan [35] provided significant migraine freedom versus placebo. Zolmitriptan 5 mg achieved the best efficacy in terms of pain freedom at 2 h of the primary outcome but was associated with a higher OR of AEs, with a SUCRA of 23.5. The efficacy of zolmitriptan showed a direct correlation with its dosage. Zolmitriptan 5 mg had a significantly higher OR than zolmitriptan 2.5 mg in all efficacy outcomes, but AEs became more frequent with increasing dosage. Sumatriptan nasal spray also presented similar dose-dependent trends.

DFN-02, comprising sumatriptan 10 mg plus a permeation-enhancing excipient, was developed to overcome the low bioavailability (~ 17%), slow absorption (t_max_ = 0.88 to 1.75 h), and slow onset of action (30 to 45 min) of conventional sumatriptan intranasal delivery [68, 69]. It had a faster absorption profile (single-dose t_max_ = 15 min) than sumatriptan nasal spray in healthy subjects, and systemic exposure from a single-dose DFN-02 was similar to 4 mg subcutaneous sumatriptan [69]. Our study shows that DFN-02 achieved better efficacy than sumatriptan 10 mg for pain freedom at 2 h and was even more effective than sumatriptan 20 mg in providing sustained pain freedom at 24 h. However, the CI contained the null effect. DFN-02 ranked the highest effectiveness in freedom from nausea and phonophobia at 2 h and achieved significant efficacy in freedom from photophobia, which may be prioritized for acute migraine attacks with significant bothersome symptoms. For safety and tolerability, DFN-02 was associated with the highest risk of any AEs (wide CI and null effect). No subjects discontinued the study medication due to AEs. The above results indicated that DFN-02 was superior to conventional same-dose sumatriptan nasal spray for pain freedom and relief from bothersome symptoms, but the AEs also increased. Considering that DFN-02 was a small-sample (total sample size of 93 patients) crossover study evaluated and low-GRADE evidence by one trial, such results seem to lack sufficient stability, and the ongoing phase 3 trial may provide further evidence for DFN-02.

Conventional intranasal treatments employ standard nasal spray pumps [70, 71]. A substantial portion of the dose is deposited in the anterior nasal cavity, which may drain out of the nose or be swallowed. Only a limited portion penetrates beyond the nasal valve to the vascular mucosa in the most posterior regions of the nasal cavity [17, 72, 73]. Unlike conventional liquid nasal spray devices, AVP-825 delivers a powder utilizing a new unique breath-powered intranasal delivery system that allows the drug to reach the rich vascular mucosa of the posterior nasal cavity [17, 53], which favours rapid absorption of the drug. The results of the PK study showed that AVP-825 delivered sumatriptan more efficiently, with higher and earlier peak exposure compared to conventional liquid nasal spray within the first 30 min post-dose, and faster absorption than oral tablets or subcutaneous injections over the first 15 min post-dose [74]. Pooled results from placebo-controlled phase 2 and 3 studies showed significantly higher percentages of patients in freedom with AVP compared to placebo at all time points from 30 to 120 min post-dose [53, 56, 75]. In the present NMA, AVP-825 was superior to sumatriptan nasal spray 10 mg but inferior to 20 mg of the same drug. However, none of these comparisons revealed statistically significant differences. Regarding safety, AVP-825 was associated with a higher risk for AEs than sumatriptan nasal spray 20 mg, which was inconsistent with the results described by the authors [17], even though the AEs were tolerable and were not considered severe. The fact that few AEs data were available for AVP-825 (only 54) might cause bias to some extent.

Two ergot derivatives were included in the current NMA: dihydroergotamine nasal spray 2 mg and MAP0004 1 mg, an orally inhaled version of dihydroergotamine. Dihydroergotamine nasal spray was associated with the highest risk of AEs among all treatments. MAP0004 demonstrated superior efficacy and safety; however, MAP0004 failed to overcome manufacturing problems and thus never gained approval from the Food and Drug Administration (FDA). Although ergometrine derivatives have some poor tolerability (nausea, vomiting, and cardiovascular effects), it has a rapid onset of action, sustained effect, and low risk of drug overuse headache [76], which is effective in acute migraine, even in patients with difficult-to-treat migraines, such as patients with abnormal pain or frequent recurrences [77], or when administered in the late phase of an attack [78]. Dihydroergotamine represents a promising clinical prospect, with at least three different new intranasal delivery dihydroergotamine products (INP104, STS101, and DFN-19) in clinical development to meet the unsatisfied drug needs of migraine patients.

Ketorolac, an NSAID that acts mainly by inhibiting cyclo-oxygenase (COX) 1 and 2, was approved by the FDA for moderate to severe pain in an oral, intravenous, or nasal spray formulation [79]. Intranasal ketorolac had a better analgesic effect than placebo, with statistical significance among adult patients with acute postoperative pain in well-designed RCTs [80–82]. In the present study, 31.5 mg ketorolac nasal spray showed the second-best efficacy results for pain freedom at 2 h and deserves priority recommendation; however, it was associated with a higher risk of AEs. The AEs were tolerable and were not considered severe. Ketorolac 31.5 mg [52] was only evaluated in one trial, and the total sample size was only 54 patients from the first stage of a crossover trial. The KSPN inclusion criteria [52] excluded patients on opioids within two months and may have limited the inclusion of some patients with treatment resistance, resulting in higher effect values. Because of the moderate GRADE level, we recommend weighing the benefit against the risk of AEs when selecting ketorolac nasal spray.

ROX-828, composed of 31.5 mg ketorolac with 6% lidocaine, was evaluated for efficacy and safety in the acute treatment of migraine. Compared to intranasal ketorolac without lidocaine, lidocaine significantly decreases the time to t_max_ without affecting the c_max_ and improves tolerability [54]. A meta-analysis [83] revealed that participants who received intranasal lidocaine had lower pain intensity, a higher success rate, and less frequent need for rescue medicine than the control group. Despite being inferior to ketorolac 31.5 mg for pain freedom at 2 h, ROX-828 was associated with a higher OR than placebo and the lowest risk of AEs among all treatments. ROX-828 showed superiority in freedom from photophobia at 2 h, which may be worth considering for migraine patients experiencing severe photophobia.

Small-molecule calcitonin gene-related peptide (CGRP) receptor antagonists (gepants) [84–86] are emerging and recently approved acute antimigraine therapies and have a potentially more favourable acute cardiovascular adverse effect profile than triptans. Zavegepant nasal spray [49, 50], the first CGRP receptor antagonist for intranasal administration for the symptomatic treatment of patients suffering from acute migraine attacks, was approved by the FDA on 9 March 2023. At the time of our research, the results of the phase 3 trial [49] had been reported and included in the current NMA. The emergence of zavegepant nasal spray has enriched the management available for acute migraine; however, compared with zavegepant nasal spray, most treatments (e.g., all doses of triptans, MAP0004, and ketorolac) included in the current NMA had higher ORs for all efficacy outcomes after the dose, which may imply that triptans will remain the current mainstay of acute migraine intranasal drugs. This result was consistent with the findings of a previous meta-analysis involving the comparison of oral gepants with oral triptans [86]. Although zavegepant nasal spray may be less effective than most investigated interventions, it was associated with the fewest AEs, demonstrating that this novel abortive agent has a better safety and tolerability profile. Its lack of vasoconstrictor activity provides a safer alternative therapy for patients currently at risk of cardiovascular disease.

Butorphanol is a potent opioid analgesic. Although butorphanol nasal spray 1 mg showed some limited efficacy in the present NMA, data for this drug came from an early published article that was included, and the certainty of the evidence was only at a low level. Guidelines strongly discourage the routine use of butorphanol in the acute treatment of migraine [3, 13] due to the high risks of habituation, addiction, tolerance, withdrawal syndromes, adverse effects, and medication overuse headache.

Given the favourable efficacy and safety profile of intranasal zolmitriptan, it appears promising to improve zolmitriptan using the novel delivery device (similar to AVP-825) or with a permeation-enhancing excipient (similar to DFN-02), which could fulfil the current unmet therapeutic need in migraine.

### Strengths and limitations

Our analysis results should be approached with caution given some limitations. First, the results of the NMA depend significantly on the quality and heterogeneity of the included studies. Only approximately half of the studies were of high quality (10/19), and the substantial number of moderate- (5/19) and high-risk (4/19) trials would undoubtedly diminish the results of our study. Hence, we performed sensitivity analyses by excluding high-risk bias literature and obtained generally consistent results. Another limitation of our study is that we included a small number of studies and sample sizes for some interventions, which may lead to instability, especially when using random effects models. Third, with the exception of a few studies [52, 59, 61], most of the included studies reported outcomes for a single migraine attack, and the consistency of the effects of repeated medication use is still being determined. Fourth, although we used strict inclusion and exclusion criteria to improve the homogeneity of the included studies, some potential heterogeneity between studies with respect to participant characteristics will inevitably remain (e.g., baseline age differences, the proportion of headache severity, combined medications, underlying diseases, the presence or absence of migraine aura and the concomitant use of preventive medications). We performed sensitivity analyses to exclude studies prohibiting the use of prophylactic drugs and obtained similar results. Finally, some data were extracted from the published literature. When the results are reported as percentages, we estimate the absolute values from the percentages, which may result in minor variations in the extracted values from the true values.

## Conclusions

For pain freedom at 2 h and sustained pain freedom for 24 h after the administered dose, zolmitriptan 5 mg still demonstrated the most effective therapeutic effects when compared to zavegepant and other intranasal drugs with novel techniques and delivery devices. DFN-02 presented good efficacy for freedom from nausea and phonophobia at 2 h, albeit with lower odds of achieving complete pain freedom and the highest odds of AEs. ROX-828 was associated with the highest efficacy of freedom from photophobia at 2 h. Zavegepant nasal spray 10 mg was associated with the fewest adverse side effects among all interventions. Although zavegepant nasal spray may be less effective in terms of pain freedom compared with most investigated interventions, the better safety profile and lack of vasoconstrictor activity can provide a safer alternative option for individuals for whom currently available acute treatments have failed or for those with cardiovascular contraindications.

### Supplementary Information

**Additional file 1: eAppendix 1. **PRISMA checklist of the current network meta-analysis. **eAppendix 2.** Search strategies. **eAppendix 3.** GRADE ratings for each network. **eTable 1.** Baseline demographics characteristics. **eTable 2. **SUCRA of pain -freedom at 2 hours. **eTable 3. **SUCRA of adverse events. **eTable 4. **SUCRA of freedom from nausea at 2 hours. **eTable 5. **SUCRA of freedom from photophobia at 2 hours. **eTable 6. **SUCRA of freedom from phonophobia at 2 hours. **eTable 7. **SUCRA of sustained pain -freedom for 24 hours. **eTable 8. **SUCRA of pain -freedom at 1 hour. **eTable9. **Design-by-treatment interaction model for inconsistency of network meta-analysis. **eTable 10. **Significant loop-specific inconsistencies of network meta-analysis. **eTable 11. **Significant side-splitting inconsistencies of network meta-analysis. **eTable 12. **Proportion of serious adverse events and the most commonly reported adverse events. **eTable 13. **League table of pain -freedom after 1 hour. **eTable 14. **League table ofHead-to-head comparisons of sustained pain -freedom for 24 hours. **eTable 15. **League table ofHead-to-head comparisons of freedom from nausea at 2 hours. **eTable 16. **League table of freedomHead-to-head comparisons of freedom from from photophobia at 2 hours. **eTable 17. **League table of freedom fromHead-to-head comparisons of freedom from phonophobia after 2 hours. **eTable 18. **Sensitivity analysis 1 and 2 of pain -freedom at 2 hours. **eTable 19. **Sensitivity analysis 3 of pain -freedom at 2 hours. **eTable 20. **Sensitivity analysis 1 and 2 of adverse events. **eTable 21. **Sensitivity analysis 3 of adverse events. **eFigure 1. **Overview of risk of bias. **eFigure 2. **Detailed risk of bias in each study. **eFigure 3. **Funnel plot and Egger-value results of all studies included for all endpoints. 

**Additional file 2.**

## Data Availability

The raw data supporting the conclusions of this article will be made available by the authors without undue reservation.

## Abbreviations

AEs: Adverse events
CGRP: Calcitonin gene-related peptide
CINeMA: Confidence in Network Meta-Analysis
COX: Cyclo-oxygenase
CI: Credible interval
ES: Effect sizes
FDA: Food and Drug Administration
ICHD: International Classification of Headache Disorders criteria
MBS: Most bothersome symptom
NMA: Network meta-analysis
NSAIDs: Non-steroidal anti-inflammatory drugs
OR: Odds ratio
PRISMA: Preferred Reporting Items for Systematic Reviews and Meta-Analyses
RCTs: Randomized controlled trials
SAEs: Serious adverse events
SUCRA: Surface under the cumulative ranking curve
