# Supplementary material for: Efficacy and safety of intranasal agents for the acute treatment of migraine: a systematic review and network meta-analysis
Source: J Headache Pain. 2023 Sep 18;24(1):129. doi: 10.1186/s10194-023-01662-6 (PMC10506288; doi:10.1186/s10194-023-01662-6)
Supplement: Supplementary file 1 — Additional file 1: eAppendix 1. PRISMA checklist of the current network meta-analysis. eAppendix 2. Search strategies. eAppendix 3. GRADE ratings for each network. eTable 1. Baseline demographics characteristics. eTable 2. SUCRA of pain -freedom at 2 hours. eTable 3. SUCRA of adverse events. eTable 4. SUCRA of freedom from nausea at 2 hours. eTable 5. SUCRA of freedom from photophobia at 2 hours. eTable 6. SUCRA of freedom from phonophobia at 2 hours. eTable 7. SUCRA of sustained pain -freedom for 24 hours. eTable 8. SUCRA of pain -freedom at 1 hour. eTable9. Design-by-treatment interaction model for inconsistency of network meta-analysis. eTable 10. Significant loop-specific inconsistencies of network meta-analysis. eTable 11. Significant side-splitting inconsistencies of network meta-analysis. eTable 12. Proportion of serious adverse events and the most commonly reported adverse events. eTable 13. League table of pain -freedom after 1 hour. eTable 14. League table ofHead-to-head comparisons of sustained pain -freedom for 24 hours. eTable 15. League table ofHead-to-head comparisons of freedom from nausea at 2 hours. eTable 16. League table of freedomHead-to-head comparisons of freedom from from photophobia at 2 hours. eTable 17. League table of freedom fromHead-to-head comparisons of freedom from phonophobia after 2 hours. eTable 18. Sensitivity analysis 1 and 2 of pain -freedom at 2 hours. eTable 19. Sensitivity analysis 3 of pain -freedom at 2 hours. eTable 20. Sensitivity analysis 1 and 2 of adverse events. eTable 21. Sensitivity analysis 3 of adverse events. eFigure 1. Overview of risk of bias. eFigure 2. Detailed risk of bias in each study. eFigure 3. Funnel plot and Egger-value results of all studies included for all endpoints. [file 10194_2023_1662_MOESM1_ESM.docx]

# **Supplementary material**

eAppendix 1. PRISMA checklist of the current network meta-analysis

eAppendix 2. Search strategies

eAppendix 3. GRADE ratings for each network

eTable 1. Baseline demographics characteristics

eTable 2-8. SUCRA of each outcome for anti-migraine-specific treatments

eTable 9. Design-by-treatment interaction model for inconsistency of network meta-analysis

eTable 10. Significant loop-specific inconsistencies of network meta-analysis

eTable 11. Significant side-splitting inconsistencies of network meta-analysis

eTable 12. Proportion of serious adverse events and the most commonly reported adverse events

eTable 13-17. Head-to-head comparisons of secondary outcomes

eTable 18-19. Sensitivity analysis of primary efficacy outcome of pain freedom at 2 hours

eTable 20-21. Sensitivity analysis primary safety outcome of adverse events

eFigure 1. Overview of risk of bias

eFigure 2. Detailed risk of bias in each study

eFigure 3. Funnel plot of changes in each outcome

eReferences.

This supplementary material has been provided by the authors to give readers additional information about their work.

**eAppendix 1:** PRISMA checklist of the current network meta-analysis

| **Section and Topic** | **Item #** | **Checklist item** | **Location where item is reported** |
| --- | --- | --- | --- |
| **TITLE** | | |  |
| Title | 1 | Identify the report as a systematic review incorporating a network meta-analysis (or related form of meta-analysis) | 1 |
| **ABSTRACT** | | |  |
| Abstract | 2 | See the PRISMA 2020 for Abstracts checklist. | 1 |
| **INTRODUCTION** | | |  |
| Rationale | 3 | Describe the rationale for the review in the context of existing knowledge. | 2-3 |
| Objectives | 4 | Provide an explicit statement of the objective(s) or question(s) the review addresses. | 2-3 |
| **METHODS** | | |  |
| Eligibility criteria | 5 | Specify the inclusion and exclusion criteria for the review and how studies were grouped for the syntheses. | 3-4 |
| Information sources | 6 | Specify all databases, registers, websites, organisations, reference lists and other sources searched or consulted to identify studies. Specify the date when each source was last searched or consulted. | 3 |
| Search strategy | 7 | Present the full search strategies for all databases, registers and websites, including any filters and limits used. | 3-4 |
| Selection process | 8 | Specify the methods used to decide whether a study met the inclusion criteria of the review, including how many reviewers screened each record and each report retrieved, whether they worked independently, and if applicable, details of automation tools used in the process. | 4-5 |
| Data collection process | 9 | Specify the methods used to collect data from reports, including how many reviewers collected data from each report, whether they worked independently, any processes for obtaining or confirming data from study investigators, and if applicable, details of automation tools used in the process. | 4-5 |
| Data items | 10a | List and define all outcomes for which data were sought. Specify whether all results that were compatible with each outcome domain in each study were sought (e.g. for all measures, time points, analyses), and if not, the methods used to decide which results to collect. | 4-5 |
|  | 10b | List and define all other variables for which data were sought (e.g. participant and intervention characteristics, funding sources). Describe any assumptions made about any missing or unclear information. | 5 |
| Study risk of bias assessment | 11 | Specify the methods used to assess risk of bias in the included studies, including details of the tool(s) used, how many reviewers assessed each study and whether they worked independently, and if applicable, details of automation tools used in the process. | 5 |
| Effect measures | 12 | Specify for each outcome the effect measure(s) (e.g. risk ratio, mean difference) used in the synthesis or presentation of results. | 5-6 |
| Synthesis methods | 13a | Describe the processes used to decide which studies were eligible for each synthesis (e.g. tabulating the study intervention characteristics and comparing against the planned groups for each synthesis (item #5)). | 5-6 |
|  | 13b | Describe any methods required to prepare the data for presentation or synthesis, such as handling of missing summary statistics, or data conversions. | 5-6 |
|  | 13c | Describe any methods used to tabulate or visually display results of individual studies and syntheses. | 5-6 |
|  | 13d | Describe any methods used to synthesize results and provide a rationale for the choice(s). If meta-analysis was performed, describe the model(s), method(s) to identify the presence and extent of statistical heterogeneity, and software package(s) used. | 5-6 |
|  | 13e | Describe any methods used to explore possible causes of heterogeneity among study results (e.g. subgroup analysis, meta-regression). | 5-6 |
|  | 13f | Describe any sensitivity analyses conducted to assess robustness of the synthesized results. | 5-6 |
| Reporting bias assessment | 14 | Describe any methods used to assess risk of bias due to missing results in a synthesis (arising from reporting biases). | 6 |
| Certainty assessment | 15 | Describe any methods used to assess certainty (or confidence) in the body of evidence for an outcome. | 6 |
| **RESULTS** | | |  |
| Study selection | 16 | Describe the results of the search and selection process, from the number of records identified in the search to the number of studies included in the review, ideally using a flow diagram. | 6-8  Figure 1  eAppendix2 |
| Study characteristics | 17 | Cite each included study and present its characteristics. | 6-7  eTable 1 |
| Risk of bias in studies | 18 | Present assessments of risk of bias for each included study. | eFigure 1, 2 |
| Results of individual studies | 19 | For all outcomes, present, for each study: (a) summary statistics for each group (where appropriate) and (b) an effect estimate and its precision (e.g. confidence/credible interval), ideally using structured tables or plots. | Figure 3 |
| Results of syntheses | 20a | For each synthesis, briefly summarise the characteristics and risk of bias among contributing studies. | 7-10  eFigure 1, 2 |
|  | 20b | Present results of all statistical syntheses conducted. If meta-analysis was done, present for each the summary estimate and its precision (e.g. confidence/credible interval) and measures of statistical heterogeneity. If comparing groups, describe the direction of the effect. | 7-10  Table 1 eTable13-17 |
|  | 20c | Present results of all investigations of possible causes of heterogeneity among study results. | Figure 3 |
|  | 20d | Present results of all sensitivity analyses conducted to assess the robustness of the synthesized results. | Table 18-21 |
| Reporting biases | 21 | Present assessments of risk of bias due to missing results (arising from reporting biases) for each synthesis assessed. | eFigure 1-2 |
| Certainty of evidence | 22 | Present assessments of certainty (or confidence) in the body of evidence for each outcome assessed. | eAppendix 3 |
| **DISCUSSION** | | |  |
| Discussion | 23a | Provide a general interpretation of the results in the context of other evidence. | 10-13 |
|  | 23b | Discuss any limitations of the evidence included in the review. | 14 |
|  | 23c | Discuss any limitations of the review processes used. | 14 |
|  | 23d | Discuss implications of the results for practice, policy, and future research. | 14 |
| **OTHER INFORMATION** | | |  |
| Registration and protocol | 24a | Provide registration information for the review, including register name and registration number, or state that the review was not registered. | 3 |
|  | 24b | Indicate where the review protocol can be accessed, or state that a protocol was not prepared. | not prepared |
|  | 24c | Describe and explain any amendments to information provided at registration or in the protocol. | not prepared |
| Support | 25 | Describe sources of financial or non-financial support for the review, and the role of the funders or sponsors in the review. | 16 |
| Competing interests | 26 | Declare any competing interests of review authors. | 16 |
| Availability of data, code and other materials | 27 | Report which of the following are publicly available and where they can be found: template data collection forms; data extracted from included studies; data used for all analyses; analytic code; any other materials used in the review. | 16 |

## eAppendix 2: Search strategies

**PubMed from inception until 15 August 2023**

| **#** | **Searches** | **Results** |
| --- | --- | --- |
| 1 | Migraine Disorders[Mesh] | 31,887 |
| 2 | Migraine disorders[Title/Abstract] OR Disorder, Migraine[Title/Abstract] OR Disorders, Migraine[Title/Abstract] OR Migraine Disorder[Title/Abstract] OR Migraine[Title/Abstract] OR Migraines[Title/Abstract] OR Migraine Headache[Title/Abstract] OR Headache, Migraine[Title/Abstract] OR Headaches, Migraine[Title/Abstract] OR Migraine Headaches[Title/Abstract] OR Acute Confusional Migraine[Title/Abstract] OR Acute Confusional Migraines[Title/Abstract] OR Migraine, Acute Confusional[Title/Abstract] OR Migraines, Acute Confusional[Title/Abstract] OR Status Migrainosus[Title/Abstract] OR Hemicrania Migraine[Title/Abstract] OR Hemicrania Migraines[Title/Abstract] OR Migraine, Hemicrania[Title/Abstract] OR Migraines, Hemicrania[Title/Abstract] OR Migraine Variant[Title/Abstract] OR Migraine Variants[Title/Abstract] OR Variant, Migraine[Title/Abstract] OR Variants, Migraine[Title/Abstract] OR Sick Headache[Title/Abstract] OR Headache, Sick[Title/Abstract] OR Headaches, Sick[Title/Abstract] OR Sick Headaches[Title/Abstract] OR Abdominal Migraine[Title/Abstract] OR Abdominal Migraines[Title/Abstract] OR Migraine, Abdominal[Title/Abstract] OR Migraines, Abdominal[Title/Abstract] OR Cervical Migraine Syndrome[Title/Abstract] OR Cervical Migraine Syndromes[Title/Abstract] OR Migraine Syndrome, Cervical[Title/Abstract] OR Migraine Syndromes, Cervical[Title/Abstract] | 42,974 |
| 3 | 1 OR 2 | 47,177 |
| 4 | Administration, Intranasal[Mesh] | 16,384 |
| 5 | Intranasal Drug Administration[Title/Abstract] OR Drug Administration, Intranasal[Title/Abstract] OR Intranasal Administration[Title/Abstract] OR Administrations,Intranasal[Title/Abstract] OR Intranasal Administrations[Title/Abstract] OR Administration, Intranasal Drug[Title/Abstract] OR Administrations, Intranasal Drug[Title/Abstract] OR Drug Administrations, Intranasal[Title/Abstract] OR Intranasal Drug Administrations[Title/Abstract] OR Administration, Nasal[Title/Abstract] OR Administrations, Nasal[Title/Abstract] OR Nasal Administration[Title/Abstract] OR Nasal Administrations[Title/Abstract] | 5,717 |
| 6 | 4 OR 5 | 18,791 |
| 7 | Nasal Sprays[Mesh] | 871 |
| 8 | Sprays, Nasal[Title/Abstract] OR Nasal Mist[Title/Abstract] OR Mist, Nasal[Title/Abstract] OR Nasal Aerosol[Title/Abstract] OR Aerosol, Nasal[Title/Abstract] OR Nasal Spray[Title/Abstract] OR Spray, Nasal[Title/Abstract] | 3,498 |
| 9 | 7 OR 8 | 3,756 |
| 10 | 6 OR 9 | 20,968 |
| 11 | (clinical[tiab] AND trial[tiab]) OR clinical trials as topic[mesh] OR clinical trial[pt] OR random*[tiab] OR random allocation[mesh] OR therapeutic use[sh] | 6,342,487 |
| 12 | animals[Mesh] NOT humans[Mesh] | 5,145,339 |
| 13 | 11 NOT 12 | 5,558,885 |
| 14 | 3 AND 10 AND 13 | 282 |

**Embase from inception until 15 August 2023**

| **#** | **Searches** | **Results** |
| --- | --- | --- |
| 1 | 'migraine disorders'/exp | 80,763 |
| 2 | 'migraine disorders':ab,ti,kw OR 'disorder, migraine':ab,ti,kw OR 'disorders, migraine':ab,ti,kw OR 'migraine disorder':ab,ti,kw OR 'migraine':ab,ti,kw OR 'migraines':ab,ti,kw OR 'migraine headache':ab,ti,kw OR 'headache, migraine':ab,ti,kw OR 'headaches, migraine':ab,ti,kw OR 'migraine headaches':ab,ti,kw OR 'acute confusional migraine':ab,ti,kw OR 'acute confusional migraines':ab,ti,kw OR 'migraine, acute confusional':ab,ti,kw OR 'migraines, acute confusional':ab,ti,kw OR 'status migrainosus':ab,ti,kw OR 'hemicrania migraine':ab,ti,kw OR 'hemicrania migraines':ab,ti,kw OR 'migraine, hemicrania':ab,ti,kw OR 'migraines, hemicrania':ab,ti,kw OR 'migraine variant':ab,ti,kw OR 'migraine variants':ab,ti,kw OR 'variant, migraine':ab,ti,kw OR 'variants, migraine':ab,ti,kw OR 'sick headache':ab,ti,kw OR 'headache, sick':ab,ti,kw OR 'headaches, sick':ab,ti,kw OR 'sick headaches':ab,ti,kw OR 'abdominal migraine':ab,ti,kw OR 'abdominal migraines':ab,ti,kw OR 'migraine, abdominal':ab,ti,kw OR 'migraines, abdominal':ab,ti,kw OR 'cervical migraine syndrome':ab,ti,kw OR 'cervical migraine syndromes':ab,ti,kw OR 'migraine syndrome, cervical':ab,ti,kw OR 'migraine syndromes, cervical':ab,ti,kw | 68,241 |
| 3 | 1 OR 2 | 89,028 |
| 4 | 'intranasal drug administration'/exp | 14,684 |
| 5 | 'intranasal drug administration':ab,ti,kw OR 'drug administration, intranasal':ab,ti,kw OR 'intranasal administration':ab,ti,kw OR 'administrations,intranasal':ab,ti,kw OR 'intranasal administrations':ab,ti,kw OR 'administration, intranasal drug':ab,ti,kw OR 'administrations, intranasal drug':ab,ti,kw OR 'drug administrations, intranasal':ab,ti,kw OR 'intranasal drug administrations':ab,ti,kw OR 'administration, nasal':ab,ti,kw OR 'administrations, nasal':ab,ti,kw OR 'nasal administration':ab,ti,kw OR 'nasal administrations':ab,ti,kw | 7,280 |
| 6 | 4 OR 5 | 19,574 |
| 7 | 'nose spray'/exp | 4,420 |
| 8 | Nasal sprays:ab,ti,kw OR Sprays, Nasal:ab,ti,kw OR Nasal Mist:ab,ti,kw OR Mist, Nasal:ab,ti,kw OR Nasal Aerosol:ab,ti,kw OR Aerosol, Nasal:ab,ti,kw OR Nasal Spray:ab,ti,kw OR Spray, Nasal:ab,ti,kw | 5,780 |
| 9 | 7 OR 8 | 7,414 |
| 10 | 6 OR 9 | 25,461 |
| 11 | 'clinical':ti,ab AND 'trial':ti,ab OR 'clinical trial'/exp OR random* OR 'drug therapy':lnk | 7,011,790 |
| 12 | 3 AND 10 AND 11 | 621 |

**Cochrane Library from inception until 15 August 2023**

| **#** | **Searches** | **Results** |
| --- | --- | --- |
| 1 | MeSH descriptor: [Migraine disorders] explode all trees | 3,523 |
| 2 | migraine disorders OR disorder, migraine OR disorders, migraine OR migraine disorder OR migraine OR migraines OR migraine headache OR headache, migraine OR headaches, migraine OR migraine headaches OR acute confusional migraine OR acute confusional migraines OR migraine, acute confusional OR migraines, acute confusional OR status migrainosus OR hemicrania migraine OR hemicrania migraines OR migraine, hemicrania OR migraines, hemicrania OR migraine variant OR migraine variants OR variant, migraine OR variants, migraine OR sick headache OR headache, sick OR headaches, sick OR sick headaches OR abdominal migraine OR abdominal migraines OR migraine, abdominal OR migraines, abdominal OR cervical migraine syndrome OR cervical migraine syndromes OR migraine syndrome, cervical OR migraine syndromes, cervical | 10,051 |
| 3 | 1 OR 2 | 10,051 |
| 4 | Administration,Intranasal OR Intranasal Drug Administration OR Drug Administration, Intranasal OR Intranasal Administration OR Administrations,Intranasal OR Intranasal Administrations OR Administration, Intranasal Drug OR Administrations, Intranasal Drug OR Drug Administrations, Intranasal OR Intranasal Drug Administrations OR Administration, Nasal OR Administrations, Nasal OR Nasal Administration OR Nasal Administrations | 10,582 |
| 5 | MeSH descriptor: [Nasal sprays] explode all trees | 325 |
| 6 | Nasal sprays OR Sprays, Nasal OR Nasal Mist OR Mist, Nasal OR Nasal Aerosol OR Aerosol, Nasal OR Nasal Spray OR Spray, Nasal | 4,817 |
| 7 | 5 OR 6 | 4,817 |
| 8 | 4 OR 7 | 12,898 |
| 9 | 3 AND 8 | 322 |
| 10 | In trials | 264 |

eAppendix 3: GRADE ratings for each network

The Grading of Recommendations Assessment, Development, and Evaluation (GRADE) methods provide confidence assessments of the results of systematic reviews and meta-analyses and have been widely accepted and applied [1, 2]. However, the complexity of the methods and the lack of suitable software have limited their adoption. Confidence in Network Meta-Analysis (CINeMA) [3] is an approach for determining confidence in the results of an NMA broadly based on GRADE, with several conceptual and semantic differences. It covers 6 domains: (A) within-study bias, (B) reporting bias, (C) indirectness, (D) imprecision, (E) heterogeneity, and (F) incoherence. Only the reviewer needs to input the within-study bias and indirectness at the study level. The three levels (no concerns, some concerns, or major concerns) for each domain can be judged according to user-defined rules. The judgments for each domain are eventually summarized to obtain 4 levels of confidence for each pair of comparisons (very low, low, moderate, or high). we examined the certainty of the evidence for the network estimate in line with the following criteria:

**Within-study bias**: CINeMA combines the studies’ contributions with the risk of bias judgments to evaluate within-study bias for each estimate from an NMA. It uses the percentage contribution matrix to approximate the contribution of each study. Then it computes the percentage contribution from studies judged to be at low, moderate, and high risk of bias. We categorized the overall risk of bias in each study. Then based on the results of the Cochrane Risk of Bias version 2 (RoB2) for randomized trials [4], we allocate the values of “low,” “some concern,” and “high,” as 1, 2, and 3, respectively, and input into the CINeMA web application [5].

**Reporting bias**: Although the CINeMA approach suggested some conditions associated with suspected reporting bias, this is still highly subjective. Therefore, by referring to previous NMA studies [6, 7], a comparison-adjusted funnel plot with an accompanying Egger test for asymmetry was calculated.

**Indirectness**: We judged that there was no concern in this domain as the included studies matched our inclusion criteria and study questions.

**Imprecision**: CINeMA compares the treatment effects included in the 95% confidence interval with the range of equivalence. Due to the absence of previous analyses that could be referenced, we considered a clinically meaningful threshold for odds ratios (OR) to be 0.

**Heterogeneity**: As for imprecision, the CINeMA approach to heterogeneity involves comparisons of results with the pre-specified range of clinical equivalence.

**Incoherence**: As for heterogeneity, the CINeMA approach to incoherence considers the impact on clinical implications based on visual inspection of the 95% confidence intervals of direct and indirect ORs and the range of equivalence.

**Summarizing judgments across the 6 domains**: For each pair comparison, one may start at high confidence and drop the confidence level by 1 step for each domain with some concerns, and by 2 levels for each domain with major concerns. However, it is essential to note that domains are interconnected. The 6 CINeMA domains should therefore be considered jointly rather than in isolation, avoiding downgrading the overall level of confidence more than once for related concerns. Therefore, in the GRADE of the current NMA, for the first three domains, the downgrading was no more than two levels; the same was performed for the last three domains.

**References:**

1. Guyatt, G., A. D. Oxman, E. A. Akl, R. Kunz, G. Vist, J. Brozek, S. Norris, Y. Falck-Ytter, P. Glasziou, H. Debeer, R. Jaeschke, D. Rind, J. Meerpohl, P. Dahm, and H. J. Schunemann (2011) GRADE guidelines: 1. Introduction-GRADE evidence profiles and summary of findings tables. J Clin Epidemiol. 64(4): 383-94.

2. Guyatt, G. H., A. D. Oxman, G. E. Vist, R. Kunz, Y. Falck-Ytter, P. Alonso-Coello, and H. J. Schunemann (2008) GRADE: an emerging consensus on rating quality of evidence and strength of recommendations. BMJ. 336(7650): 924-6.

3. Nikolakopoulou, A., Jpt Higgins, T. Papakonstantinou, A. Chaimani, Giovane C. Del, M. Egger, and G. Salanti (2020) CINeMA: An approach for assessing confidence in the results of a network meta-analysis. PLoS Med. 17(4): e1003082.

4. Sterne, Jac, J. Savovic, M. J. Page, R. G. Elbers, N. S. Blencowe, I. Boutron, C. J. Cates, H. Y. Cheng, M. S. Corbett, S. M. Eldridge, J. R. Emberson, M. A. Hernan, S. Hopewell, A. Hrobjartsson, D. R. Junqueira, P. Juni, J. J. Kirkham, T. Lasserson, T. Li, A. Mcaleenan, B. C. Reeves, S. Shepperd, I. Shrier, L. A. Stewart, K. Tilling, I. R. White, P. F. Whiting, and Jpt Higgins (2019) RoB 2: a revised tool for assessing the risk of bias in randomised trials. BMJ. 366: l4898.

5. Papakonstantinou, T., A. Nikolakopoulou, Jpt Higgins, M. Egger, and G. Salanti (2020) CINeMA: Software for semiautomated assessment of the confidence in the results of network meta-analysis. Campbell Syst Rev. 16(1): e1080.

6. Locher, C., J. Kossowsky, H. Koechlin, T. L. Lam, J. Barthel, C. B. Berde, J. Gaab, G. Schwarzer, K. Linde, and K. Meissner (2020) Efficacy, Safety, and Acceptability of Pharmacologic Treatments for Pediatric Migraine Prophylaxis: A Systematic Review and Network Meta-analysis. JAMA Pediatr. 174(4): 341-349.

7. Koechlin, H., J. Kossowsky, T. L. Lam, J. Barthel, J. Gaab, C. B. Berde, G. Schwarzer, K. Linde, K. Meissner, and C. Locher (2021) Nonpharmacological Interventions for Pediatric Migraine: A Network Meta-analysis. Pediatrics. 147(4).

**Pain freedom at 2 hours**


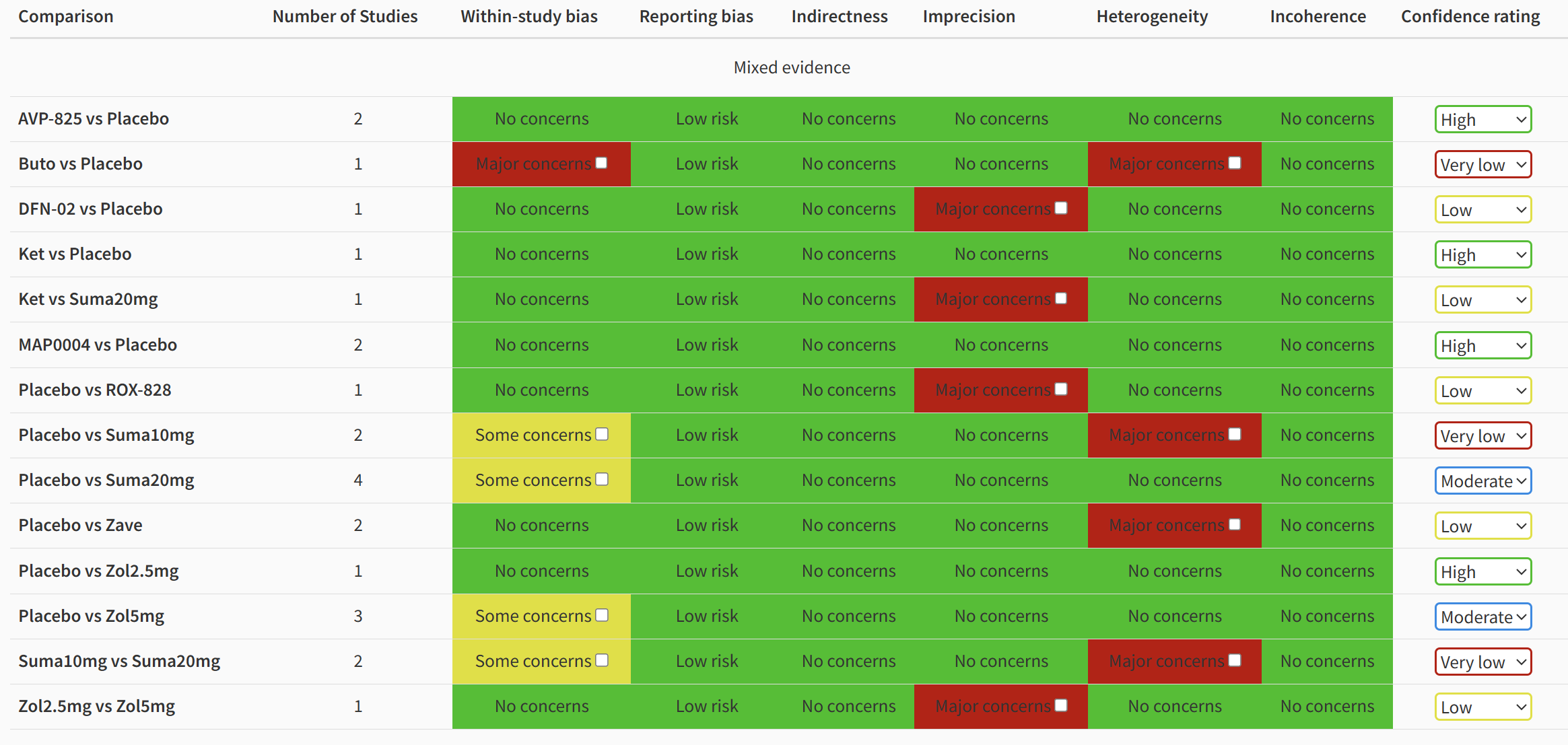


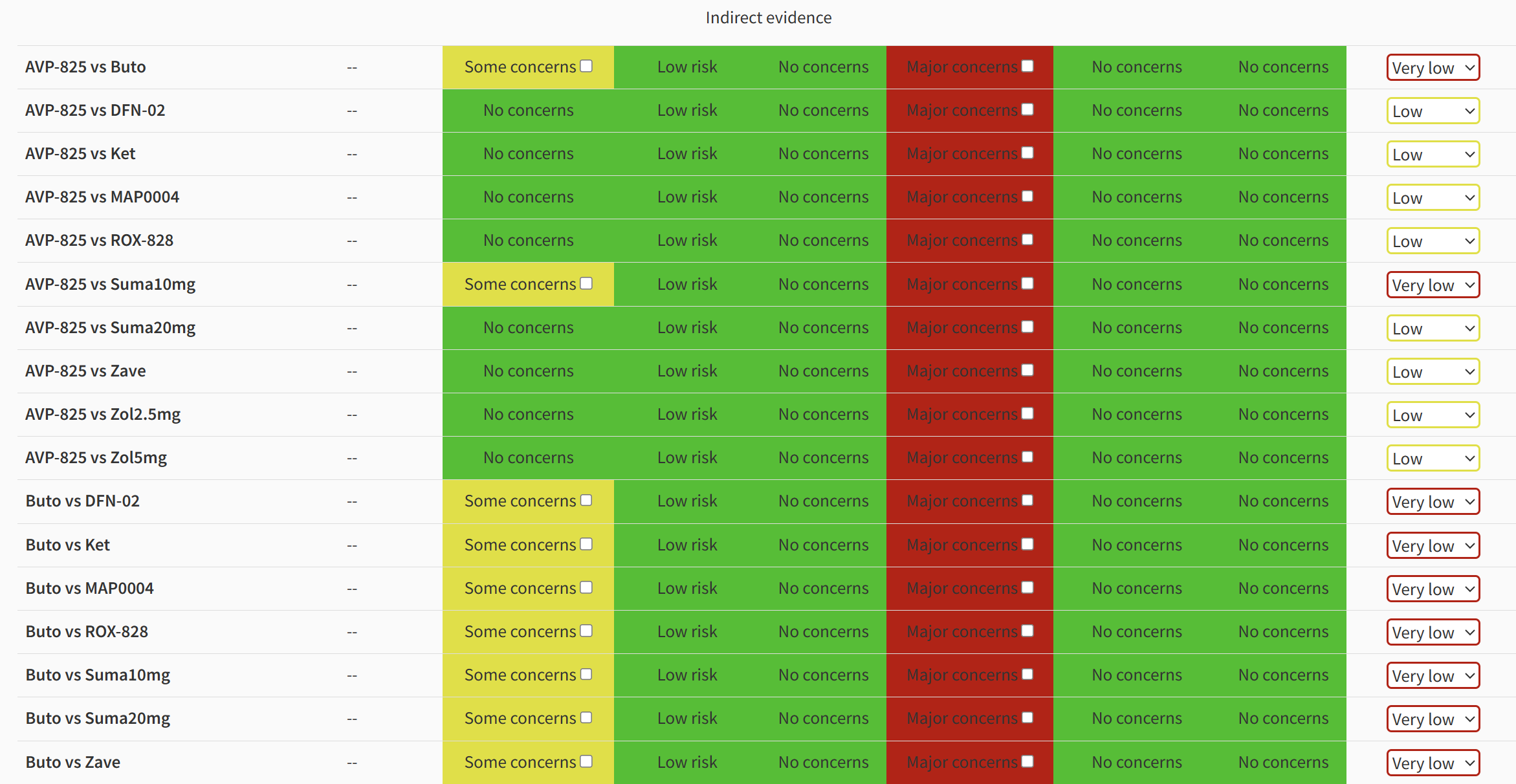


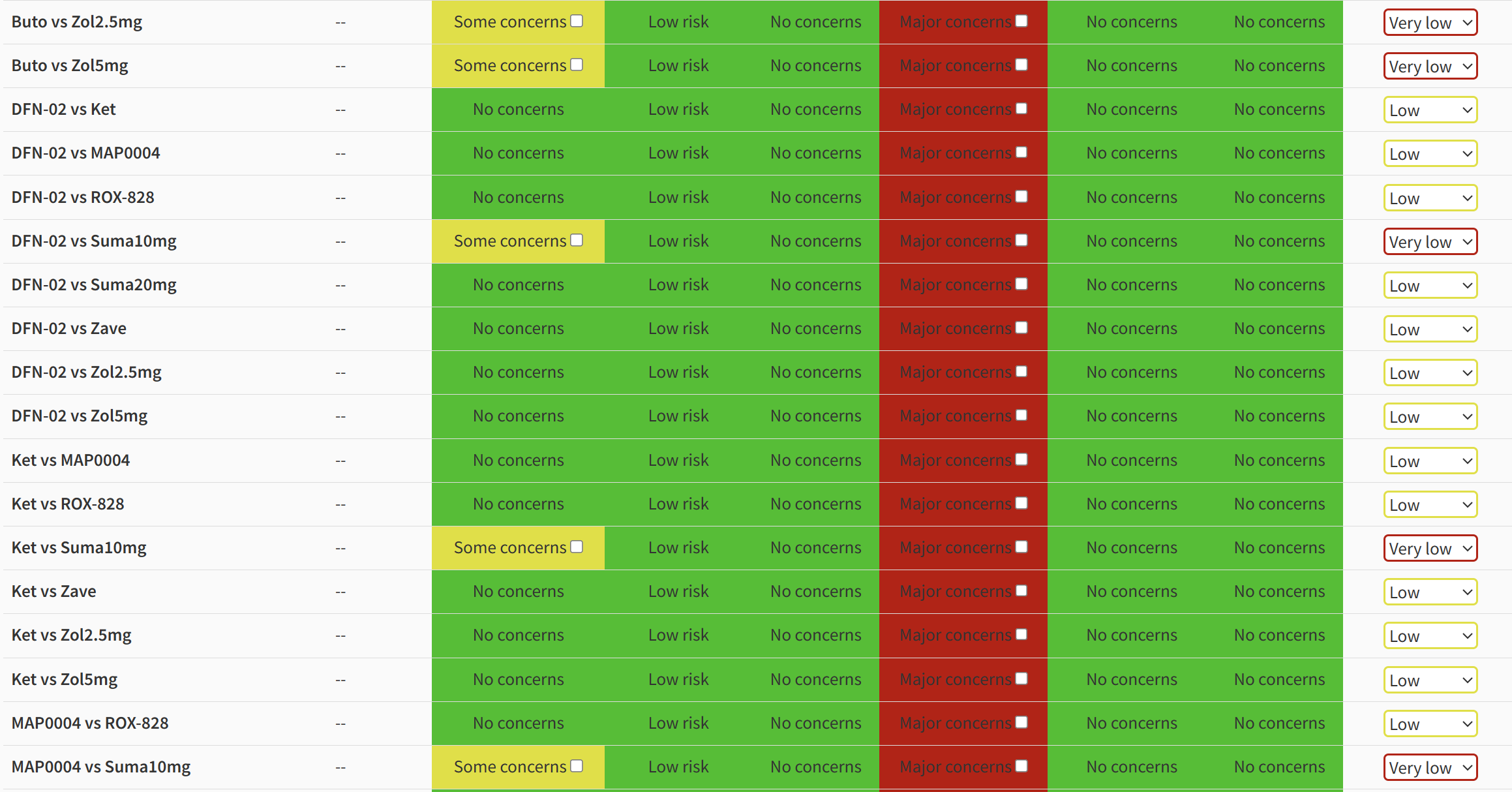


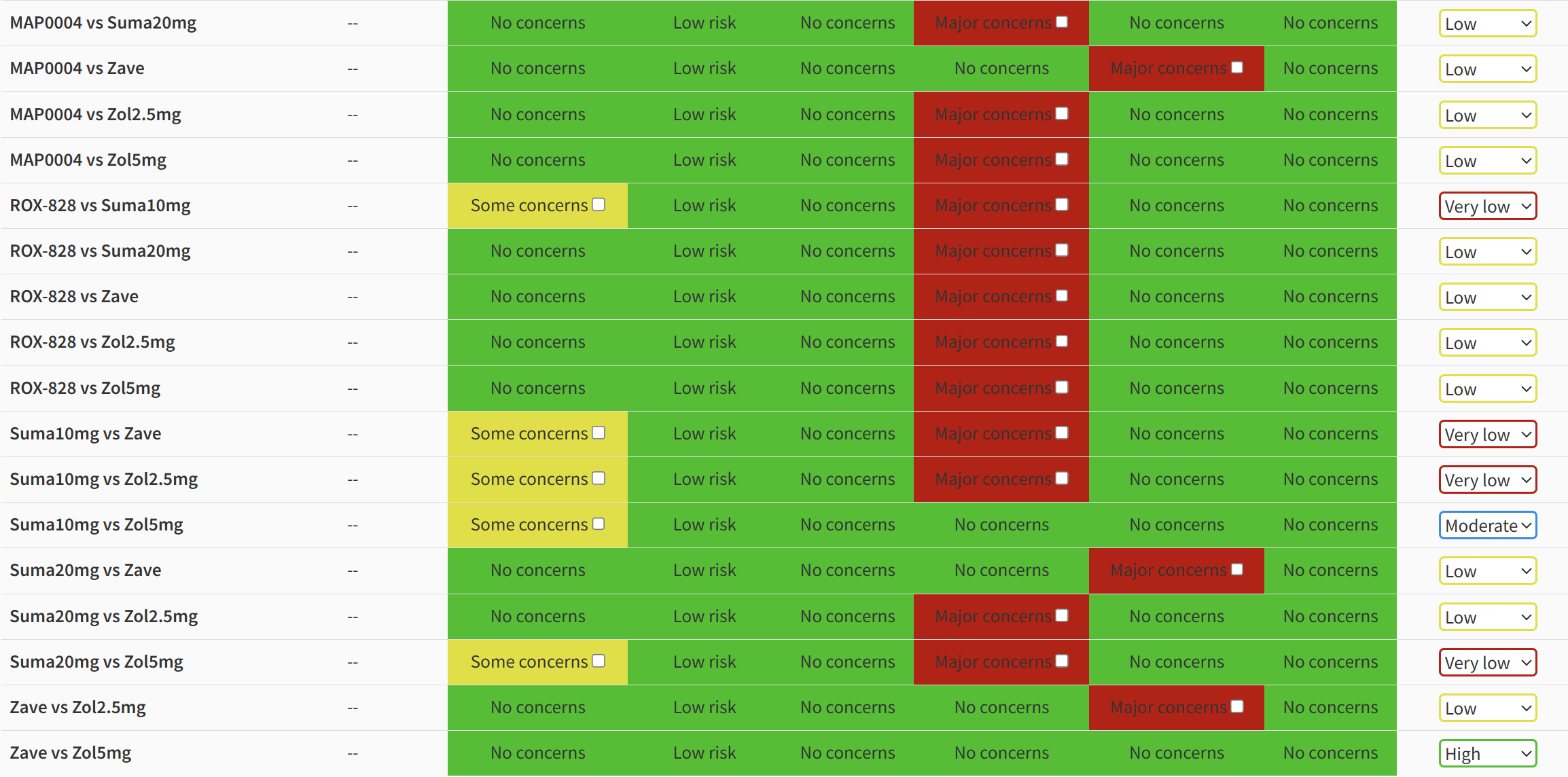


**Pain freedom at 1 hour**


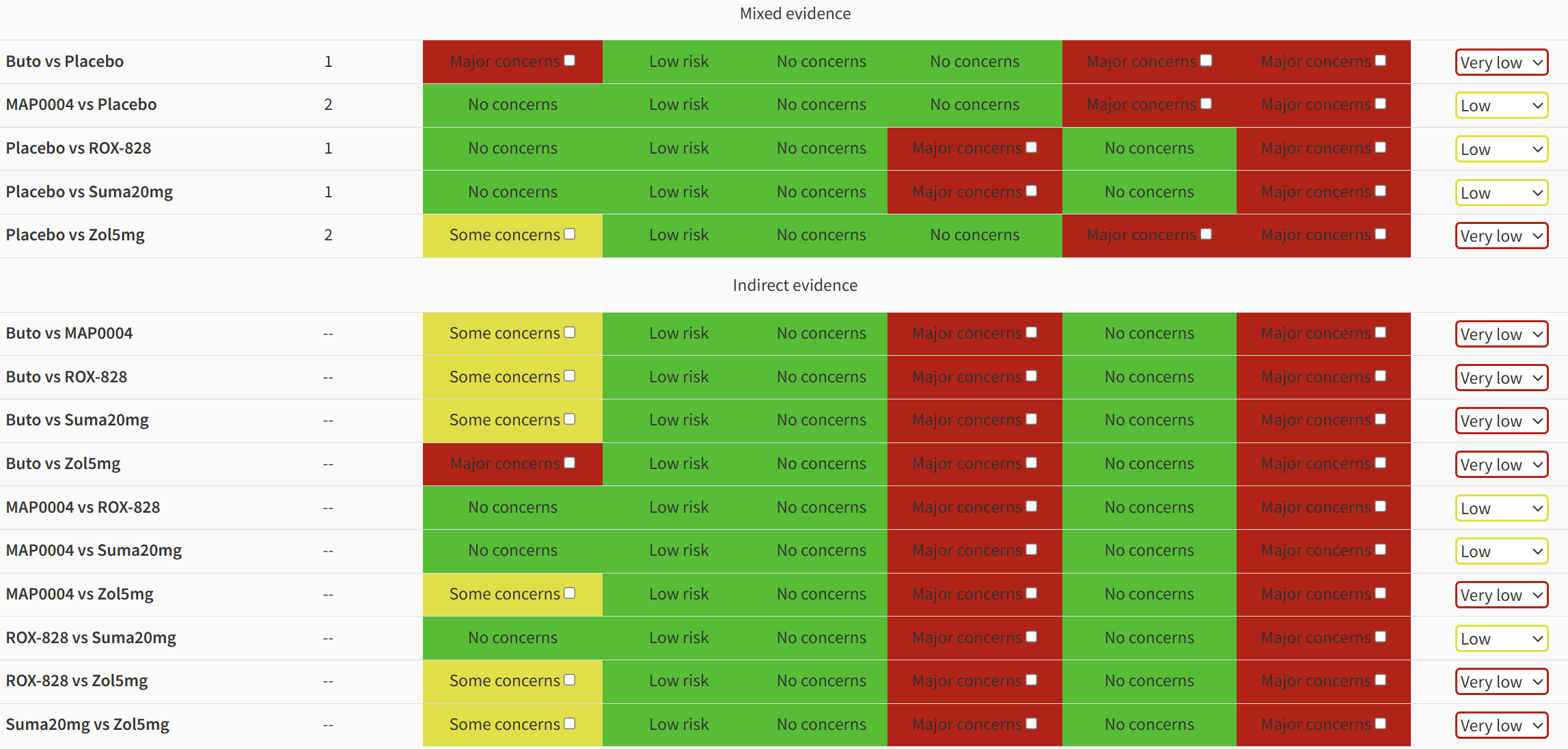


**Sustained pain freedom for 24 hours**


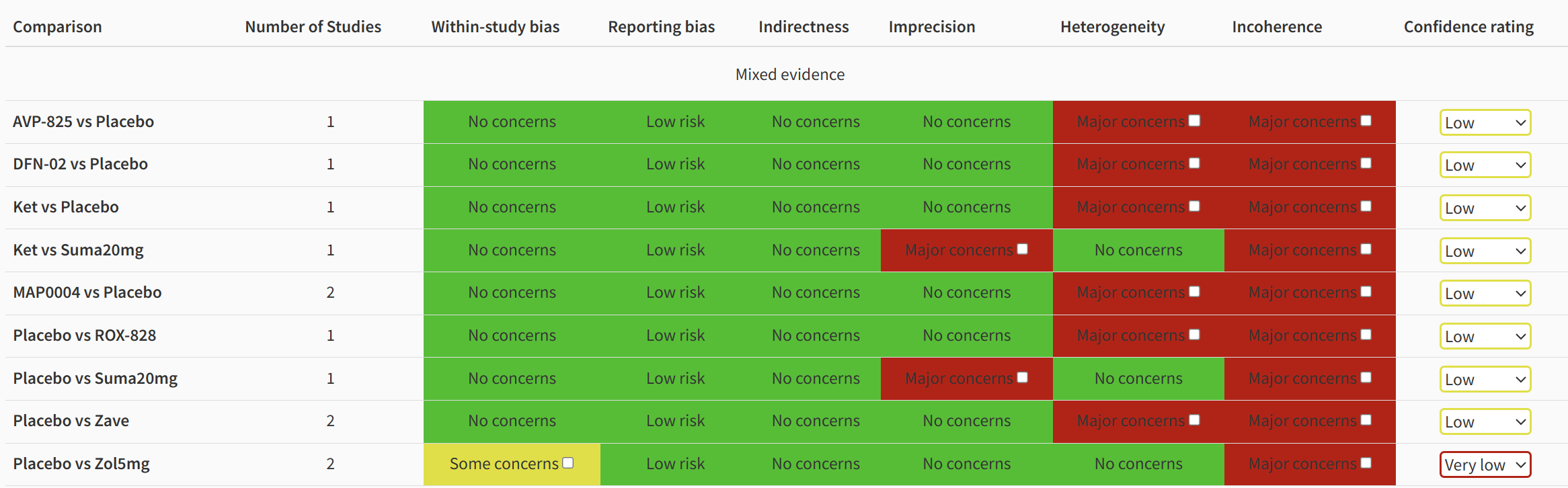


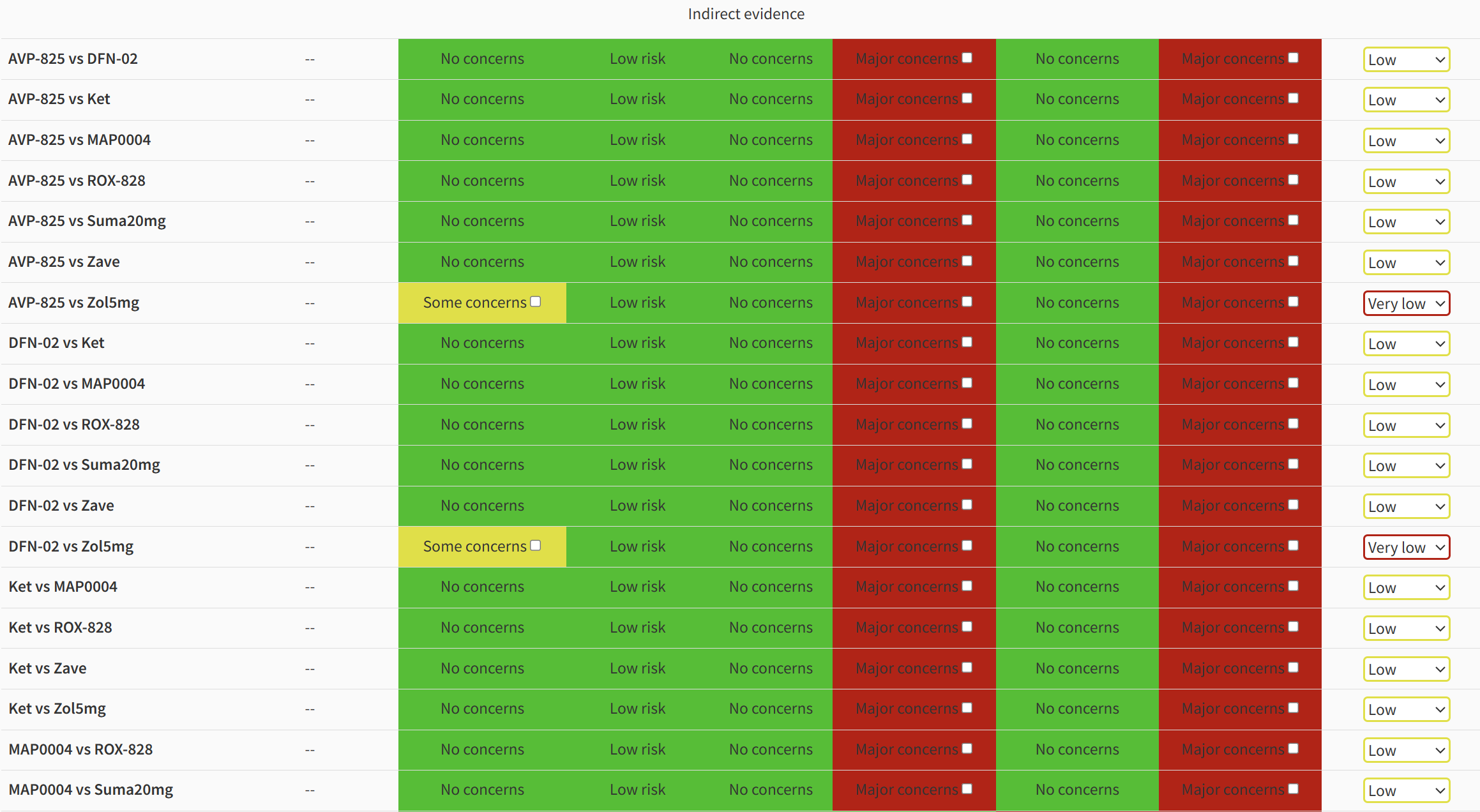


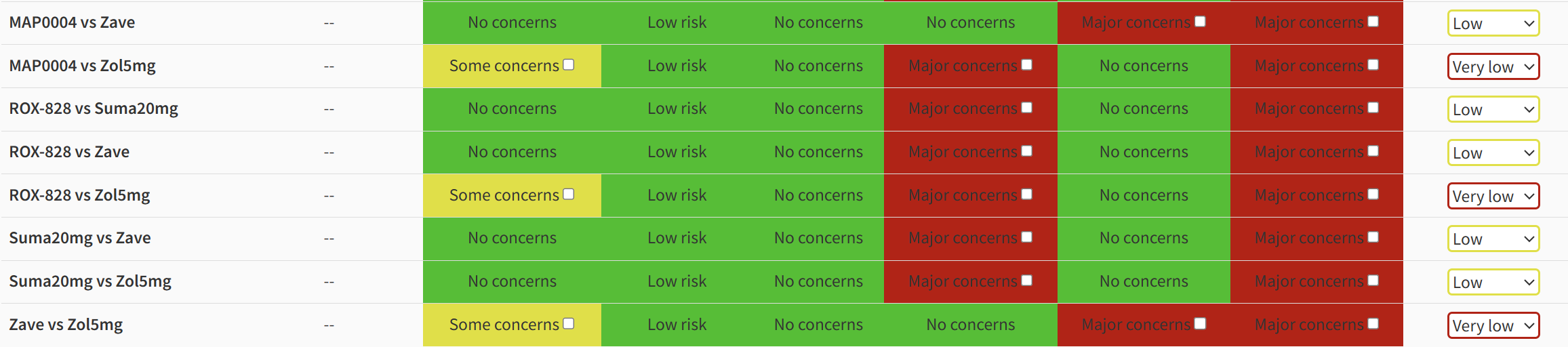


**Freedom from nausea at 2 hours**


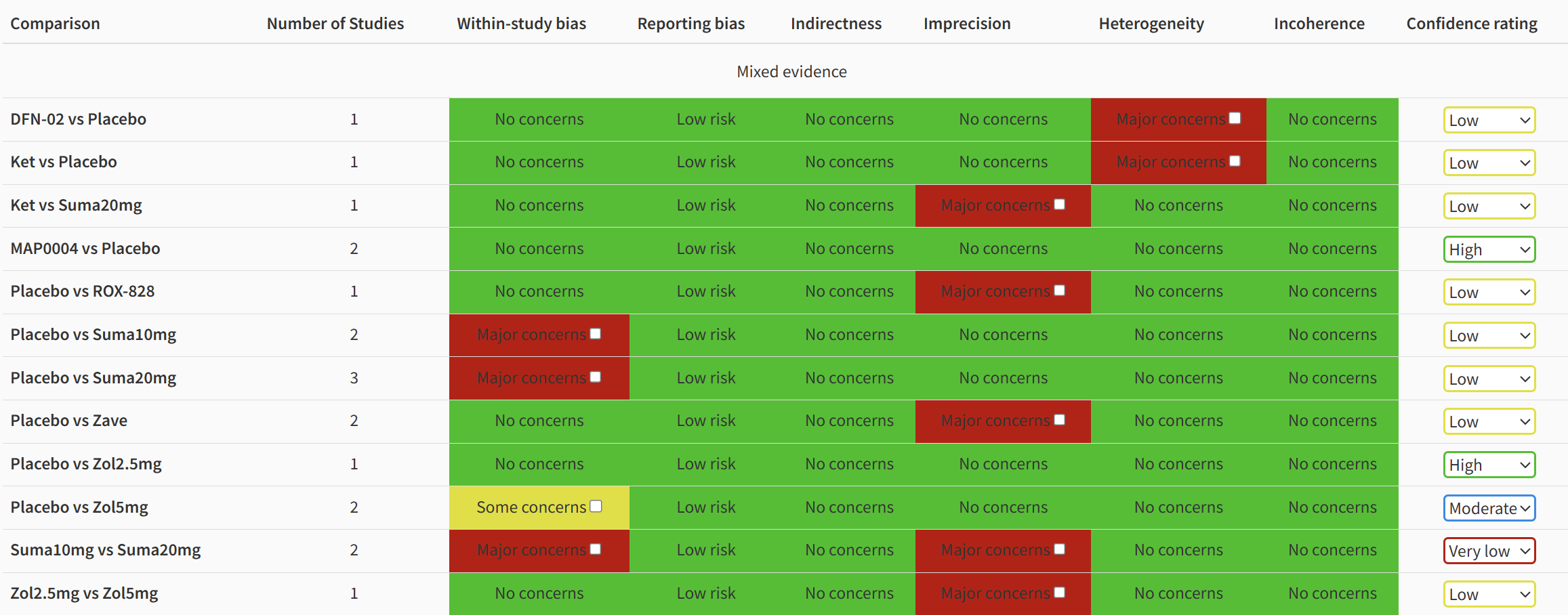


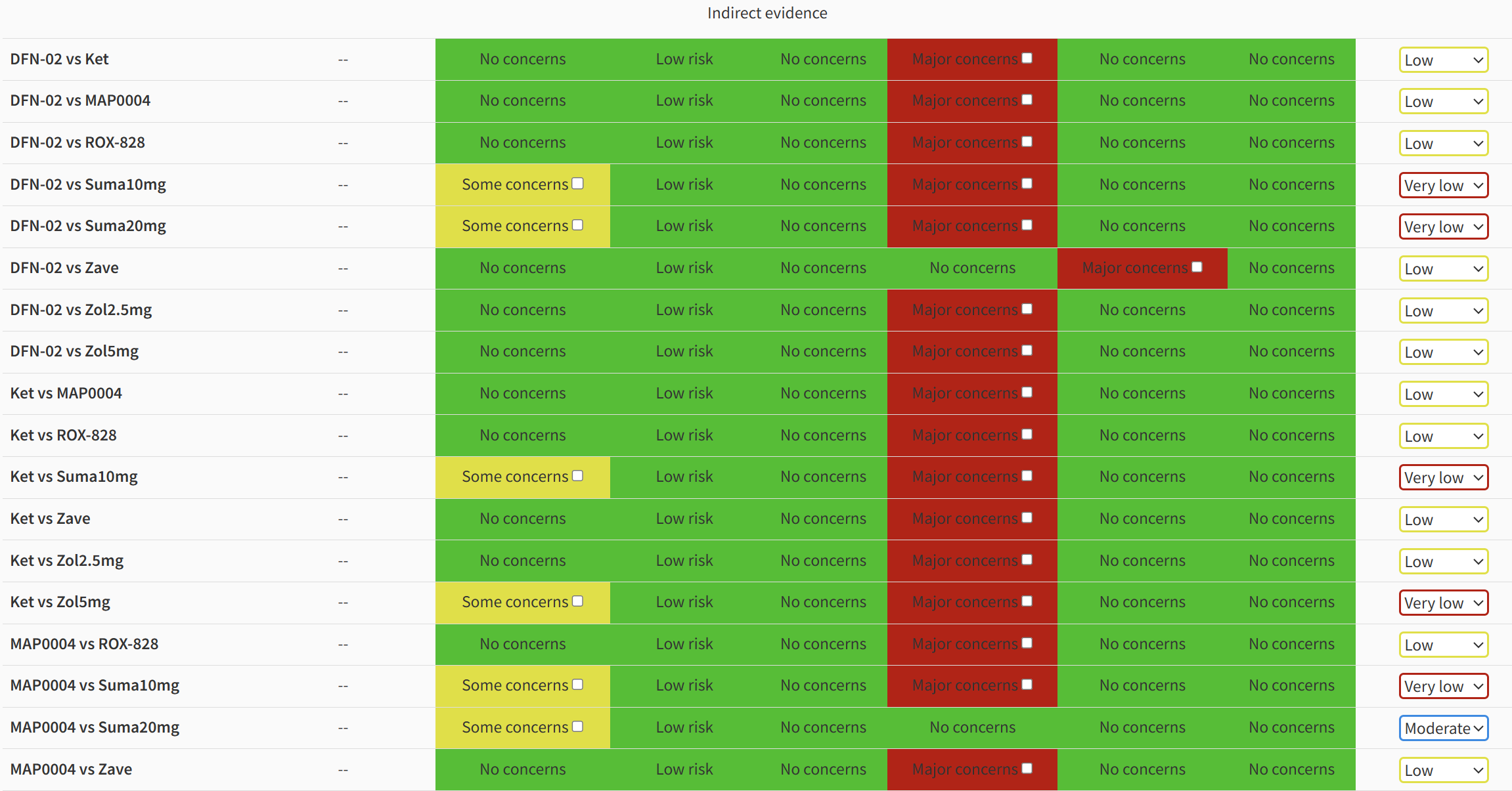


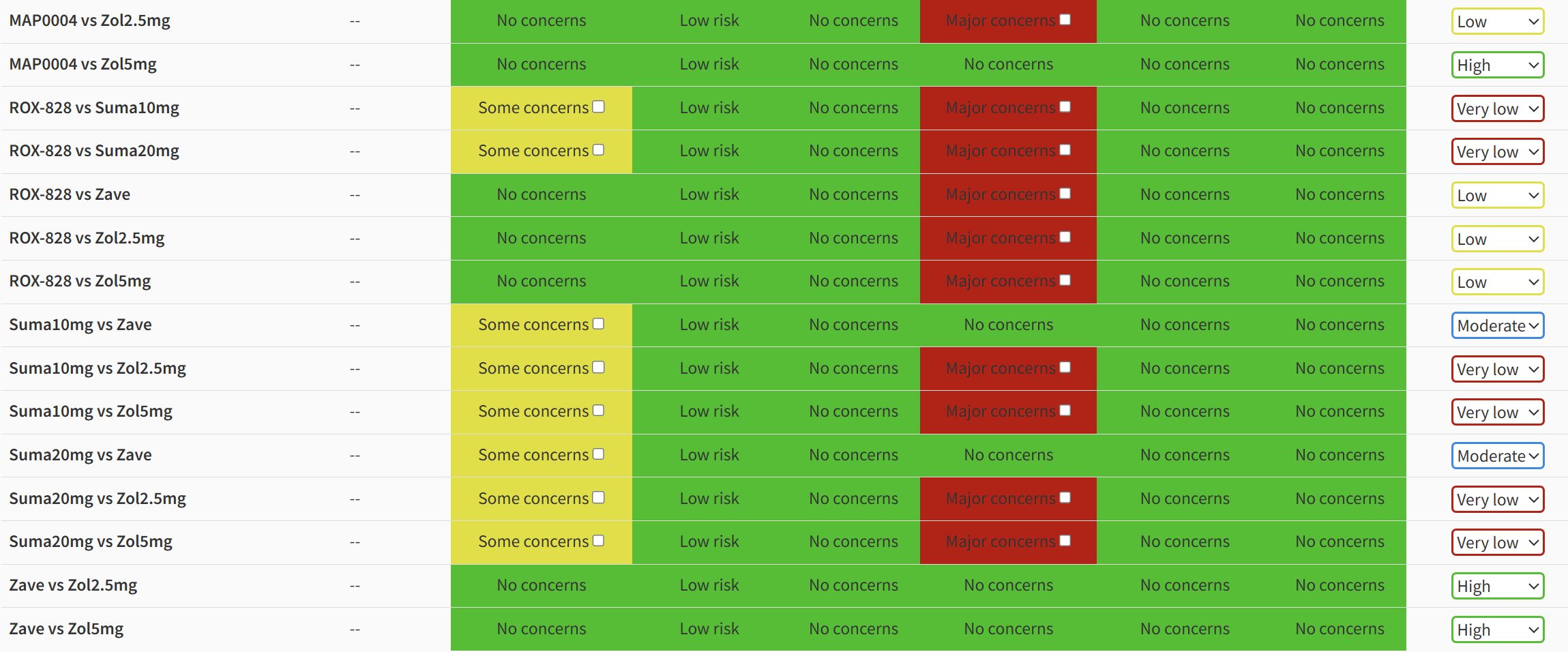


**Freedom from photophobia at 2 hours**


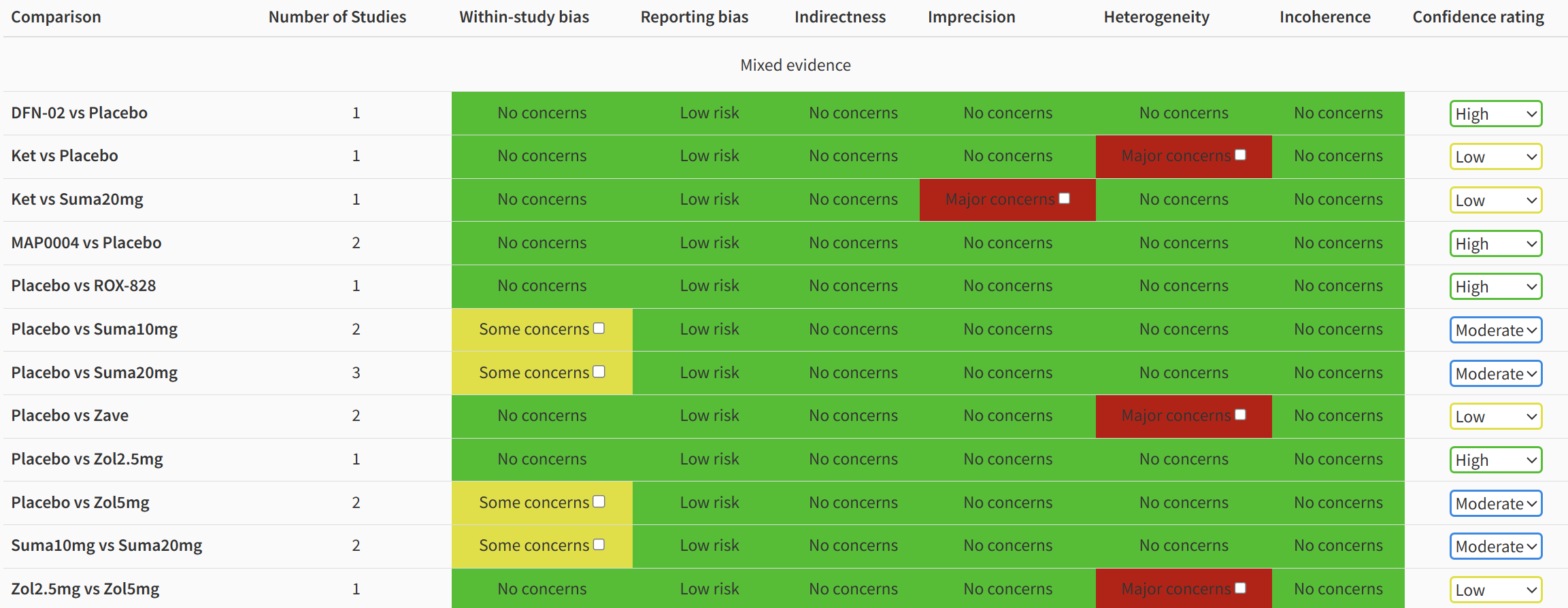


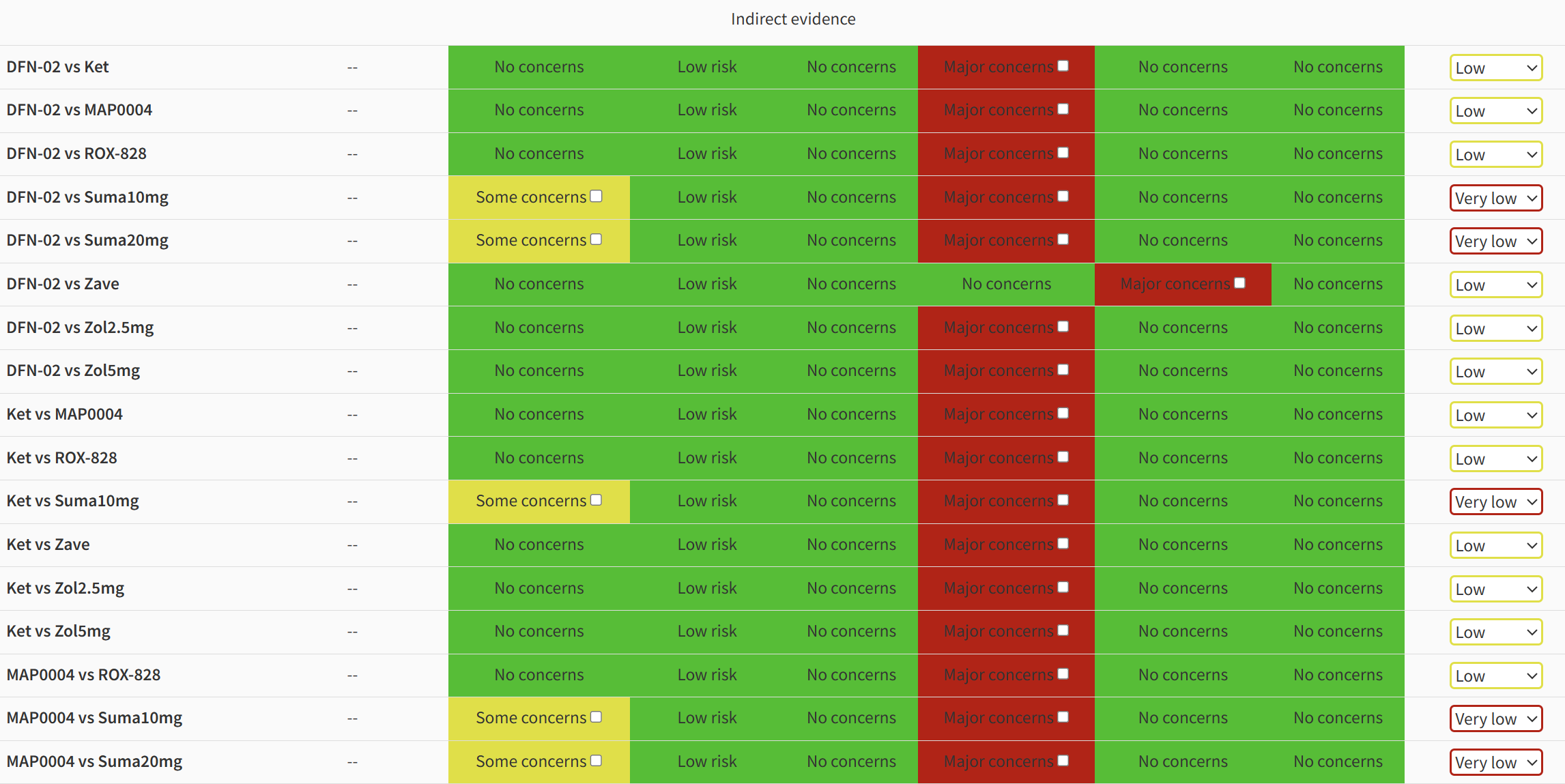


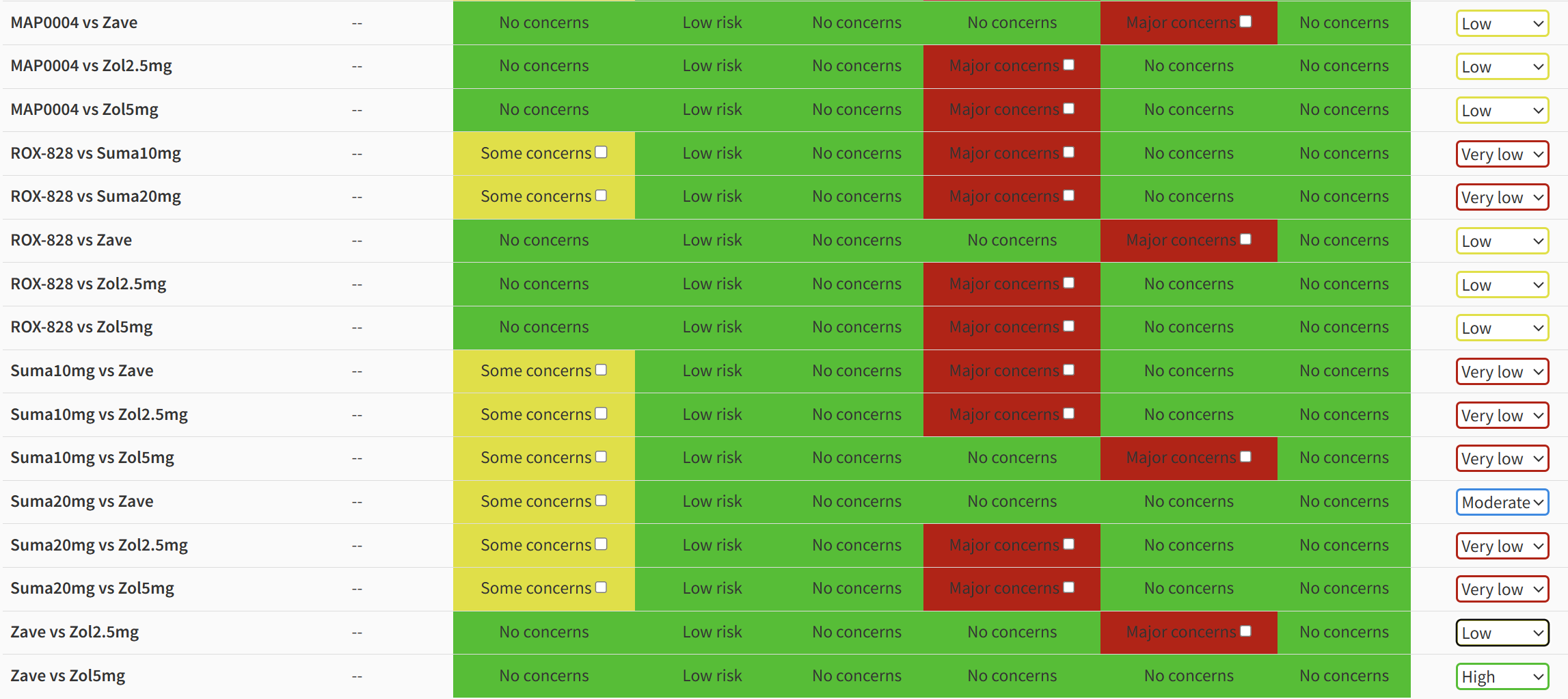


**Freedom from phonophobia at 2 hours**


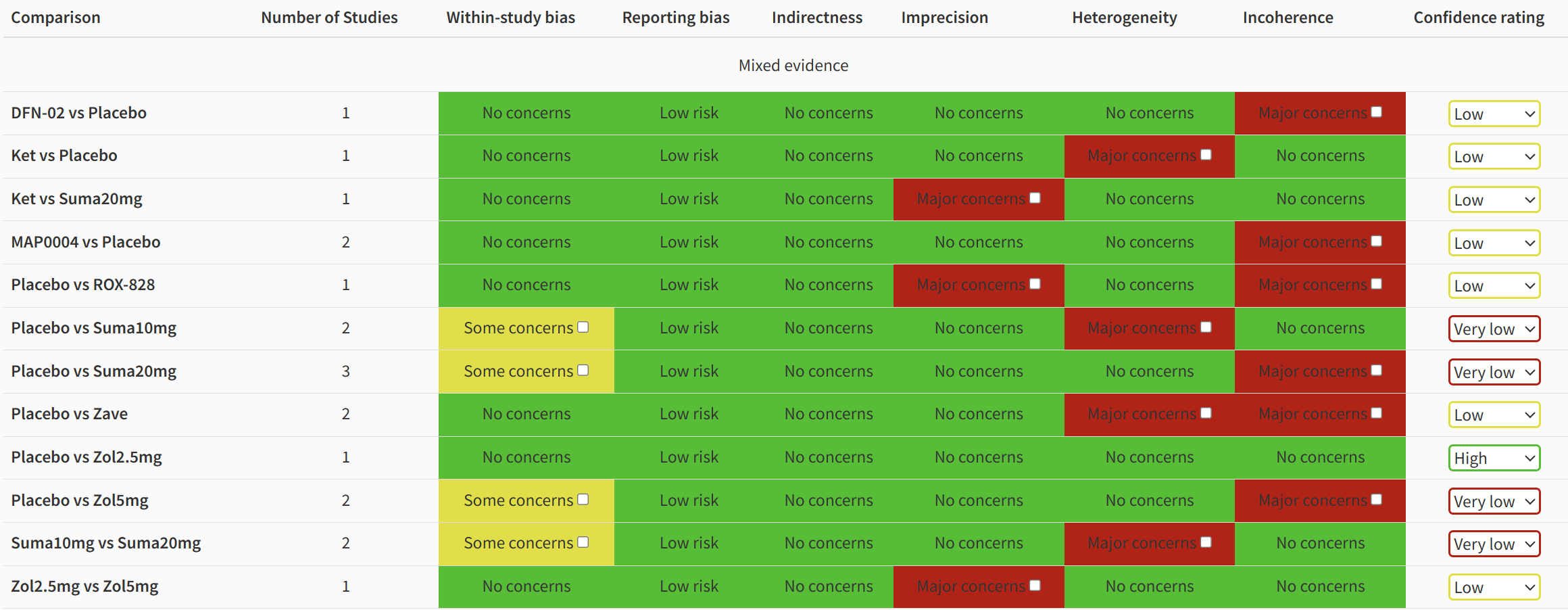


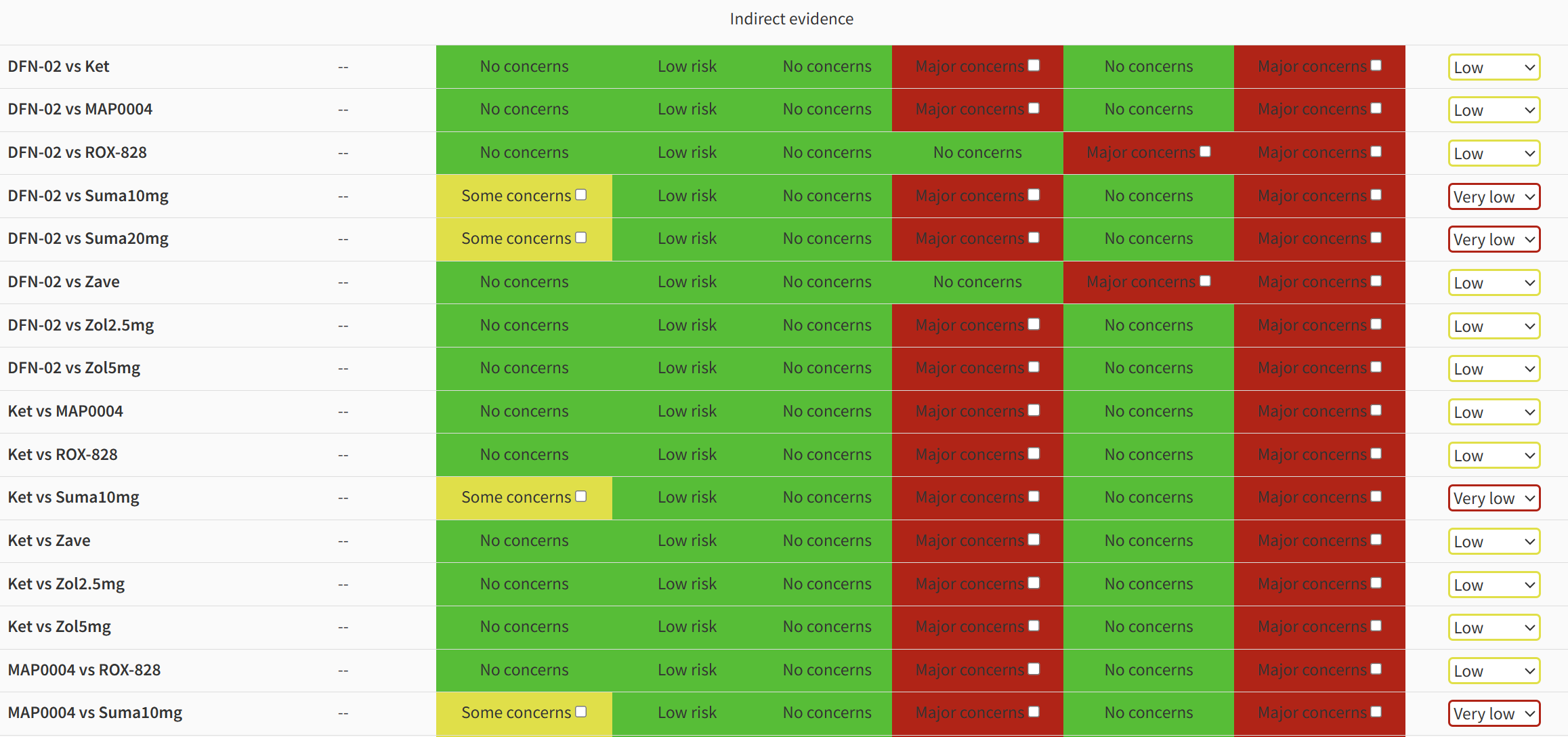


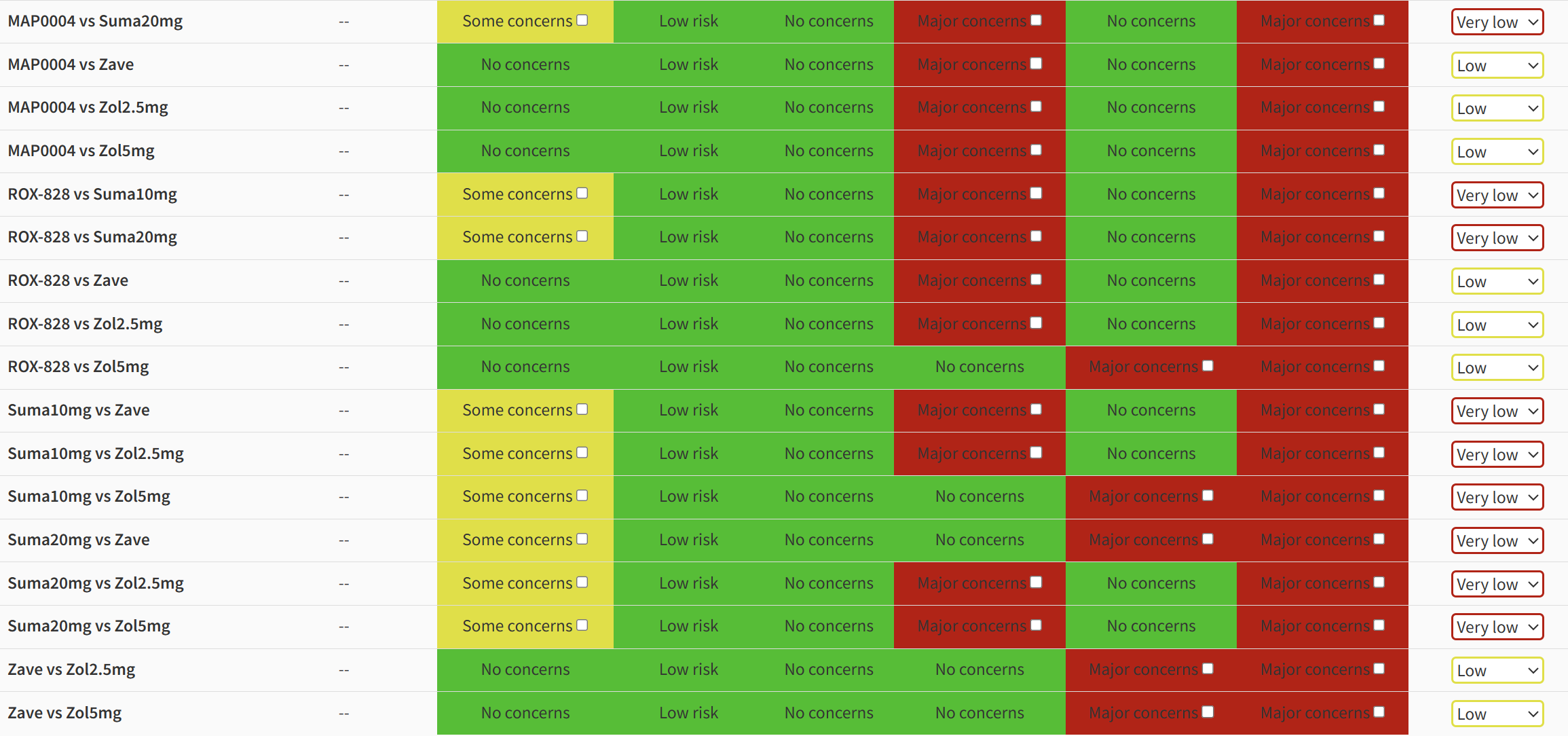


**Adverse events**


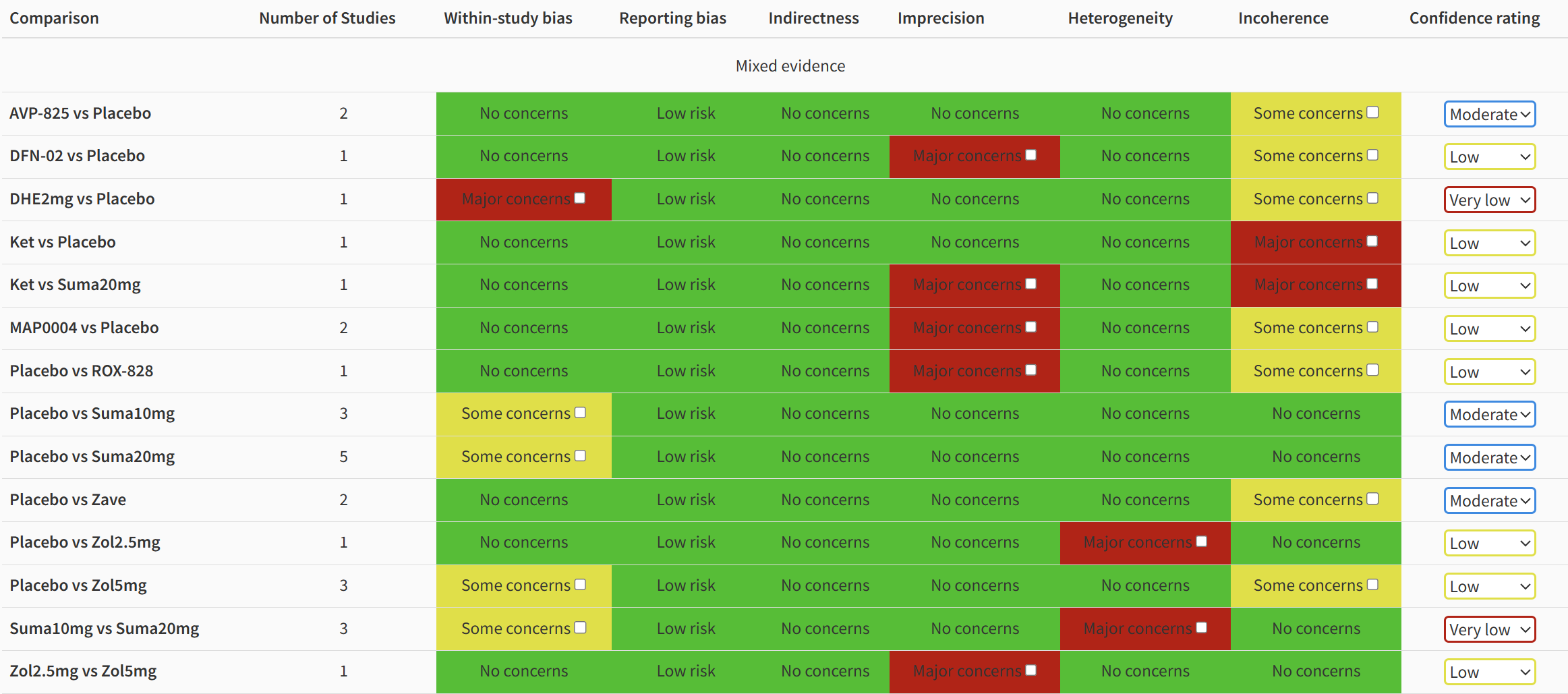


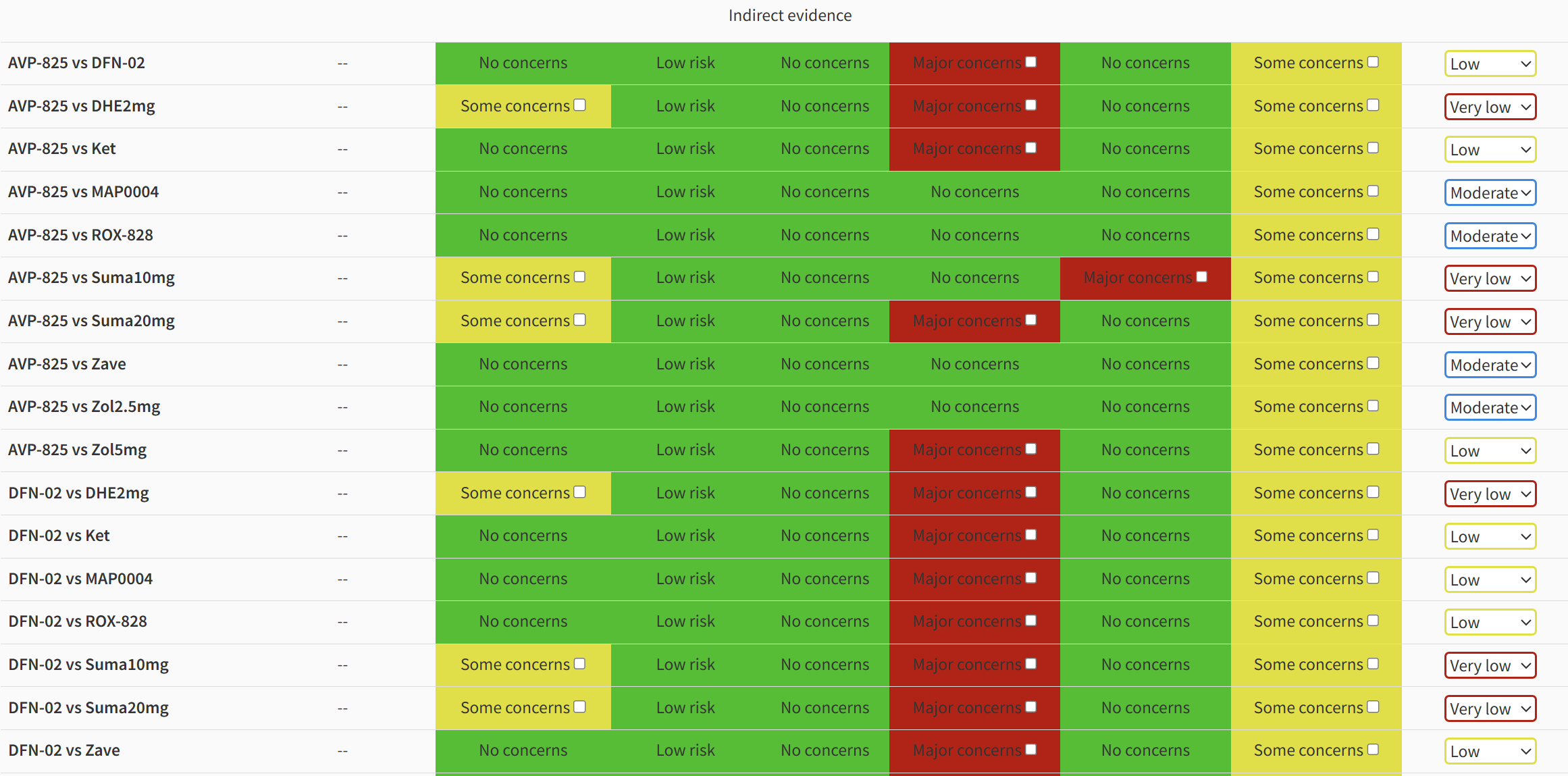


## eTable 1. Baseline demographics characteristics

| **Studies included** | **Country,  Study Design,  Study Period** | **Diagnostic criteria** | **Intervention, Regimens, and routes** | **Sample** | **female, n%** | **Mean age (years, mean ±SD)** | **Pre-treatment headache intensity** | **Aura, n%** | **Use of preventive medications** | **Outcomes** | **Risk of bias** |
| --- | --- | --- | --- | --- | --- | --- | --- | --- | --- | --- | --- |
| **Sumatriptan vs placebo** | |  |  |  |  |  |  |  |  |  |  |
| Richard B Lipton et al.,2018 | RCT in the USA,07/2016 to 02/2017 | ICHD-3 | DFN-02 NS | 93 | 80.6% | 42.3 ± 11.6 | NR | NR | Allowed (No change in prophylactic drug dose within 30 days) | ①③④⑤⑥⑦ | Low |
| Roger K Cady et al.,2015 | RCT in USA,12/2011 to 05/2012 | ICHD-2 | AVP-825 | 212 | 83.5% | 42.0 ± 10.5 | Moderate 83.0%, Severe 17.0% | 35.4% | Allowed (No change in prophylactic drug dose within 30 days) | ①③⑦ | Low |
| P G Djupesland et al.,2010 | RCT in the Czech Republic,04/2007 to 09/2007 | IHS | AVP-825 | 117 | 85.5% | 42 (18 - 64)^*^ | Mild 10.3% Moderate 80.3% Severe 9.4% | 8.5% | Not allowed | ①④ | Low |
| Shuu-Jiun Wang et al.,2007 | RCT in Taiwan | ICHD | Sumatriptan NS 20 mg | 56 | 85.7% | 37.2 ± 10.3 | Moderate 12.5%, Severe 87.5% | 12.5% | Allowed | ①②④ | Low |
| A Peikert et al.,1999 | RCT in multiple countries | IHS | Sumatriptan NS 10 mg, sumatriptan NS 20mg | 584 | 85.0% | 41 ± 9.87 | Mild 0.3% Moderate 54.4% Severe 46.8% | 11.0% | Not allowed | ①④⑤ | Some concerns |
| S Diamond et al.,1998 | RCT in the USA | IHS | Sumatriptan NS 10 mg, sumatriptan NS 20mg | 1086 | 88.0% | 41.0^*^ | NR | 30.0% | Allowed | ④⑤⑥⑦ | High |
| R Ryan et al.,1997 | RCT | IHS | Sumatriptan NS 10mg, sumatriptan NS 20mg | 845 | 86.0% | 40.7^*^ | NR | 13.1% | Allowed | ①④⑥⑦ | Some concerns |
| Salonen, R et al.,1994 | RCT in multiple countries | IHS | Sumatriptan NS 10mg, sumatriptan NS 20mg | 455 | 80.0% | 41.9 ± 10.2 | Mild 1.2% Moderate 50.2% Severe 48.6% | 11.0% | Not allowed |  | High |
| **Zolmitriptan vs placebo** | |  |  |  |  |  |  |  |  |  |  |
| David Dodick et al.,2005 | RCT in the USA | IHS | Zolmitriptan NS 5mg | 1869 | 86.6% | 40.7 ± 10.3 | NR | 18.4% | Not allowed | ①②③④⑤⑥⑦ | Some concerns |
| Marek Gawel et al.,2005 | RCT in Canada and Germany | IHS | Zolmitriptan NS 5mg | 912 | 87.5% | 41.7 (18 - 66)^*^ | Mild 8% Moderate 66.2% Severe 25.3% | NR | NR | ①②③⑦ | Some concerns |
| Bruce R Charlesworth et al.,2003 | RCT at 42 centers in 11 countries,12/1997 to 02/1999 | IHS | Zolmitriptan NS 2.5mg, zolmitriptan NS 5.0mg | 1372 | 83.0% | 40.6 ± 10.3 | NR | 37.9% | Not allowed | ①③④⑤⑥⑦ | Low |
| **Zavegepant vs placebo** | |  |  |  |  |  |  |  |  |  |  |
| Robert Croop et al.,2022 | RCT in USA，03/2019 to 09/2019 | ICHD-3 | Zavegepant NS10mg | 1581 | 85.5% | 40.8 ± 12.7 | NR | NR | Allowed (No change in prophylactic drug dose within 90 days and the dose will not change during the study) | ①③④⑤⑥⑦ | Low |
| Richard B Lipton et al.,2023 | RCT in the USA，10/2020 to 08/2021 | ICHD-3 | Zavegepant NS 10mg | 1269 | 82.8% | 40.9 ± 13.2 | NR | 34.2% | Allowed (No change in prophylactic drug dose within 90 days) | ①③④⑤⑥⑦ | Low |
| **Ketorolac vs placebo** | |  |  |  |  |  |  |  |  |  |  |
| Aruna S Rao et al.,2016 | RCT in the USA，03/2013 to 12/2014 | ICHD-2 | Ketorolac NS 31.5mg, sumatriptan NS 20mg | 152 | 98.1% | 36.3 ± 9.8 | Mild 37.5% Moderate/Severe 42% | 28.6% | Allowed (No change in prophylactic drug dose within 90 days) | ①③④⑤⑥⑦ | Some concerns |
| V Pfaffenrath et al.,2012 | RCT in Germany andFinland,09/2007 to 02/2008 | IHS | ROX-828 | 140 | 92.1% | 40 (18-60)^*^ | Moderate 68.6% Severe 27.9% | 27.9% | Allowed (No change in prophylactic drug dose within 12 weeks) | ①②③④⑤⑥⑦ | Low |
| **Dihydroergotamine vs placebo** | |  | |  | |  | |  | |  | |
| R M Gallagher et al.,1996 | RCT in the USA, 04/1993 to  06/1994 | IHS | Dihydroergotamine NS 2mg | 308 | NR | 40 ± 7.8 | NR | NR | Not allowed | ④ | High |
| Sheena K Aurora et al.,2009 | RCT in the USA, 07/2006 to  02/2007 | ICHD-2 | MAP0004 1mg | 86 | 80.7% | 41.2 ± 10.8 | NR | NR | NR | ①②③④⑤⑥⑦ | Low |
| Sheena K Aurora et al.,2011 | RCT in the USA, 07/2008 to  03/2009 | ICHD-2 | MAP0004 1mg | 903 | 80.3% | 40.0 ± 11.5 | Moderate 53.8% Severe 46.2% | NR | Allowed | ①②③④⑤⑥⑦ | Low |
| **Butorphanol vs placebo** | |  | |  | |  | |  | |  | |
| M J Hoffert et al.,1995 | RCT in the USA | IHS | Butorphanol NS 1 mg | 157 | 84.1% | 40.8±8.0 | NR | NR | Allowed | ①② | High |
| IHS, international headache society; ICHD, International Classification of Headache Disorders; ICHD-2, International Classification of Headache Disorders, 2nd edition; ICHD-3, International Classification of Headache Disorders, 3rd edition; NS, Nasal Spray; NR, not reported; *, median (IQR); ①, pain freedom at 2 hours; ②, pain freedom at 1 hour; ③, sustained pain freedom for 24 hours; ④, adverse events; ⑤, freedom from nausea at 2 hours;⑥, freedom from photophobia at 2 hours; ⑦, freedom from phonophobia after 2 hours. DFN-02, sumatriptan nose spray 10 mg with a permeation enhancer; AVP-825, a drug-device combination of 22mg sumatriptan powder; MAP0004, a dihydroergotamine inhaler; ROX-828, ketorolac 31.5mg with 6% of lidocaine. | | | | | | | | | | |  |

**eTable 2:** SUCRA of pain freedom at 2 hours

| **Treatment** | **SUCRA, n%** |
| --- | --- |
| Zolmitriptan 5mg | 87.1 |
| Ketorolac 31.5mg | 70.3 |
| MAP0004 1mg | 68.9 |
| Butorphanol 1mg | 66.9 |
| Sumatripan20mg | 65.2 |
| Zolmitriptan 2.5mg | 56.6 |
| AVP-825 22mg | 56.2 |
| DFN-02 10mg | 48.6 |
| ROX-828 31.5mg | 33.3 |
| Sumatripan10mg | 25.8 |
| Zavegepant 10mg | 19.7 |
| Placebo | 1.4 |

SUCRA values vary from 0 to 1, whereas 1 means the treatment might be the best and 0 the worst.

**eTable 3:** SUCRA of adverse events

| **Treatment** | **SUCRA, n%** |
| --- | --- |
| Placebo | 95.1 |
| ROX-828 31.5mg | 85.9 |
| MAP0004 1mg | 81.9 |
| Zavegepant 10mg | 66.2 |
| Zolmitriptan 2.5mg | 65.9 |
| Sumatripan 10mg | 59.4 |
| Zolmitriptan 5mg | 40.5 |
| Sumatripan 20mg | 37.5 |
| DFN-02 10mg | 22.8 |
| AVP-825 22mg | 19.4 |
| Ketorolac 31.5mg | 13.5 |
| Dihydroergotamine 2mg | 11.9 |

SUCRA values vary from 0 to 1, whereas 1 means the treatment might be the best and 0 the worst.

**eTable 4:** SUCRA of freedom from nausea at 2 hours

| **Treatment** | **SUCRA, n%** |
| --- | --- |
| DFN-02 10mg | 87 |
| Ketorolac 31.5mg | 76.7 |
| Sumatripan20mg | 74.6 |
| Zolmitriptan 5mg | 64.7 |
| ROX-828 31.5mg | 58.3 |
| Sumatripan10mg | 55.4 |
| Zolmitriptan 2.5mg | 42.1 |
| MAP0004 1mg | 26.9 |
| Zavegepant 10mg | 10.6 |
| Placebo | 3.7 |

SUCRA values vary from 0 to 1, whereas 1 means the treatment might be the best and 0 the worst.

**eTable 5:** SUCRA of freedom from photophobia at 2 hours

| **Treatment** | **SUCRA, n%** |
| --- | --- |
| ROX-828 31.5mg | 82.1 |
| DFN-02 10mg | 80.3 |
| Zolmitriptan 5mg | 76.8 |
| Sumatripan20mg | 69.4 |
| Ketorolac 31.5mg | 55.6 |
| MAP0004 1mg | 46.9 |
| Zolmitriptan 2.5mg | 45.8 |
| Sumatripan10mg | 28.3 |
| Zavegepant 10mg | 14.4 |
| Placebo | 0.4 |

SUCRA values vary from 0 to 1, whereas 1 means the treatment might be the best and 0 the worst.

**eTable 6:** SUCRA of freedom from phonophobia at 2 hours

| **Treatment** | **SUCRA, n%** |
| --- | --- |
| DFN-02 10mg | 82.1 |
| Zolmitriptan 5mg | 76.8 |
| Ketorolac 31.5mg | 74.5 |
| Zolmitriptan 2.5mg | 71.5 |
| Sumatripan20mg | 63.3 |
| MAP0004 1mg | 46.3 |
| Sumatripan10mg | 34.8 |
| Zavegepant 10mg | 24.5 |
| ROX-828 31.5mg | 13.0 |
| Placebo | 5.6 |

SUCRA values vary from 0 to 1, whereas 1 means the treatment might be the best and 0 the worst.

**eTable 7:** SUCRA of sustained pain freedom for 24 hours

| **Treatment** | **SUCRA, n%** |
| --- | --- |
| Zolmitriptan 5mg | 85.6 |
| DFN-02 10mg | 72.0 |
| Ketorolac 31.5mg | 66.4 |
| MAP0004 1mg | 66.2 |
| AVP-825 22mg | 50.9 |
| ROX-828 31.5mg | 50.4 |
| Sumatripan20mg | 33.1 |
| Zavegepant 10mg | 23.4 |
| Placebo | 1.9 |

SUCRA values vary from 0 to 1, whereas 1 means the treatment might be the best and 0 the worst.

**eTable 8:** SUCRA of pain freedom at 1 hour

| **Treatment** | **SUCRA, n%** |
| --- | --- |
| Butorphanol 1mg | 81.6 |
| Sumatripan20mg | 61.6 |
| MAP0004 1mg | 59.1 |
| ROX-828 31.5mg | 48.4 |
| Zolmitriptan 5mg | 47.0 |
| Placebo | 2.3 |

SUCRA values vary from 0 to 1, whereas 1 means the treatment might be the best and 0 the worst.

**eTable9:** Design-by-treatment interaction model for inconsistency of network meta-analysis

| **design-by-treatment inconsistency** | **chi2** | **Prob>chi2** |
| --- | --- | --- |
| pain freedom at 2 hours | 3.30 | 0.35 |
| pain freedom at 1 hour | No potential source of inconsistency | |
| sustained pain freedom for 24 hours | No potential source of inconsistency | |
| freedom from nausea at 2 hours | 1.01 | 0.629 |
| freedom from photophobia at 2 hours | 2.16 | 0.3395 |
| freedom from phonophobia after 2 hours | 6.65 | 0.036 |
| adverse events | 5.92 | 0.1155 |

**eTable 10:** Significant loop-specific inconsistencies of network meta-analysis

| **loop inconsistency** | **Ratio of odds ratios** | **95% confidence interval** | **Tau^2^** | **P** |
| --- | --- | --- | --- | --- |
| **pain freedom at 2 hours** |  |  |  |  |
| Zolmitriptan2.5mg-Zolmitriptan5mg-placebo | 1.740 | (1.01,3.00) | 0.000 | 0.047 |
| Sumatripan10mg-Sumatripan20mg-placebo | 1.535 | (1.00,3.87) | 0.000 | 0.908 |
| Ketorolac 31.5mg-Sumatripan20mg-placebo | 1.284 | (1.00,4.55) | 0.000 | 0.387 |
| **freedom from nausea at 2 hours** |  |  |  |  |
| Sumatripan10mg-Sumatripan20mg-placebo | 1.594 | (1.00,6.03) | 0.000 | 0.492 |
| Ketorolac 31.5mg-Sumatripan20mg-placebo | 1.564 | (1.00,11.41) | 0.000 | 0.659 |
| Zolmitriptan2.5mg-Zolmitriptan5mg-placebo | 1.135 | (1.00,1.76) | 0.000 | 0.572 |
| **freedom from photophobia at 2 hours** |  |  |  |  |
| Sumatripan10mg-Sumatripan20mg-placebo | 1.402 | (1.00,3.67) | 0.000 | 0.491 |
| Ketorolac 31.5mg-Sumatripan20mg-placebo | 1.410 | (1.00,5.09) | 0.000 | 0.599 |
| Zolmitriptan2.5mg-Zolmitriptan5mg-placebo | 1.365 | (1.00,2.11) | 0.000 | 0.161 |
| **freedom from phonophobia after 2 hours** |  |  |  |  |
| Sumatripan10mg-Sumatripan20mg-placebo | 1.816 | (1.00,4.98) | 0.000 | 0.246 |
| Ketorolac 31.5mg-Sumatripan20mg-placebo | 1.830 | (1.00,7.69) | 0.000 | 0.409 |
| Zolmitriptan2.5mg-Zolmitriptan5mg-placebo | 1.552 | (1.00,2.42) | 0.000 | 0.053 |
| **adverse events** |  |  |  |  |
| Sumatripan10mg-Sumatripan20mg-placebo | 2.078 | (1.00,7.43) | 0.118 | 0.261 |
| Ketorolac 31.5mg-Sumatripan20mg-placebo | 4.383 | (1.00,37.09) | 0.257 | 0.175 |
| Zolmitriptan2.5mg-Zolmitriptan5mg-placebo | 1.082 | (1.00,1.90) | 0.000 | 0.275 |

pain freedom at 1 hour and sustained pain freedom for 24 hours was not potentially inconsistency.

**eTable 11:** Significant side-splitting inconsistencies of network meta-analysis

|  | **Direct** |  | **Indirect** |  | **Difference** |  | **P>z** | **Tau** | **Treatments used** |  |
| --- | --- | --- | --- | --- | --- | --- | --- | --- | --- | --- |
| **Side** | **Coefficient** | **SE** | **Coefficient** | **SE** | **Coefficient** | **SE** |  |  |  |  |
| **pain freedom at 2 hours** |  |  |  |  |  |  |  |  |  |  |
| A vs. L | -0.99 | 0.52 | -0.75 | 194.33 | -0.24 | 194.33 | 0.999 | 0.20 | A: | DFN-02 10mg |
| B vs. F | -0.18 | 0.45 | 0.36 | 1.04 | -0.54 | 1.14 | 0.636 | 0.21 | B: | Ketorolac 31.5mg |
| B vs. L | -1.21 | 0.51 | -1.74 | 0.96 | 0.54 | 1.14 | 0.636 | 0.21 | C: | AVP-825 22mg |
| C vs. L | -1.11 | 0.32 | -1.83 | 945.16 | 0.72 | 945.16 | 0.999 | 0.20 | D: | ROX-828 31.5mg |
| D vs. L | -0.69 | 0.55 | -1.90 | 1860.55 | 1.21 | 1860.55 | 1.00 | 0.20 | E: | Sumatripan 10mg |
| E vs. F | 0.64 | 0.22 | -0.24 | 0.95 | 0.88 | 0.97 | 0.37 | 0.21 | F: | Sumatripan 20mg |
| E vs. L | -0.74 | 0.28 | 0.14 | 0.91 | -0.88 | 0.97 | 0.37 | 0.21 | G: | Zolmitriptan 2.5mg |
| F vs. L | -1.25 | 0.22 | -1.57 | 720.46 | 0.32 | 720.46 | 1.00 | 0.20 | H: | Zolmitriptan 5mg |
| G vs. H | 0.53 | 0.20 | -0.44 | 0.56 | 0.96 | 0.59 | 0.10 | 0.14 | I: | Zavegepant 10mg |
| G vs. L | -1.35 | 0.26 | -0.38 | 0.49 | -0.96 | 0.59 | 0.10 | 0.14 | J: | MAP0004 1mg |
| H vs. L | -1.54 | 0.16 | 0.31 | 773.28 | -1.85 | 773.28 | 1.00 | 0.20 | K: | Butorphanol 1mg |
| I vs. L | -0.52 | 0.18 | -1.97 | 1070.56 | 1.45 | 1070.56 | 1.00 | 0.20 | L: | Placebo |
| J vs. L | -1.30 | 0.27 | -1.70 | 1063.41 | 0.40 | 1063.41 | 1.00 | 0.20 |  |  |
| K vs. L | -1.30 | 0.55 | -1.30 | 1524.42 | 0.00 | 1524.42 | 1.00 | 0.20 |  |  |
| **freedom from nausea at 2 hours** |  |  |  |  |  |  |  |  |  |  |
| A vs. J | -1.60 | 0.69 | -0.38 | 20.59 | -1.22 | 20.60 | 0.95 | 0.00 | A: | DFN-02 10mg |
| B vs. F | -0.51 | 0.72 | 0.40 | 1.17 | -0.91 | 1.31 | 0.49 | 0.00 | B: | Ketorolac 31.5mg |
| B vs. J | -1.08 | 0.69 | -1.99 | 1.23 | 0.91 | 1.31 | 0.49 | 0.00 | C: | ROX-828 31.5mg |
| C vs. J | -0.84 | 0.54 | -3.31 | 140.86 | 2.47 | 140.86 | 0.99 | 0.00 | D: | Sumatripan 10mg |
| D vs. J | 0.22 | 0.17 | -0.69 | 1.30 | 0.91 | 1.31 | 0.49 | 0.00 | E: | Sumatripan 20mg |
| E vs. F | -0.81 | 0.20 | 0.10 | 1.29 | -0.91 | 1.31 | 0.49 | 0.00 | F: | Zolmitriptan 2.5mg |
| E vs. J | -0.99 | 0.19 | -2.80 | 76.31 | 1.81 | 76.31 | 0.98 | 0.00 | G: | Zolmitriptan 5mg |
| F vs. J | 0.21 | 0.14 | 0.46 | 0.32 | -0.25 | 0.35 | 0.47 | 0.00 | H: | Zavegepant 10mg |
| G vs. H | -0.57 | 0.14 | -0.82 | 0.31 | 0.25 | 0.35 | 0.47 | 0.00 | I: | MAP0004 1mg |
| G vs. J | -0.86 | 0.08 | -3.12 | 90.28 | 2.26 | 90.28 | 0.98 | 0.00 | J: | Placebo |
| H vs. J | -0.08 | 0.11 | -3.20 | 89.57 | 3.12 | 89.57 | 0.97 | 0.00 |  |  |
| I vs. J | -0.38 | 0.14 | -3.18 | 91.87 | 2.80 | 91.87 | 0.98 | 0.00 |  |  |
| **freedom from photophobia at 2 hours** |  |  |  |  |  |  |  |  |  |  |
| A vs. J | -1.39 | 0.51 | -0.53 | 20.58 | -0.86 | 20.59 | 0.97 | 0.14 | A: | DFN-02 10mg |
| B vs. E | 0.00 | 0.48 | 0.70 | 0.89 | -0.70 | 1.01 | 0.49 | 0.15 | B: | Ketorolac 31.5mg |
| B vs. J | -0.78 | 0.47 | -1.48 | 0.90 | 0.70 | 1.01 | 0.49 | 0.15 | C: | ROX-828 31.5mg |
| C vs. J | -1.39 | 0.46 | -2.87 | 129.11 | 1.48 | 129.11 | 0.99 | 0.14 | D: | Sumatripan 10mg |
| D vs. E | 0.53 | 0.16 | -0.17 | 1.00 | 0.70 | 1.01 | 0.49 | 0.15 | E: | Sumatripan 20mg |
| D vs. J | -0.60 | 0.19 | 0.10 | 0.99 | -0.70 | 1.01 | 0.49 | 0.15 | F: | Zolmitriptan 2.5mg |
| E vs. J | -1.08 | 0.17 | -2.51 | 74.75 | 1.43 | 74.75 | 0.99 | 0.14 | G: | Zolmitriptan 5mg |
| F vs. G | 0.44 | 0.18 | -0.18 | 0.44 | 0.62 | 0.47 | 0.19 | 0.11 | H: | Zavegepant 10mg |
| F vs. J | -0.92 | 0.19 | -0.29 | 0.43 | -0.62 | 0.47 | 0.19 | 0.11 | I: | MAP0004 1mg |
| G vs. J | -1.17 | 0.13 | -2.63 | 89.95 | 1.46 | 89.95 | 0.99 | 0.14 | J: | Placebo |
| H vs. J | -0.32 | 0.14 | -2.77 | 95.46 | 2.45 | 95.46 | 0.98 | 0.14 |  |  |
| I vs. J | -0.82 | 0.20 | -2.68 | 92.45 | 1.86 | 92.45 | 0.98 | 0.14 |  |  |
| **freedom from phonophobia after 2 hours** |  |  |  |  |  |  |  |  |  |  |
| A vs. J | -1.68 | 0.60 | -0.46 | 20.57 | -1.22 | 20.58 | 0.95 | 0.18 | A: | DFN-02 10mg |
| B vs. E | -0.52 | 0.54 | 0.69 | 0.97 | -1.21 | 1.08 | 0.26 | 0.18 | B: | Ketorolac 31.5mg |
| B vs. J | -0.96 | 0.53 | -2.17 | 0.98 | 1.21 | 1.08 | 0.26 | 0.18 | C: | ROX-828 31.5mg |
| C vs. J | -0.02 | 0.52 | -3.35 | 137.55 | 3.34 | 137.55 | 0.98 | 0.18 | D: | Sumatripan 10mg |
| D vs. E | 0.41 | 0.18 | -0.80 | 1.07 | 1.21 | 1.08 | 0.26 | 0.18 | E: | Sumatripan 20mg |
| D vs. J | -0.64 | 0.20 | 0.57 | 1.06 | -1.21 | 1.08 | 0.27 | 0.18 | F: | Zolmitriptan 2.5mg |
| E vs. J | -0.97 | 0.18 | -3.02 | 74.87 | 2.05 | 74.87 | 0.98 | 0.18 | G: | Zolmitriptan 5mg |
| F vs. G | 0.17 | 0.16 | -0.71 | 0.38 | 0.88 | 0.41 | 0.03 | 0.07 | H: | Zavegepant 10mg |
| F vs. J | -1.23 | 0.17 | -0.35 | 0.36 | -0.88 | 0.41 | 0.03 | 0.07 | I: | MAP0004 1mg |
| G vs. J | -1.15 | 0.16 | -3.13 | 89.91 | 1.98 | 89.91 | 0.98 | 0.18 | J: | Placebo |
| H vs. J | -0.40 | 0.17 | -3.37 | 92.30 | 2.97 | 92.30 | 0.97 | 0.18 |  |  |
| I vs. J | -0.74 | 0.22 | -3.29 | 91.85 | 2.54 | 91.85 | 0.98 | 0.18 |  |  |
| **adverse events** |  |  |  |  |  |  |  |  |  |  |
| A vs. L | -2.35 | 1.51 | -1.10 | 25.03 | -1.25 | 25.08 | 0.96 | 0.25 | A: | DFN-02 10mg |
| B vs. E | -0.30 | 0.54 | -3.04 | 0.98 | 2.74 | 1.10 | 0.01 | 0.20 | B: | Ketorolac 31.5mg |
| B vs. L | -2.83 | 0.54 | -0.09 | 0.98 | -2.74 | 1.10 | 0.01 | 0.20 | C: | AVP-825 22mg |
| C vs. L | -1.94 | 0.41 | -2.76 | 141.51 | 0.81 | 141.51 | 1.00 | 0.25 | D: | Sumatripan 10mg |
| D vs. E | 0.37 | 0.19 | 1.74 | 0.88 | -1.37 | 0.90 | 0.13 | 0.25 | E: | Sumatripan 20mg |
| D vs. L | -0.78 | 0.20 | -2.14 | 0.87 | 1.37 | 0.90 | 0.13 | 0.25 | F: | Zolmitriptan 2.5mg |
| E vs. L | -1.28 | 0.18 | -3.57 | 89.54 | 2.29 | 89.54 | 0.98 | 0.25 | G: | Zolmitriptan 5mg |
| F vs. G | 0.47 | 0.37 | 0.69 | 0.82 | -0.23 | 0.90 | 0.80 | 0.32 | H: | Zavegepant 10mg |
| F vs. L | -0.67 | 0.38 | -0.90 | 0.81 | 0.23 | 0.90 | 0.80 | 0.32 | I: | ROX-828 31.5mg |
| G vs. L | -1.22 | 0.17 | -3.80 | 115.94 | 2.58 | 115.94 | 0.98 | 0.25 | J: | Dihydroergotamine 2mg |
| H vs. L | -0.71 | 0.21 | -3.99 | 141.28 | 3.28 | 141.28 | 0.98 | 0.25 | K: | MAP0004 1mg |
| I vs. L | -0.19 | 0.42 | -4.51 | 200.04 | 4.32 | 200.04 | 0.98 | 0.25 | L: | Placebo |

pain freedom at 1 hour and sustained pain freedom for 24 hours was not potentially inconsistency.

**eTable 12:** Proportion of serious adverse events and the most commonly reported adverse events

| **Medication** | **AEs** | **SAEs** | **treatment-related SAEs** | **withdrawal duo to treatment-related AEs** | **Most commonly reported AEs** |
| --- | --- | --- | --- | --- | --- |
| **Sumatriptan** |  |  |  |  |  |
| DFN-02 | 5 | 0 | 0 | 0 | Dysgeusia (1) |
| AVP-825 | 54 | 0 | 0 | 0 | Dysgeusia (30) |
| Sumatriptan 20mg | 546 | NR | NR | NR | Dysgeusia (241) |
| Sumatriptan 10mg | 298 | NR | NR | NR | Dysgeusia(125) |
| **Zolmitriptan** |  |  |  |  |  |
| Zolmitriptan 2.5mg | 574* | 3 | 1 | 0 | Unusual taste(83*) |
| Zolmitriptan 5.0mg | 2815* | 6 | 0 | 16 | Unusual taste(478) |
| **Zavegepant** |  |  |  |  |  |
| Zavegepant 10mg | 283 | 1 | 0 | 0 | Dysgeusia(182) |
| **Ketorolac** |  |  |  |  |  |
| Ketorolac 31.5mg | 43 | 0 | 0 | 0 | Burning of the nose（32） |
| ROX-828 | 41 | 2 | 0 | 0 | Nasal discomfort(17) |
| **Dihydroergotamine** |  |  |  |  |  |
| Dihydroergotamine 2mg | 84 | 0 | 0 | 1 | Rhinitis (primarily nasal congestion)(44) |
| MAP0004 1mg | 134 | 0 | 0 | 1 | Product taste(32) |
| **opioids** |  |  |  |  |  |
| Butorphanol 1 mg | NR | NR | NR | NR | Dizziness(58) |

Marked with * means reported as frequency of events; Unmarked indicates reported as the number of patients. NR, not reported;

**eTable 13:** League table of pain freedom after 1 hour

| **ROX-828** |  |  |  |  | 3.06 (0.78,12.08) |
| --- | --- | --- | --- | --- | --- |
| 0.71 (0.08,6.15) | **Suma20mg** |  |  |  | 4.33 (0.81,23.10) |
| 0.95 (0.24,3.83) | 1.36 (0.25,7.30) | **Zol5mg** |  |  | **3.20 (2.64,3.88)** |
| 0.82 (0.19,3.58) | 1.16 (0.20,6.72) | 0.86 (0.49,1.51) | **MAP0004** |  | **3.73 (2.19,6.36)** |
| 0.36 (0.03,4.19) | 0.50 (0.04,7.09) | 0.37 (0.05,2.91) | 0.43 (0.05,3.60) | **Buto** | **8.62 (1.11,66.92)** |
| 3.07 (0.79,12.08) | 4.33 (0.81,23.10) | **3.19 (2.64,3.88)** | **3.73 (2.19,6.36)** | **8.62 (1.11,66.92)** | **Placebo** |

Pairwise (upper-right portion) and network (lower-left portion) meta-analysis results are presented as estimated effect sizes for pain freedom after 1 hour. For the result, outcomes are expressed as odds ratios (OR) with 95% credible interval (CI) (OR of > 1 indicated that the treatment specified in the row got more improvement than that specified in the column), 0 < OR < 1, the opposite. For the network meta-analysis, OR of > 1 indicates that the treatment specified in the column got better improvement than that specified in the row, 0 < OR < 1, the opposite. 95% CI that did not contain one was considered to have a statistical difference. Bold results indicate statistical significance. Marked with * indicated a significant difference between direct and mixed comparisons.

DFN-02, sumatriptan nose spray 10 mg with a permeation enhancer; Ket, ketorolac nose spray 31.5mg; Suma10mg, sumatriptan nose spray 10mg; Suma20mg, sumatriptan nose spray 20mg; Zol2.5mg, zolmitriptan nose spray 2.5mg; Zol5mg, zolmitriptan nose spray 5mg; Zave, zavegepant nose spray 10mg; Buto, butorphanol nose spray 1 mg; AVP-825, a drug-device combination of 22mg sumatriptan powder; MAP0004, a dihydroergotamine inhaler; ROX-828, ketorolac 31.5mg with 6% of lidocaine; DHE, dihydroergotamine nose spray 2mg.

**eTable 14:** League table of sustained pain freedom for 24 hours

| **DFN-02** |  |  |  |  |  |  |  | **4.59 (1.41,14.96)** |
| --- | --- | --- | --- | --- | --- | --- | --- | --- |
| 1.18 (0.23,6.00) | **Ket** |  |  | 1.88 (0.71,5.00) |  |  |  | **3.88 (1.28,11.82)** |
| 1.56 (0.36,6.68) | 1.32 (0.32,5.35) | **AVP-825** |  |  |  |  |  | **2.95 (1.26,6.91)** |
| 1.58 (0.36,6.98) | 1.34 (0.32,5.60) | 1.01 (0.29,3.51) | **ROX-828** |  |  |  |  | **2.91 (1.18,7.16)** |
| 2.22 (0.42,11.68) | 1.88 (0.71,5.00) | 1.43 (0.34,6.04) | 1.41 (0.32,6.14) | **Suma20mg** |  |  |  | 2.07 (0.64,6.64) |
| 0.84 (0.24,2.94) | 0.71 (0.22,2.33) | 0.54 (0.21,1.39) | 0.53 (0.20,1.44) | 0.38 (0.11,1.31) | **Zol5mg** |  |  | **5.49 (3.58,8.42)** |
| 2.63 (0.75,9.22) | 2.23 (0.68,7.32) | 1.69 (0.66,4.37) | 1.70 (0.62,4.51) | 1.19 (0.34,4.10) | **3.15 (1.73,5.72)** | **Zave** |  | **1.74 (1.15,2.64)** |
| 1.17 (0.31,4.40) | 0.99 (0.28,3.50) | 0.75 (0.27,2.13) | 0.74 (0.25,2.19) | 0.53 (0.14,1.96) | 1.40 (0.67,2.92) | 0.44 (0.21,0.92) | **MAP0004** | **3.92 (2.16,7.13)** |
| **4.59 (1.41,14.96)** | **3.88 (1.28,11.82)** | **2.95 (1.26,6.91)** | **2.91 (1.18,7.16)** | 2.07 (0.64,6.64) | **5.49 (3.58,8.42)** | **1.74 (1.15,2.64)** | **3.92 (2.16,7.13)** | **Placebo** |

Pairwise (upper-right portion) and network (lower-left portion) meta-analysis results are presented as estimated effect sizes for sustained pain-free for 24 hours. For the result, outcomes are expressed as odds ratios (OR) with 95% credible interval (CI) (OR of > 1 indicated that the treatment specified in the row got more improvement than that specified in the column), 0 < OR < 1, the opposite. For the network meta-analysis, OR of > 1 indicates that the treatment specified in the column got better improvement than that specified in the row, 0 < OR < 1, the opposite. 95% CI that did not contain one was considered to have a statistical difference. Bold results indicates statistical significance. Marked with * indicated a significant difference between direct and mixed comparisons.

DFN-02, sumatriptan nose spray 10 mg with a permeation enhancer; Ket, ketorolac nose spray 31.5mg; Suma10mg, sumatriptan nose spray 10mg; Suma20mg, sumatriptan nose spray 20mg; Zol2.5mg, zolmitriptan nose spray 2.5mg; Zol5mg, zolmitriptan nose spray 5mg; Zave, zavegepant nose spray 10mg; Buto, butorphanol nose spray 1 mg; AVP-825, a drug-device combination of 22mg sumatriptan powder; MAP0004, a dihydroergotamine inhaler; ROX-828, ketorolac 31.5mg with 6% of lidocaine; DHE, dihydroergotamine nose spray 2mg.

**eTable 15:** League table of freedom from nausea at 2 hours

| **DFN-02** |  |  |  |  |  |  |  |  | **4.95 (1.29,19.01)** |
| --- | --- | --- | --- | --- | --- | --- | --- | --- | --- |
| 1.40 (0.22,8.72) | **Ket** |  |  | 1.66 (0.41,6.79) |  |  |  |  | *2.95 (0.77,11.34) |
| 2.13 (0.39,11.78) | 1.59 (0.30,7.79) | **ROX-828** |  |  |  |  |  |  | 2.32 (0.81,6.65) |
| 2.26 (0.56,9.17) | 1.62 (0.46,5.77) | 1.06 (0.35,3.26) | **Suma10mg** | 0.80 (0.58,1.11) |  |  |  |  | **2.27 (1.54,3.36)** |
| 1.84 (0.46,7.42) | 1.32 (0.38,4.59) | 0.86 (0.28,2.63) | 0.81 (0.59,1.12) | **Suma20mg** |  |  |  |  | **2.67 (1.85,3.88)** |
| 2.68 (0.68,10.53) | 1.92 (0.54,6.81) | 1.26 (0.43,3.71) | 1.18 (0.75,1.87) | 1.46 (0.93,2.28) | **Zol2.5mg** | 0.81 (0.62,1.07) |  |  | **1.77 (1.34,2.34)** |
| 2.09 (0.54,8.10) | 1.50 (0.43,5.23) | 0.98 (0.34,2.84) | 0.92 (0.61,1.40) | 1.14 (0.76,1.70) | 0.78 (0.61,1.01) | **Zol5mg** |  |  | **2.36 (2.01,2.78)** |
| **4.57 (1.17,17.85)** | 3.27 (0.93,11.54) | 2.14 (0.73,6.28) | **2.02 (1.30,3.14)** | **2.48 (1.61,3.82)** | **1.71 (1.22,2.39)** | **2.19 (1.67,2.87)** | **Zave** |  | 1.08 (0.87,1.39) |
| 3.40 (0.86,13.45) | 2.43 (0.68,8.70) | 1.59 (0.54,4.74) | 1.50 (0.93,2.42) | **1.85 (1.16,2.95)** | 1.27 (0.87,1.86) | **1.63 (1.18,2.25)** | 0.75 (0.52,1.07) | **MAP0004** | **1.48 (1.10,1.95)** |
| **4.95 (1.29,19.01)** | ***3.55 (1.02,12.27)** | 2.32 (0.81,6.65) | **2.19 (1.49,3.21)** | **2.69 (1.86,3.90)** | **1.85 (1.43,2.39)** | **2.37 (2.02,2.78)** | 1.08 (0.87,1.35) | **1.46 (1.10,1.93)** | **Placebo** |

Pairwise (upper-right portion) and network (lower-left portion) meta-analysis results are presented as estimated effect sizes for freedom from nausea at 2 hours. For the result, outcomes are expressed as odds ratios (OR) with 95% credible interval (CI) (OR of > 1 indicated that the treatment specified in the row got more improvement than that specified in the column), 0 < OR < 1, the opposite. For the network meta-analysis, OR of > 1 indicates that the treatment specified in the column got better improvement than that specified in the row, 0 < OR < 1, the opposite. 95% CI that did not contain one was considered to have a statistical difference. Bold results indicate statistical significance. Marked with * indicated a significant difference between direct and mixed comparisons.

DFN-02, sumatriptan nose spray 10 mg with a permeation enhancer; Ket, ketorolac nose spray 31.5mg; Suma10mg, sumatriptan nose spray 10mg; Suma20mg, sumatriptan nose spray 20mg; Zol2.5mg, zolmitriptan nose spray 2.5mg; Zol5mg, zolmitriptan nose spray 5mg; Zave, zavegepant nose spray 10mg; Buto, butorphanol nose spray 1 mg; AVP-825, a drug-device combination of 22mg sumatriptan powder; MAP0004, a dihydroergotamine inhaler; ROX-828, ketorolac 31.5mg with 6% of lidocaine; DHE, dihydroergotamine nose spray 2mg.

**eTable 16:** League table of freedom from photophobia at 2 hours

| **DFN-02** |  |  |  |  |  |  |  |  | **4.00 (1.48,10.83)** |
| --- | --- | --- | --- | --- | --- | --- | --- | --- | --- |
| 1.58 (0.44,5.71) | **Ket** |  |  | 1.00 (0.40,2.52) |  |  |  |  | *2.18 (0.88,5.40) |
| 0.99 (0.26,3.77) | 0.63 (0.19,2.09) | **ROX-828** |  |  |  |  |  |  | **4.03 (1.66,9.81)** |
| 2.25 (0.78,6.44) | 1.42 (0.61,3.30) | 2.26 (0.87,5.87) | **Suma10mg** | **0.60 (0.44,0.80)** |  |  |  |  | **1.81 (1.27,2.56)** |
| 1.35 (0.47,3.86) | 0.85 (0.38,1.92) | 1.36 (0.53,3.48) | **0.60 (0.45,0.81)** | **Suma20mg** |  |  |  |  | **2.95 (2.16,4.03)** |
| 1.78 (0.62,5.11) | 1.13 (0.47,2.71) | 1.79 (0.69,4.65) | 0.79 (0.49,1.29) | 1.32 (0.83,2.10) | **Zol2.5mg** | **0.64 (0.45,0.92)** |  |  | **2.50 (1.71,3.67)** |
| 1.25 (0.45,3.48) | 0.79 (0.34,1.84) | 1.26 (0.50,3.16) | **0.56 (0.37,0.84)** | 0.93 (0.63,1.37) | **0.70 (0.50,0.98)** | **Zol5mg** |  |  | **3.20 (2.54,4.03)** |
| **2.90 (1.04,8.20)** | 1.83 (0.78,4.29) | **2.92 (1.16,7.38)** | 1.29 (0.84,1.98) | **2.15 (1.44,3.23)** | **1.63 (1.06,2.51)** | **2.32 (1.64,3.28)** | **Zave** |  | **1.38 (1.06,1.79)** |
| 1.76 (0.61,5.08) | 1.11 (0.46,2.70) | 1.77 (0.68,4.63) | 0.78 (0.47,1.29) | 1.30 (0.81,2.11) | 0.99 (0.60,1.63) | 1.41 (0.91,2.17) | **0.61 (0.39,0.95)** | **MAP0004** | **2.28 (1.58,3.29)** |
| **4.00 (1.48,10.83)** | ***2.53 (1.13,5.69)** | **4.03 (1.66,9.81)** | **1.78 (1.27,2.51)** | **2.97 (2.18,4.05)** | **2.25 (1.59,3.17)** | **3.20 (2.54,4.03)** | **1.38 (1.06,1.79)** | **2.28 (1.58,3.29)** | **Placebo** |

Pairwise (upper-right portion) and network (lower-left portion) meta-analysis results are presented as estimated effect sizes for freedom from photophobia at 2 hours. For the result, outcomes are expressed as odds ratios (OR) with 95% credible interval (CI) (OR of > 1 indicated that the treatment specified in the row got more improvement than that specified in the column), 0 < OR < 1, the opposite. For the network meta-analysis, OR of > 1 indicates that the treatment specified in the column got better improvement than that specified in the row, 0 < OR < 1, the opposite. 95% CI that did not contain one was considered to have a statistical difference. Bold results indicate statistical significance. Marked with * indicated a significant difference between direct and mixed comparisons.

DFN-02, sumatriptan nose spray 10 mg with a permeation enhancer; Ket, ketorolac nose spray 31.5mg; Suma10mg, sumatriptan nose spray 10mg; Suma20mg, sumatriptan nose spray 20mg; Zol2.5mg, zolmitriptan nose spray 2.5mg; Zol5mg, zolmitriptan nose spray 5mg; Zave, zavegepant nose spray 10mg; Buto, butorphanol nose spray 1 mg; AVP-825, a drug-device combination of 22mg sumatriptan powder; MAP0004, a dihydroergotamine inhaler; ROX-828, ketorolac 31.5mg with 6% of lidocaine; DHE, dihydroergotamine nose spray 2mg.

**eTable 17:** League table of freedom from phonophobia after 2 hours

| **DFN-02** |  |  |  |  |  |  |  |  | **5.36 (1.67,17.22)** |
| --- | --- | --- | --- | --- | --- | --- | --- | --- | --- |
| 1.60 (0.36,7.18) | **Ket** |  |  | 1.68 (0.58,4.87) |  |  |  |  | *2.61 (0.91,7.43) |
| **5.28 (1.12,24.98)** | 3.31 (0.82,13.37) | **ROX-828** |  |  |  |  |  |  | 1.02 (0.36,2.83) |
| 2.96 (0.87,10.14) | 1.86 (0.69,4.98) | 0.56 (0.19,1.68) | **Suma10mg** | **0.67 (0.47,0.95)** |  |  |  |  | **1.88 (1.26,2.80)** |
| 2.03 (0.60,6.90) | 1.28 (0.49,3.30) | 0.39 (0.13,1.14) | **0.69 (0.48,0.98)** | **Suma20mg** |  |  |  |  | **2.63 (1.84,3.77)** |
| 1.79 (0.52,6.22) | 1.12 (0.40,3.18) | 0.34 (0.11,1.03) | 0.61 (0.34,1.08) | 0.88 (0.51,1.54) | **Zol2.5mg** | 0.84 (0.54,1.32) |  |  | **3.42 (2.14,5.46)** |
| 1.70 (0.51,5.69) | 1.07 (0.39,2.89) | **0.32 (0.11,0.94)** | **0.57 (0.35,0.94)** | 0.84 (0.52,1.34) | 0.95 (0.62,1.44) | **Zol5mg** |  |  | **3.15 (2.32,4.26)** |
| **3.59 (1.07,12.11)** | 2.25 (0.82,6.15) | 0.68 (0.23,2.01) | 1.21 (0.72,2.03) | **1.7 (1.08,2.89)** | **2.00 (1.16,3.45)** | **2.11 (1.34,3.32)** | **Zave** |  | **1.49 (1.06,2.09)** |
| 2.54 (0.73,8.84) | 1.59 (0.56,4.52) | 0.48 (0.16,1.47) | 0.86 (0.48,1.54) | 1.25 (0.71,2.20) | 1.42 (0.77,2.61) | 1.49 (0.88,2.54) | 0.71 (0.41,1.23) | **MAP0004** | **2.11 (1.36,3.26)** |
| **5.36 (1.67,17.22)** | ***3.36 (1.30,8.66)** | 1.02 (0.36,2.83) | **1.81 (1.23,2.67)** | **2.63 (1.84,3.77)** | **2.99 (1.95,4.58)** | **3.15 (2.32,4.26)** | **1.49 (1.06,2.09)** | **2.11 (1.36,3.26)** | **Placebo** |

Pairwise (upper-right portion) and network (lower-left portion) meta-analysis results are presented as estimated effect sizes for freedom from phonophobia at 2 hours. For the result, outcomes are expressed as odds ratios (OR) with 95% credible interval (CI) (OR of > 1 indicated that the treatment specified in the row got more improvement than that specified in the column), 0 < OR < 1, the opposite. For the network meta-analysis, OR of > 1 indicates that the treatment specified in the column got better improvement than that specified in the row, 0 < OR < 1, the opposite. 95% CI that did not contain one was considered to have a statistical difference. Bold results indicate statistical significance. Marked with * indicated a significant difference between direct and mixed comparisons.

DFN-02, sumatriptan nose spray 10 mg with a permeation enhancer; Ket, ketorolac nose spray 31.5mg; Suma10mg, sumatriptan nose spray 10mg; Suma20mg, sumatriptan nose spray 20mg; Zol2.5mg, zolmitriptan nose spray 2.5mg; Zol5mg, zolmitriptan nose spray 5mg; Zave, zavegepant nose spray 10mg; Buto, butorphanol nose spray 1 mg; AVP-825, a drug-device combination of 22mg sumatriptan powder; MAP0004, a dihydroergotamine inhaler; ROX-828, ketorolac 31.5mg with 6% of lidocaine; DHE, dihydroergotamine nose spray 2mg.

**eTable 18:** Sensitivity analysis 1 and 2 of pain freedom at 2 hours

| **DFN-02** | 0.75 (0.22,2.49) | 1.01 (0.32,3.14) | 1.34 (0.34,5.29) | 1.45 (0.50,4.16) | 0.86 (0.31,2.38) | NA | NA | 1.57 (0.60,4.13) | 0.75 (0.27,2.09) | 0.73 (0.18,2.90) | **^2.67 (1.05,6.83)** |
| --- | --- | --- | --- | --- | --- | --- | --- | --- | --- | --- | --- |
| 0.70 (0.19,2.56) | **Ket** | 1.33 (0.49,3.60) | 1.79 (0.51,6.23) | 1.92 (0.86,4.31) | 1.15 (0.55,2.37) | NA | NA | 2.09 (0.960,4.58) | 1.00 (0.43,2.34) | 0.97 (0.27,3.42) | **3.56 (1.68,7.52)** |
| 0.88 (0.27,2.87) | 1.26 (0.45,3.50) | **AVP-825** | 1.34 (0.40,4.42) | 1.44 (0.63,3.26) | 0.86 (0.40,1.84) | NA | NA | 1.57 (0.78,3.13) | 0.75 (0.35,1.62) | 0.72 (0.21,2.42) | **2.66 (1.38,5.13)** |
| 1.35 (0.31,5.80) | 1.91 (0.50,7.31) | 1.52 (0.44,5.20) | **ROX-828** | 1.07 (0.35,3.27) | 0.64 (0.22,1.87) | NA | NA | 1.17 (0.42,3.26) | 0.56 (0.19,1.64) | 0.54 (0.13,2.24) | 1.99 (0.73,5.39) |
| 1.41 (0.46,4.30) | 2.00 (0.83,4.77) | 1.59 (0.72,3.50) | 1.04 (0.32,3.36) | **Suma10mg** | **0.59 (0.40,0.87)** | NA | NA | 1.08 (0.63,1.86) | **0.52 (0.28,0.97)** | 0.50 (0.16,1.54) | **1.84 (1.13,3.00)** |
| 0.77 (0.26,2.28) | 1.10 (0.49,2.42) | 0.87 (0.41,1.84) | 0.57 (0.18,1.79) | **0.54 (0.36,0.82)** | **Suma 20mg** | NA | NA | **1.82 (1.16,2.86)** | 0.87 (0.50,1.52) | 0.84 (0.28,2.50) | **3.09 (2.09,4.57)** |
| 0.87 (0.29,2.63) | 1.24 (0.48,3.16) | 0.98 (0.45,2.13) | 0.64 (0.20,2.06) | 0.61 (0.31,1.22) | 1.12 (0.60,2.11) | **Zol2.5mg** | NA | NA | NA | NA | NA |
| 0.58 (0.20,1.63) | 0.82 (0.34,1.94) | 0.65 (0.33,1.28) | 0.42 (0.14,1.28) | **0.41 (0.23,0.72)** | 0.75 (0.45,1.24) | 0.66 (0.42,1.03) | **Zol5mg** | NA | NA | NA | NA |
| 1.59 (0.55,4.58) | 2.26 (0.93,5.47) | 1.79 (0.88,3.64) | 1.18 (0.38,3.60) | 1.13 (0.62,2.06) | **2.06 (1.20,3.54)** | **1.82 (1.02,3.25)** | **2.74 (1.76,4.27)** | **Zave** | **0.48 (0.30,0.75)** | 0.46 (0.16,1.30) | **1.69 (1.357,2.12)** |
| 0.73 (0.23,2.25) | 1.03 (0.39,2.72) | 0.82 (0.36,1.84) | 0.54 (0.16,1.76) | 0.51 (0.25,1.06) | 0.94 (0.48,1.84) | 0.83 (0.41,1.68) | 1.26 (0.69,2.27) | **0.45 (0.24,0.85)** | **MAP0004** | 0.96 (0.32,2.85) | **3.53 (2.38,5.23)** |
| NA | NA | NA | NA | NA | NA | NA | NA | NA | NA | **Buto** | **3.67 (1.33,10.11)** |
| 2.67 (0.98,7.31) | **3.81 (1.68,8.61)** | **3.03 (1.63,5.62)** | 1.99 (0.68,5.75) | **1.90 (1.16,3.11)** | **3.47 (2.28,5.28)** | **3.07 (1.93,4.90)** | **4.63 (3.50,6.11)** | **1.68 (1.19,2.37)** | **3.67 (2.18,6.18)** | NA | **Placebo** |

Sensitivity analysis 1 (lower-left portion) and Sensitivity analysis 2 (upper-right portion) meta-analysis results. For the result, outcomes are expressed as odds ratios (OR) with 95% credible interval (CI) (OR of > 1 indicated that the treatment specified in the row got more improvement than that specified in the column), 0 < OR < 1, the opposite. For the network meta-analysis, OR of > 1 indicated that the treatment specified in the column got better improvement than that specified in the row, 0 < OR < 1, the opposite. 95% CI that did not contain one was considered to have a statistical difference.

Bold results indicated statistical significance. Marked with ^ indicated a significant difference between the basic model and sensitivity analysis.

DFN-02, sumatriptan nose spray 10 mg with a permeation enhancer; Ket, ketorolac nose spray 31.5mg; Suma10mg, sumatriptan nose spray 10mg; Suma20mg, sumatriptan nose spray 20mg; Zol2.5mg, zolmitriptan nose spray 2.5mg; Zol5mg, zolmitriptan nose spray 5mg; Zave, zavegepant nose spray 10mg; Buto, butorphanol nose spray 1 mg; AVP-825, a drug-device combination of 22mg sumatriptan powder; MAP0004, a dihydroergotamine inhaler; ROX-828, ketorolac 31.5mg with 6% of lidocaine; DHE, dihydroergotamine nose spray 2mg.

**eTable 19:** Sensitivity analysis 3 of pain freedom at 2 hours

| **DFN-02** |  |  |  |  |  |  |  |  |  |
| --- | --- | --- | --- | --- | --- | --- | --- | --- | --- |
| 0.89 (0.30,2.65) | **AVP-825** |  |  |  |  |  |  |  |  |
| 1.34 (0.34,5.29) | 1.50 (0.48,4.70) | **ROX-828** |  |  |  |  |  |  |  |
| 1.36 (0.48,3.83) | 1.52 (0.75,3.08) | 1.01 (0.34,3.01) | **Suma10mg** |  |  |  |  |  |  |
| 0.74 (0.27,2.05) | 0.83 (0.42,1.63) | 0.55 (0.19,1.61) | **0.54 (0.39,0.75)** | **Suma20mg** |  |  |  |  |  |
| 0.47 (0.17,1.31) | 0.53 (0.26,1.05) | 0.35 (0.12,1.03) | **0.34 (0.19,0.63)** | 0.63(0.36,1.11) | **Zol5mg** |  |  |  |  |
| 1.57 (0.60,4.13) | 1.76 (0.96,3.20) | 1.17 (0.42,3.26) | 1.15 (0.70,1.88) | **2.11 (1.35,3.30)** | **3.31 (2.09,5.26)** | **Zave** |  |  |  |
| 0.74 (0.26,2.04) | 0.82 (0.42,1.62) | 0.55 (0.18,1.60) | **^0.54 (0.30,0.97)** | 0.99 (0.57,1.71) | 1.55 (0.89,2.72) | **0.47 (0.30,0.73)** | **MAP0004** |  |  |
| 0.73 (0.18,2.90) | 0.81 (0.25,2.58) | 0.54 (0.13,2.24) | 0.53 (0.17,1.61) | 0.97 (0.33,2.89) | 1.53 (0.51,4.57) | 0.46 (0.16,1.30) | 0.98 (0.33,2.91) | **Buto** |  |
| **^2.67 (1.05,6.83)** | **2.99 (1.71,5.20)** | 1.99 (0.73,5.39) | **1.96 (1.26,3.03)** | **3.59 (2.44,5.28)** | **5.63 (3.75,8.43)** | **1.69 (1.35,2.12)** | **3.61 (2.45,5.32)** | **3.67 (1.33,10.11)** | **Placebo** |

For the result, outcomes are expressed as odds ratios (OR) with 95% credible interval (CI) (OR of > 1 indicated that the treatment specified in the row got more improvement than that specified in the column), 0 < OR < 1, the opposite. For the network meta-analysis, OR of > 1 indicated that the treatment specified in the column got better improvement than that specified in the row, 0 < OR < 1, the opposite. 95% CI that did not contain one was considered to have a statistical difference. Bold results indicated statistical significance. Marked with ^ indicated a significant difference between the basic model and sensitivity analysis.

DFN-02, sumatriptan nose spray 10 mg with a permeation enhancer; Ket, ketorolac nose spray 31.5mg; Suma10mg, sumatriptan nose spray 10mg; Suma20mg, sumatriptan nose spray 20mg; Zol2.5mg, zolmitriptan nose spray 2.5mg; Zol5mg, zolmitriptan nose spray 5mg; Zave, zavegepant nose spray 10mg; Buto, butorphanol nose spray 1 mg; AVP-825, a drug-device combination of 22mg sumatriptan powder; MAP0004, a dihydroergotamine inhaler; ROX-828, ketorolac 31.5mg with 6% of lidocaine; DHE, dihydroergotamine nose spray 2mg.

**eTable 20:** Sensitivity analysis 1 and 2 of adverse events

| **DFN-02** | 1.04 (0.05,23.76) | 1.38 (0.06,31.07) | 8.71 (0.40,190.6) | 3.93 (0.20,79.06) | 2.34 (0.11,46.83) | NA | NA | 5.17 (0.26,103.7) | 7.81 (0.38,161.6) | 10.51 (0.54,204.9) |
| --- | --- | --- | --- | --- | --- | --- | --- | --- | --- | --- |
| 1.12 (0.05,25.61) | **Ket** | 1.33 (0.34,5.18) | **8.38 (2.29,30.68)** | **3.78 (1.33,10.72)** | 2.26 (0.84,6.06) | NA | NA | **4.97 (1.70,14.59)** | **7.52 (2.37,23.88)** | **10.12 (3.77,27.19)** |
| 1.51 (0.07,32.82) | 1.35 (0.37,4.87) | **AVP-825** | **6.30 (1.79,22.14)** | **2.84 (1.01,7.98)** | 1.70 (0.62,4.67) | NA | NA | **3.74 (1.34,10.43)** | **5.65 (1.86,17.15)** | **7.61 (2.99,19.36)** |
| 8.71 (0.40,190.4) | **7.77 (2.12,28.47)** | **5.77 (1.79,18.60)** | **ROX-828** | 0.45 (0.18,1.17) | **0.27 (0.11,0.68)** | NA | NA | 0.59 (0.23,1.52) | 0.90 (0.32,2.52) | 1.21 (0.52,2.80) |
| 3.88 (0.19,78.78) | **3.47 (1.20,10.00)** | ^2.57 (0.99,6.70) | 0.45 (0.17,1.18) | **Suma10mg** | **0.60 (0.39,0.91)** | NA | NA | 1.32 (0.71,2.43) | 1.99 (0.95,4.18) | **2.68 (1.73,4.15)** |
| 2.72 (0.14,54.68) | 2.43 (0.90,6.55) | 1.80 (0.72,4.54) | **0.31 (0.12,0.80)** | ^0.70 (0.44,1.12) | **Suma20mg** | NA | NA | **2.21 (1.24,3.93)** | **3.33 (1.63,6.82)** | **4.49 (3.04,6.63)** |
| 5.15 (0.25,106.09) | **4.60 (1.46,14.50)** | **3.41 (1.25,9.30)** | 0.59 (0.21,1.64) | 1.33 (0.62,2.85) | 1.89 (0.92,3.89) | **Zol2.5mg** | NA | NA | NA | NA |
| 3.11 (0.16,61.80) | 2.78 (0.98,7.92) | 2.06 (0.85,4.99) | **0.36 (0.15,0.88)** | 0.80 (0.44,1.47) | 1.14 (0.66,1.97) | 0.61 (0.34,1.07) | **Zol5mg** | NA | NA | NA |
| 5.17 (0.26,103.7) | **4.61 (1.57,13.55)** | **3.42 (1.36,8.59)** | 0.59 (0.23,1.52) | 1.33 (0.69,2.56) | **1.90 (1.04,3.47)** | 1.00 (0.49,2.06) | 1.66 (0.97,2.85) | **Zave** | 1.51 (0.73,3.15) | **2.04 (1.33,3.12)** |
| 7.51 (0.37,154.2) | **6.70 (2.14,20.97)** | **4.97 (1.84,13.43)** | 0.86 (0.31,2.37) | 1.93 (0.91,4.11) | **2.76 (1.36,5.61)** | 1.46 (0.65,3.28) | **2.41 (1.25,4.66)** | 1.45 (0.72,2.95) | **MAP0004** | 1.35 (0.74,2.45) |
| 10.52 (0.54,204.8) | **9.39 (3.49,25.29)** | **6.97 (3.08,15.77)** | 1.21 (0.52,2.80) | **2.71 (1.65,4.46)** | **3.86 (2.52,5.93)** | **2.04 (1.14,3.65)** | **3.38 (2.41,4.74)** | **2.04 (1.33,3.11)** | 1.40 (0.80,2.47) | **Placebo** |

Sensitivity analysis 1 (lower-left portion) and Sensitivity analysis 2 (upper-right portion) meta-analysis results. For the result, outcomes are expressed as odds ratios (OR) with 95% credible interval (CI) (OR of > 1 indicated that the treatment specified in the row got more improvement than that specified in the column), 0 < OR < 1, the opposite. For the network meta-analysis, OR of > 1 indicated that the treatment specified in the column got better improvement than that specified in the row, 0 < OR < 1, the opposite. 95% CI that did not contain one was considered to have a statistical difference. Bold results indicated statistical significance. Marked with ^ indicated a significant difference between the basic model and sensitivity analysis.

DFN-02, sumatriptan nose spray 10 mg with a permeation enhancer; Ket, ketorolac nose spray 31.5mg; Suma10mg, sumatriptan nose spray 10mg; Suma20mg, sumatriptan nose spray 20mg; Zol2.5mg, zolmitriptan nose spray 2.5mg; Zol5mg, zolmitriptan nose spray 5mg; Zave, zavegepant nose spray 10mg; Buto, butorphanol nose spray 1 mg; AVP-825, a drug-device combination of 22mg sumatriptan powder; MAP0004, a dihydroergotamine inhaler; ROX-828, ketorolac 31.5mg with 6% of lidocaine; DHE, dihydroergotamine nose spray 2mg.

**eTable 21:** Sensitivity analysis 3 of adverse events.

| **DFN-02** |  |  |  |  |  |  |  |  |  |
| --- | --- | --- | --- | --- | --- | --- | --- | --- | --- |
| 1.51 (0.07,32.87) | **AVP-825** |  |  |  |  |  |  |  |  |
| 8.71 (0.40,190.7) | **5.77 (1.78,18.66)** | **ROX-828** |  |  |  |  |  |  |  |
| 4.89 (0.24,97.84) | **3.24 (1.30,8.05)** | 0.56 (0.22,1.43) | **Suma10mg** |  |  |  |  |  |  |
| 3.40 (0.17,67.90) | 2.25 (0.91,5.55) | **0.39 (0.16,0.98)** | ^0.70 (0.48,1.01) | **Suma20mg** |  |  |  |  |  |
| 2.79 (0.14,57.64) | 1.85 (0.67,5.08) | **0.32 (0.11,0.90)** | 0.57 (0.28,1.17) | 0.82 (0.41,1.66) | **Zol5mg** |  |  |  |  |
| 5.17 (0.26,103.8) | **3.42 (1.36,8.61)** | 0.59 (0.23,1.53) | 1.06 (0.59,1.89) | 1.52 (0.86,2.69) | 1.85 (0.89,3.84) | **Zave** |  |  |  |
| 1.09 (0.05,23.71) | 0.72 (0.23,2.29) | **0.13 (0.04,0.40)** | **0.22 (0.09,0.55)** | **0.32 (0.13,0.79)** | ^0.39 (0.14,1.07) | **0.21 (0.08,0.53)** | **DHE2mg** |  |  |
| 7.50 (0.37,154.3) | **4.97 (1.83,13.47)** | 0.86 (0.31,2.38) | 1.54 (0.77,3.07) | **2.21 (1.11,4.37)** | **2.69 (1.18,6.12)** | 1.45 (0.71,2.96) | **6.89 (2.55,18.64)** | **MAP0004** |  |
| 10.52 (0.54,204.9) | **6.97 (3.07,15.79)** | 1.21 (0.52,2.80) | **2.15 (1.45,3.20)** | **3.09 (2.12,4.51)** | **3.77 (2.09,6.82)** | **2.04 (1.33,3.12)** | **9.65 (4.27,21.85)** | 1.40 (0.79,2.48) | **Placebo** |

For the result, outcomes are expressed as odds ratios (OR) with 95% credible interval (CI) (OR of > 1 indicated that the treatment specified in the row got more improvement than that specified in the column), 0 < OR < 1, the opposite. For the network meta-analysis, OR of > 1 indicated that the treatment specified in the column got better improvement than that specified in the row, 0 < OR < 1, the opposite. 95% CI that did not contain one was considered to have a statistical difference. Bold results indicated statistical significance. Marked with ^ indicated a significant difference between the basic model and sensitivity analysis.

DFN-02, sumatriptan nose spray 10 mg with a permeation enhancer; Ket, ketorolac nose spray 31.5mg; Suma10mg, sumatriptan nose spray 10mg; Suma20mg, sumatriptan nose spray 20mg; Zol2.5mg, zolmitriptan nose spray 2.5mg; Zol5mg, zolmitriptan nose spray 5mg; Zave, zavegepant nose spray 10mg; Buto, butorphanol nose spray 1 mg; AVP-825, a drug-device combination of 22mg sumatriptan powder; MAP0004, a dihydroergotamine inhaler; ROX-828, ketorolac 31.5mg with 6% of lidocaine; DHE, dihydroergotamine nose spray 2mg.

**eFigure 1:** Overview of risk of bias

**eFigure 2:** Detailed risk of bias in each study

**eFigure 3:** Funnel plot and Egger-value results of all studies included for all endpoints


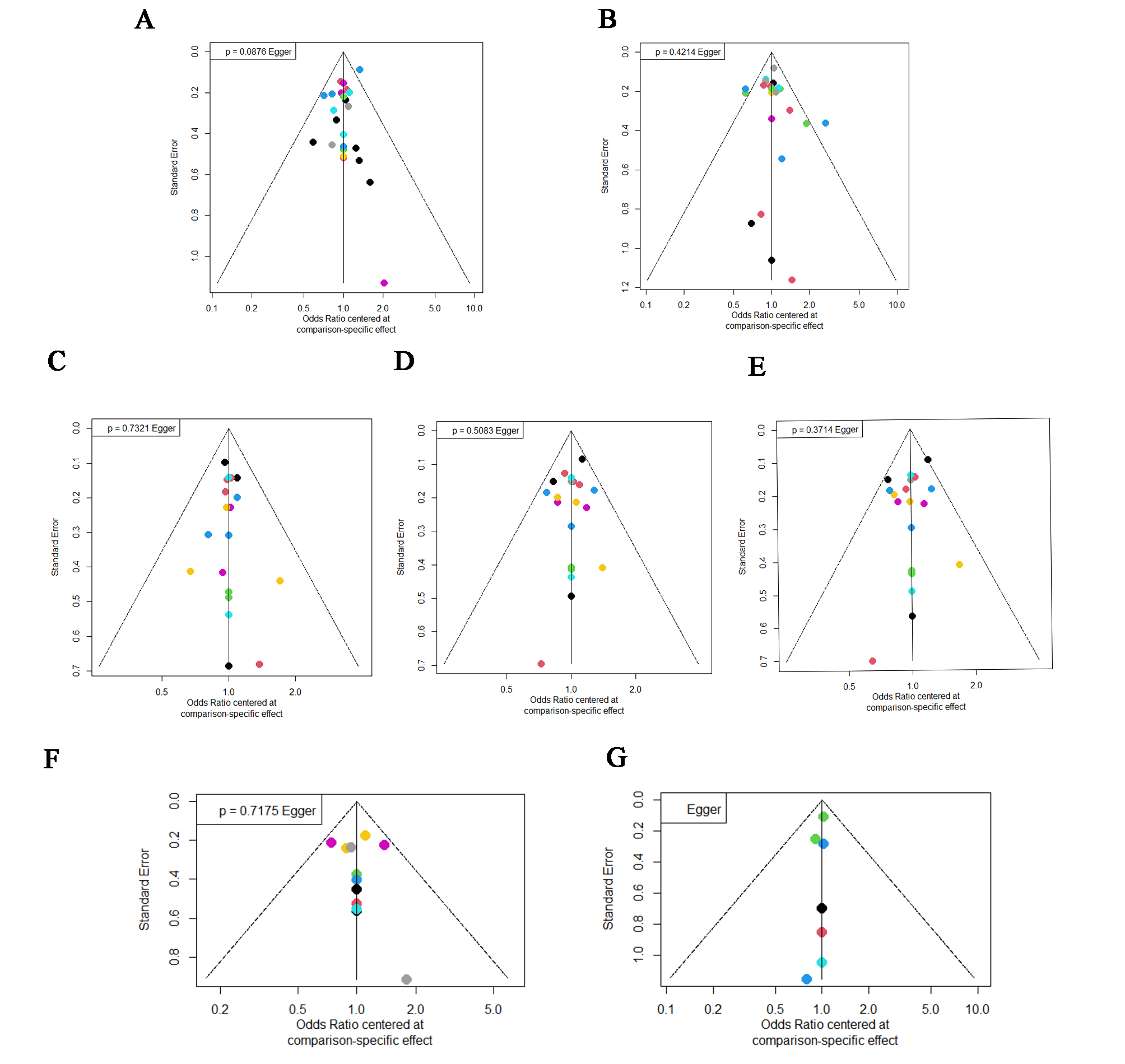


Primary endpoints, (A): pain freedom at 2 hours. (B): adverse events. Secondary endpoints, (C): freedom from nausea at 2 hours. (D): freedom from photophobia at 2 hours. (E): freedom from phonophobia at 2 hours. (F): sustained pain freedom for 24 hours. (G): pain freedom at 1 hour. For outcome indicators of G (pain freedom at 1 hour), Egger-value was not reported due to the fewer comparisons available, but the funnel plot was more balanced so we consider the publication bias to be slight.
